# Functionalized *C*_3_-Symmetric Building Blocks—The Chemistry of Triaminotrimesic Acid

**DOI:** 10.3390/molecules27144369

**Published:** 2022-07-07

**Authors:** Lisa Schmidt, Danny Wagner, Martin Nieger, Stefan Bräse

**Affiliations:** 1 Institute of Organic Chemistry, Karlsruhe Institute of Technology (KIT), Fritz-Haber-Weg 6, 76131 Karlsruhe, Germany; lisa.schmidt@kit.edu (L.S.); danny.wagner2@kit.edu (D.W.); 2 Department of Chemistry, University of Helsinki, P.O. Box 55 (A. I. Virtasen aukio 1), FIN-00014 Helsinki, Finland; martin.nieger@helsinki.fi; 3 Institute of Biological and Chemical Systems—FMS, Karlsruhe Institute of Technology (KIT), Hermann-Von-Helmholtz-Platz 1, 76344 Leopoldshafen, Germany

**Keywords:** azides, click reactions, *C*_3_-symmetrical building blocks, carboxylic acids, X-ray

## Abstract

A series of *C*_3_-symmetric fully substituted benzenes were prepared based on alkyl triamino-benzene-tricarboxylates. Starting with a one step-synthesis, the alkyl triamino-benzene-tricarboxylates were synthesized using the corresponding cyanoacetates. The reactivity of these electronically sophisticated compounds was investigated by the formation of azides, the click reaction of the azides and a Sandmeyer-like reaction. Caused by the low stability of triaminobenzenes, direct *N*-alkylation was rarely reported. The use of the stable alkyl triamino-benzene-tricarboxylates allowed us total *N*-alkylation under standard alkylation conditions. The molecular structures of the *C*_3_-symmetric structures have been corroborated by an X-ray analysis.

## 1. Introduction

**Figure 1:**
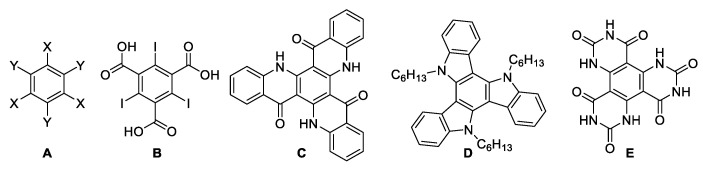
Generic structure of *C*_3_-symmetric benzenes (**A**), 2,4,6-triiodobenzene-1,3,5-tricarboxylic acid (**B**), [34] two prominent *C*_3h_-symmetric disk-like structures triquinolonobenzene (TQB) (**C**) [9] and 5,10,15-trihexyl-10,15-dihydro-5H-diindolo [3,2-a:3′,2′-c] carbazole (**D**); precursor dipyrimido [4,5-f:4′,5′-h] quinazoline-2,4,6,8,10,12-(1H,3H,5H,7H,-9H,11H)-hexone (**E**) [35].

The symmetry of molecular building blocks plays a pivotal role in the overall geometry of the materials that are formed. Besides many examples of *C*_2_-symmetric [1] building blocks, the *C*_3_-symmetrical ones have found fewer applications, other than in life sciences [2] or material sciences. However, *C*_3_-symmetrical-based geometries can be found in star-shaped molecules, dendrimers, and molecular cages [3], thus allowing the formation of columnar structuring due to strong π-π interactions in the case of appropriately functionalized monomers [4] (for reviews, see Ref. [5]). The applications range from discotic liquid crystals [6,7]; mesogens [8]; OLED emitters featuring thermally-activated delayed fluorescence; [9,10,11] gels; [12] metal-organic frameworks (MOFs) (trimesic acid/BTC: MOF-177 [13]; MOF-199; 2D-covalent organic frameworks (COFs) [14,15]; hole-transporting materials for photovoltaics; [16] materials for second harmonic generation/non-linear optics by generating octopoles [17,18,19]; hydrogels [20,21] and many more [22]. Noteworthy in this context are functionalized truxenes [23,24] such as truxenones [25,26,27], or triazatruxenes [28,29] (e.g., D, Figure 1), which exhibit outstanding photophysical properties [30,31,32,33].

Fully substituted derivatives of type **A** (X, Y are non-hydrogen atoms, Figure 1) also exhibit additional properties due to steric crowding. The non-planarity of some derivatives leads to different functionalization of the side groups, e.g., carboxylic acids (Figure 1, Structure **B**) [34].

The synthesis of such molecules can either start from condensation reactions (mostly carbonyl compounds in aldol-like reactions), the cyclotrimerization reactions of alkynes (Reppe-type chemistry) [35], or by the functionalization of appropriately equipped building blocks of type **A**, such as triesters, triamines and trihalides [36] (Figure 1).

In particular, cores featuring nitrogen and/or carbon-based functionalized groups have been successfully used in many materials. An overview is given in Table S1.

Most of the syntheses for aminocarboxy-substituted arenes start with an internal acylation of triaminobenzene. However, the combination of nitrogen and oxidized carbon-based functionalized groups have not been reported in depth. Methyl triaminobenzenetricarboxylate—the only derivative known so far—was used in only one patent, wherein 2,4,6,8,10,12-hexacyano-1,3,5,7,9,11-hexa-azatriphenylene, a derivative of HAT-CN was prepared from trimethyl 2,4,6-triaminobenzene-1,3,5-tricarboxylate (**2a**) via thermal cyclocondensation with urea; halogenation with POCl_3_ in PhNMe_2_; and cyanolysis with KCN in MeCN [37].

**Scheme 1:**
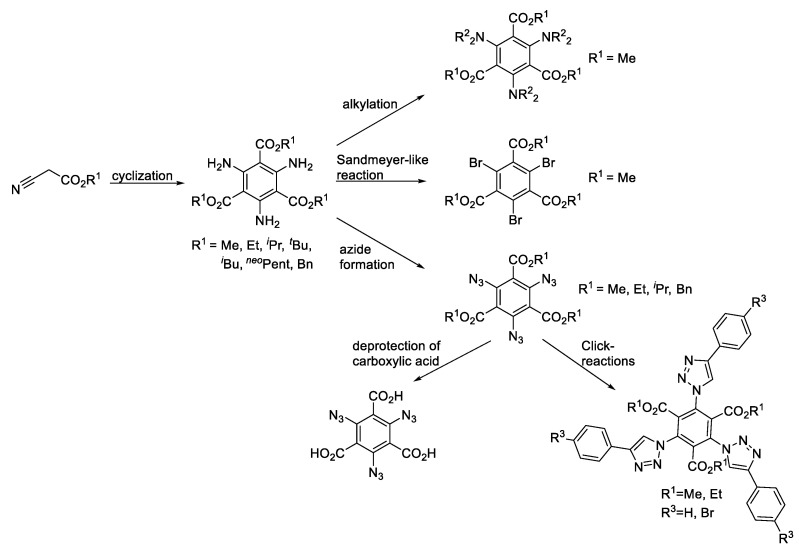
Overview of the herein reported building blocks.

Herein, it is our intention to report the syntheses, structures and reactivities of novel *C*_3_-symmetric fully substituted benzenes with the tandem amino/alkylcarboxylate groups, based on the easily manageable cyclotrimerization of alkyl cyanoacetates. These electronically sophisticated structures of alkyl triamino-benzene-tricarboxylates led to extremely challenging subsequent reactions of the amine group. However, azide formation and a Sandmeyer-like reaction were studied within this report. Due to the low stability of triaminobenzenes, direct *N*-alkylation was rarely reported. Using the stable alkyl triamino-benzene-tricarboxylates allowed us total *N*-alkylation. An overview of the synthesized building blocks is given in Scheme 1.

## 2. Results

### 2.1. Syntheses of C_3_-Symmetric Alkyl Triamino-Benzene-Tricarboxylates

According to the literature-known synthesis for **2a,** starting with the methylcyanoacetate, methyl triamino-benzene-tricarboxylate was synthesized in a moderate yield [38]. Despite extensive experimentation, the yield of the methyl triamino-benzene-tricarboxylate **2a** could not be improved in our hands. However, this atom-economic reaction is scalable and provides the required building block in multi-gram amounts. Besides the methyl triamino-benzene-tricarboxylate **2a**, cyclotrimerization of the alkylcyanoacetates **1b**–**g** led to the derivatives **2b**–**g** in moderate yields (Table 1).

In comparison to 1,3,5-triaminobenzene and several other derivatives, which darken slowly after being left in air, the synthesized alkyl triamino-benzene-tricarboxylates do not show a color change after several months stored in air [39].

### 2.2. Diazotation and Azide Formation

The diazotization and conversion of triaminobenzenes have been reported [40]. Although 1,3,5-triaazidobenzenes are known and reported to be stable, most publications only deal with the theoretical calculations of these molecules [41,42,43,44,45,46,47,48], and there are only around 20 reported examples (including tetra/penta/hexa-azides) [40,43,44,49,50,51,52,53,54,55].

Herein, we report the syntheses of 1,3,5-triazidobenzenes, substituted with ester groups in a 2,4,6-position. The syntheses of triazides **3a**–**g** proceeded from the triamines **2a**–**g** under established conditions through a diazotization reaction. The triazides **3a**–**g** were obtained in yields of 36% to 60% (Table 2). The yields that were achieved for the triazides **3a**–**g** were in the range of other reported triazides that were synthesized from the corresponding amines [40,44]. A procedure in which the diazotization was carried out with tert-butyl nitrite and tosylic acid at 21 °C, followed by the addition of sodium azide, did not result in the formation of the triazide **3a**. Triazides **3a**–**c** and **3g** were stable at normal conditions (daylight included) for several weeks. In the case of the alkyl triamino-benzene-tricarboxylates **2d**, **2e** and **2f**, the mono- and diazides that were also formed during the conversion to the triazide could not be separated from the corresponding triazides via column chromatography. Therefore, these structures are not listed in Table 2.

Cautionary note: azides with a C/N ratio of around 1:1 are potentially explosive [56,57]. 

**Scheme 2:**
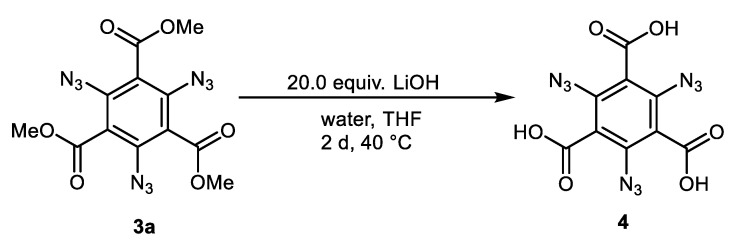
Synthesis of triazidobenzene-tricarboxylic acid **4**.

In the following, the deprotection of the ester was successfully performed to give the triazidobenzene-tricarboxylic acid **4** in a moderate yield of 60% (Scheme 2). These structures might serve as interesting building blocks, e.g., for the generation of trinitrenes [58,59,60] or the synthesis of HKUST1-comparable MOFs.

**Scheme 3:**
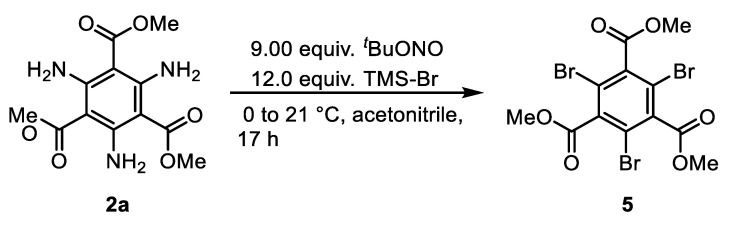
Synthesis of methyl tribromobenzene tricarboxylate **5**.

Due to the ability to form the azide, we thought that reactions based on the diazonium salt, such as Sandmeyer reactions, should be possible. Nevertheless, Sandmeyer-like reactions using *tert*-butyl nitrite or sodium nitrite/hydrochloric acid and potassium iodide failed several times. Many attempts were necessary until conditions were found, which lead to a triple-halogenated compound **5**. This method uses *tert*-butyl nitrite for diazotization and TMS-bromide for halogen transfer. Upon optimization, we find that the addition of the diazotization compound and TMS-bromide must take place alternately, leading to the tribromide **5** in a moderate yield of 34% (Scheme 3). This indicates that diazotization can only be performed at one amine group at a time. Compared to another synthesis route, which has five reaction steps from mesitylene to methyl tribromobenzene tricarboxylate **5**, this route only needs two steps, starting with methylcyanoacetate [8,14].

### 2.3. Click Reactions

Tris-1,2,3-triazoles originating from triazides of type **3** are unknown, except for a single benzotriazole [61] and theoretical investigations [62]. In our hands, the click chemistry that was applied in the case of triazide **3a** in a reaction with phenyl ethyne and *p*-bromophenyl ethyne gave the triazoles **6a-Ph** and **6a-C_6_H_4_Br**, respectively (Table 3). A click reaction with the triazide **3b**, containing an ethyl ester instead of the methyl ester of **3a**, resulted in triazole **6b-Ph**. The yield of **6b-Ph** is noticeably better than that of **6a-Ph**, which can be explained by the better solubility of the ethyl ester **3b** and intermediates on the way to compound **6b-Ph**. Substituted alkynes enable different reactions of these molecules, e.g., network building via dialkynes or coupling reactions of the bromo-substituted tristriazole **6a-C_6_H_4_Br**.

### 2.4. Alkylation

In the past, the direct *N*-alkylation of 1,3,5-triaminobenzene and many derivatives was not possible due to the low stability of the amines. Therefore, benzene-1,3,5-triol or 1,3,5-halogenated structures and secondary amines were commonly used to synthesize alkylated 1,3,5-triamino benzenes [63,64,65]. 

Using the stable methyl triamino-benzene-tricarboxylate **2a** enabled the study of the total *N*-alkylation of 1,3,5- triamino benzenes. To investigate the reactivity of the methyl triamino-benzene-tricarboxylate **2a**, we used different alkyl iodides. The previously unknown hexa-alkyl-triamines **7a**–**c** were successfully synthesized. Purification via column chromatography gave the alkylated structures **7a**–**c** in yields between 38% and 51% (Table 4). The reaction was performed at 100 °C and stirred for 16 h in case of **7a** and **2d**, in case of **7b** and **7c**. TLC of the crude reaction mixtures showed less alkylated fractions. Nevertheless, longer reaction times could not improve the yields.

### 2.5. Molecular Structures

**Figure 2:**
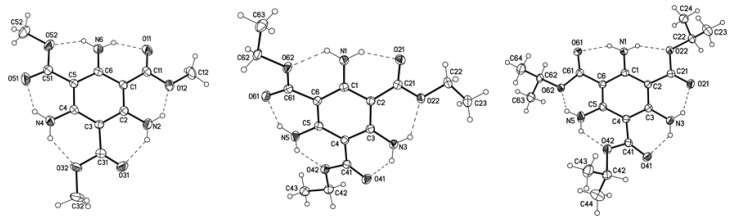
Molecular structures of **2a**, **2b** and **2c**, intramolecular hydrogen bonds drawn at dashed lines, displacement parameters are drawn at 50% probability level (for details, see supporting information and cif-file).

The structures of the alkyl triamino-benzene-tricarboxylates **2a**–**c** were additionally confirmed by X-ray crystallography. The molecular structures show the possible formation of hydrogen bonds between the amino and ester groups, leading to the expected planar structure of **2**. While **2a** showed a planar propeller-like arrangement, the sterically more demanding ester groups of **2b** and **2c** twisted the functional groups marginally out of the plane (Figure 2).

**Figure 3:**
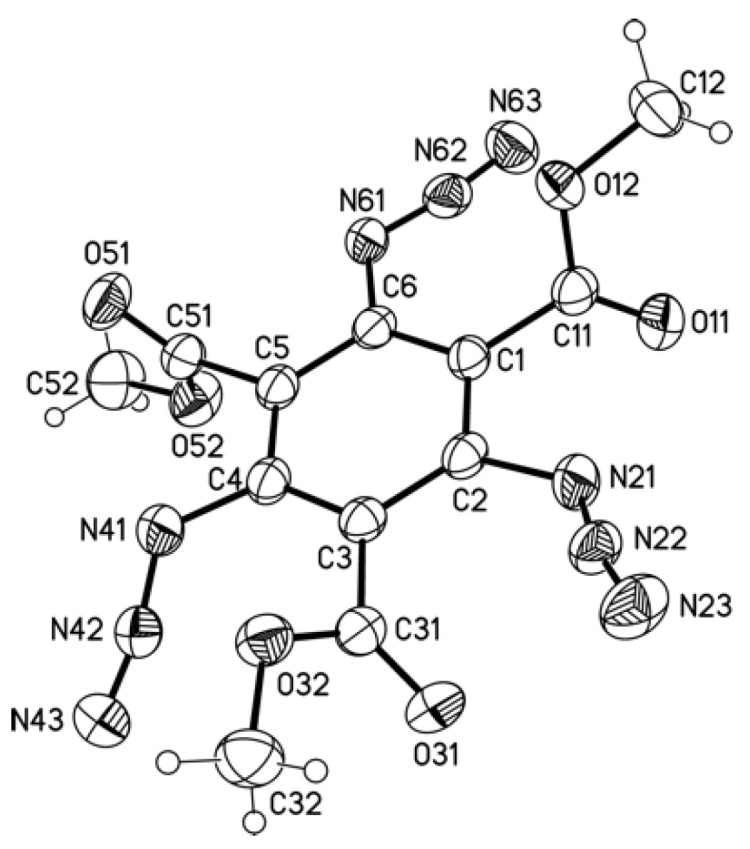
Molecular structure of **3a**, displacement parameters are drawn at 30% probability level (for details, see supporting information and cif-file).

Beside the triamines **2a**–**c**, the structure of **3a** was also confirmed by the molecular structure, but due to the poor crystal quality we will not discuss this further (Figure 3).

## 3. Materials and Methods

### 3.1. General Procedure for Cyclotrimerizations

A pressure tube was charged with Cu(OAc)_2_*H_2_O (0.10 equiv.) and 1,4-dioxane. Alkyl cyanoacetate (1.00 equiv.) was added, and the mixture was bubbled with argon for 5 min. The mixture was heated to 130 °C for 72 h. After cooling to room temperature, the solid was filtered off, and the solvent was removed under reduced pressure. The product was purified by column chromatography (cyclohexane/ethyl acetate).

### 3.2. General Procedure for the Syntheses of Azides

Trialkyl 2,4,6-triaminobenzene-1,3,5-tricarboxylate (1.00 equiv.) was solved in THF and cooled to 0 °C. tert-Butyl nitrite (9.00 equiv.) was added dropwise. The mixture was stirred for 30 minutes, followed by the addition azido(trimethyl)silane (slow, 6.00 equiv.). The mixture was stirred for 72 h. The solvent was (carefully) removed under reduced pressure, and the residue was purified by column chromatography (cyclohexane/ethyl acetate).

### 3.3. Typical Procedure for the Click Reactions

**3a** (100 mg, 240 μmol, 1.00 equiv) and ethynylbenzene (85.7 mg, 839 μmol, 3.50 equiv.) were solved under argon in degassed DMSO (2.50 mL). Copper sulfate pentahydrate (8.97 mg, 35.9 μmol, 0.150 equiv) and sodium ascorbate (14.2 mg, 71.9 μmol, 0.300 equiv) were solved in degassed water (500 μL) and degassed DMSO (2.50 mL). The mixture was added dropwise to the ethynylbenzene-solution. The mixture was stirred at 50 °C for 3 d. After cooling to 25 °C, ethyl acetate (20 mL) and water (20 mL) were added, and the phases were separated. The organic layer was washed with brine (20 mL) and then dried over sodium sulfate. The solvent was removed under reduced pressure, and the residue was purified via flash chromatography (cyclohexane/ethyl acetate 20:1 to 4:1) to give the desired product **6a-Ph** as a light-yellow solid.

### 3.4. Crystal Structure Determination

The single-crystal X-ray diffraction studies were carried out on a Bruker D8 Venture diffractometer with a PhotonII detector at 123(2) K; 173(2) K; or 298(2) K using Cu-Kα radiation (*λ* = 1.54178 Å). Dual space methods (SHELXT) [66] were used for the structure solution, and refinement was carried out using SHELXL (full-matrix least-squares on *F*^2^) [67]. Hydrogen atoms were localized by difference electron density determination and refined using a riding model (H(N, O) free). Semi-empirical absorption corrections were applied. CCDC 2,102,766 (**2a**); 2,102,767 (**2b**); and 2,102,768 (**2c**) contain the supplementary crystallographic data for this paper. These data can be obtained free of charge from The Cambridge Crystallographic Data Centre via www.ccdc.cam.ac.uk/data_request/cif (accessed on 12 August 2021). Due to the bad quality of the data of **3a** (completeness approx. 82%), the data were not deposited with The Cambridge Crystallographic Data Centre).

### 3.5. NMR Measurements

The NMR spectra were recorded at 25 °C on an Bruker Avance 400 NMR instrument. More details on the NMR measurements can be found in the Supplementary Information.

## 4. Conclusions

Different *C*_3_-symmetric building blocks based on alkyl triamino-benzene-tricarboxylates have been reported in this manuscript. Despite only moderate yields, the simplicity of the syntheses allowed gram amounts of the alkyl triamino-benzene-tricarboxylates. Starting from the remarkably stable alkyl-triamino-benzene-tricarboxylates, we investigated azide formation and Sandmeyer-like reactions, as well as chemo-selective *N*-hexa-alkylation. More interestingly, click reactions were possible with the synthesized triazides, allowing further studies on the formation of porous organic polymers (POP). With the triazidobenzene-tricarboxylic acid, we have presented a building block that can be used, for example, in a similar way to trimesic acid in the synthesis of MOFs.

## Figures and Tables

**Table 1:** Scope of the various alkyl triamino-benzene-tricarboxylates **2a**–**g**.

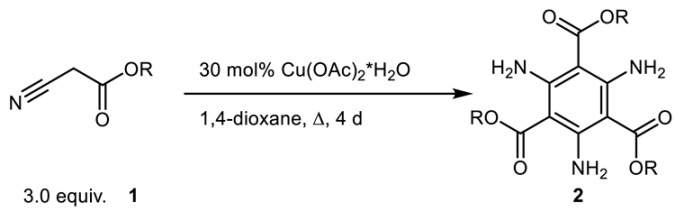				
Starting Material	R	Product	Temp. [°C]	Yields [%]
**1a**	Me	**2a**	130	35 ^a^
**1b**	Et	**2b**	130	28
**1c**	*^i^* Pr	**2c**	130	18
**1d**	*^t^* Bu	**2d**	100	15
**1e**	*^i^* Bu	**2e**	100	12
**1f**	*^n^* Pent	**2f**	100	22
**1g**	Bn	**2g**	130	23

**Table 2:** Syntheses of triazides **3a**–**c**, **g**.

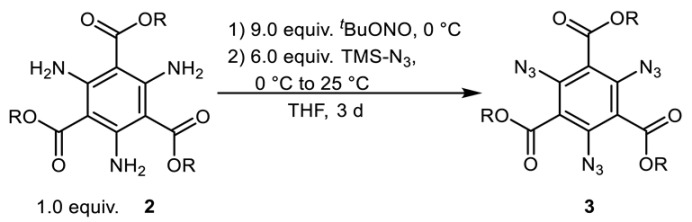			
Starting Material	R	Product	Yield [%]
**2a**	Me	**3a**	60
**2b**	Et	**3b**	47
**2c**	*^i^* Pr	**3c**	50
**2g**	Bn	**3g**	36

**Table 3:** Syntheses of tris-1,2,3-triazoles **6**.

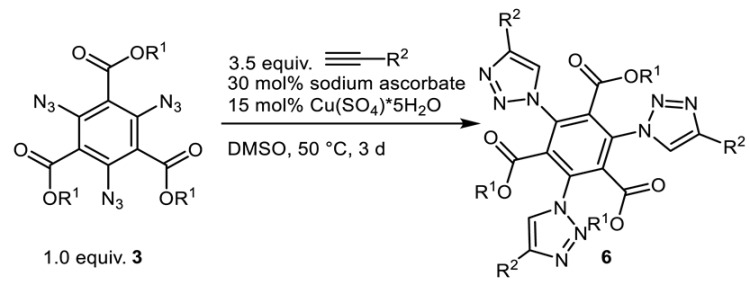				
Starting Material	R^1^	Product	R^2^	Yield
**3a**	Me	**6a-Ph**	Ph	19
**3a**	Me	**6a-C_6_H_4_Br**	C_6_H_4_Br	18
**3b**	Et	**6b-Ph**	Ph	27

**Table 4:** Chemo-selective hexa-N-alkylation of methyl triamino-benzene-tricarboxylate **2a**.

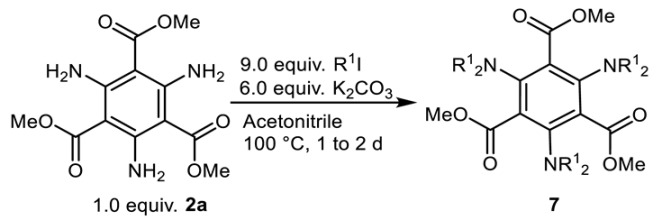		
Product	R	Yield [%]
**7a**	Me	48
**7b**	Et	38
**7c**	Bn	51

## Data Availability

The procedures, as well as the analytical data, are available online in the Chemotion Repository (https://www.chemotion-repository.net/welcome (accessed on 28 June 2022)). Exact links are given in the Supplementary Information.

## Supplementary Materials

The following are available online at https://www.mdpi.com/article/10.3390/molecules27144369/s1: synthetic procedure, crystallographic, and NMR data.

## Sample Availability

Not available.
