# Supplementary material for: Functionalized C3-Symmetric Building Blocks—The Chemistry of Triaminotrimesic Acid
Source: Molecules. 2022 Jul 7;27(14):4369. doi: 10.3390/molecules27144369 (PMC9322044; doi:10.3390/molecules27144369)
Supplement: Supplementary file 1 [file molecules-27-04369-s001.zip › molecules-1771940-supplementary.pdf]

Functionalized C_3-Symmetric Building Blocks—The Chemistry of Triaminotrimesic Acid

Lisa Schmidt [1], Danny Wagner [1], Martin Nieger [2] and Stefan Bräse [1,3,*]

[1] Institute of Organic Chemistry, Karlsruhe Institute of Technology (KIT), Fritz-Haber-Weg 6,
76131 Karlsruhe, Germany; lisa.schmidt@kit.edu (L.S.); danny.wagner2@kit.edu (D.W.)
[2] Department of Chemistry, University of Helsinki, P.O. Box 55 (A. I. Virtasen aukio 1), FIN-00014 Helsinki, Finland; martin.nieger@helsinki.fi
[3] Institute of Biological and Chemical Systems—FMS, Karlsruhe Institute of Technology (KIT),
Hermann-Von-Helmholtz-Platz 1, 76344 Leopoldshafen, Germany
* Correspondence: braese@kit.edu; Tel.: +49-721-608-42903; Fax: +49-721-608-48581

Contents

1. Overview of some synthetically known C3-symmetric building blocks 2
2. General remarks 3
3. Syntheses and analytical data 3
   3.1 Syntheses of triaminobenzene-1,3,5-alkyltricarboxylates **2a-g** 4
   3.2 Syntheses of 2,4,6-triazidobenzene-1,3,5-alkyltricarboxylates **3a-c, 3g,** 2,4,6-Triazidobenzene-1,3,5-tricarboxylic acid (**4**) and Trimethyl-2,4,6-tribromobenzene-1,3,5-tricarboxylate (**5**) 9
   3.3 Click reactions of azides **3a and 3b** 13
   3.4 Alkylation of amine **2a** 16
4. Crystallographic Information 19
5. ^{1}H- and ^{13}C-NMR spectra 23
6. References 42

1. Overview of some synthetically known C3-symmetric building blocks

Table S1. Overview of some synthetically known C3-symmetric building blocks A [72]—highlighted: combinations investigated in this report.

X/Y	OR	Hal	NH_2	NR_2[1]	NO_2	N_3	CO_2H	CO_2R	CN	$CONR_2$	CHO
OR	-	Br [2]	unknown	[3]	[4]	unknown	[5]	[6]	[7–9]	[5,6,10–13]	[6,11,14]
Hal	Br [2]	I/Cl, I/Br[15]	Br,Cl:[16–22]	[23]	Br,Cl: [24]	[17]	I: [25]; Br: [26]; F: [27,28]	Br: [29] [30]	[31]	many examples[32] [30]	Br [33]
NH_2	unknown	Br,Cl:[16–22]	-		TATB [3,34]	unknown	unknown	[35,36]	[18,35,37–39]		unknown
NR_2	[3]	[23]		-	[4],[40]		unknown	unknown	[38,41–45]	unknown	unknown
NO_2	[4]	Br,Cl: [24]	TATB [3,34]	[40]	-	[46]	[47]	[47–49]	unknown	unknown	unknown
N_3	unknown	[17]	unknown		[46]	-	unknown	unknown			
CO_2H	[5]	I: [25]; Br: [26]; F: [28]	unknown	unknown	[47]	unknown	-				
CO_2R	[6]	Br: [29] [30]	[35,36]	unknown	[47,48] [49]	unknown		-			
CN	[7–9]	[31]	[18,35,37–39]	[38,41–45]	unknown	[39,50–52]			-		
$CONR_2$	[5,6,10–13]	many examples [32] [30]	unknown	unknown	unknown	unknown				-	
CHO	[6,11,14]	Br [31,33]	unknown	unknown	unknown	unknown					-
Alkyl	many examples [53],[54]	[55,56]	[57,58]	[59]	[60]	unknown					
Alkenyl											
Aryl				[61]							
Alkynyl	[62]	[63]			[60]	unknown		[29]	[64]	[29] [65,66] [67]	[68–71]

2. General remarks

The NMR (Nuclear Magnetic Resonance) spectra were recorded on a BRUKER Avance 400 (400 MHz (^{1}H), 101 MHz (^{13}C)) spectrometer. Chemical shifts (δ) are expressed in parts per million (ppm). All spectra are referenced to the signals of the residual protons of the solvents chloroform-d1 (7.26 ppm (1H), 77.2 ppm (13C)); or dimethylsulfoxide-d6 (2.50 ppm (1H), 39.5 ppm (13C)) as an internal standard. The spectra were analyzed according to the first order. Coupling constants (J) are given in Hertz (Hz). The different signals are described as follows: br = broad signal; s = singlet; d = doublet; t = triplet; q = quartet; quin = quintet; m = multiplet.

FAB (Fast Atom Bombardment) and EI (Electron Impact) mass spectra, as well as HRMS (High-Resolution Mass Spectra), were obtained using a Finnigan MAT 95 mass spectrometer. The indication of the molecular fragments was carried out as the ratio of mass to charge m/z; for EI mass, the intensity of the signals was expressed in percent relative to the intensity of the base signal (100%). For High-Resolution Mass Spectrometry (HR-MS), the following abbreviations were used: calcd = calculated; mass found = mass found in the analysis.

An Attenuated Total Reflection Fourier Transform Infrared Spectroscopy (ATR-FTIR) was conducted on a Bruker Alpha T or Tensor-27 at ambient temperature with the OPUS software. The position of the absorption band was given in wavenumbers ν in cm-1. The shapes and intensities of the bands were characterized as follows: vs = very strong 0-10% T (Transmittance); s = strong 11–40% T; m = medium 41–70% T; w = weak 71- 90% T; vw = very weak, 91–100% T.

TLC (Thin Layer Chromatography) reaction monitoring was carried out using silica gel coated aluminum plates (Merck, silica gel 60, F254), which were analyzed under UV light at 254 nm. Flash-chromatography was conducted on silica gel. The solvent mixtures for TLC and flash-chromatography are understood as volume/volume, with each volume measured separately.

The solvents, reagents and chemicals were purchased from Sigma-Aldrich, Alfa Aesar, ABCR, Chempur, Thermo Fisher Scientific, TCI, and VWR, and used without further purification, unless stated otherwise.

Please note that full experimental details and the original analytical data files can be accessed, examined and downloaded from the repository Chemotion (https://www.chemotion-repository.net/) via the DOIs that are given at the end of the reaction procedure. For this supporting information, the reaction descriptions and analytical data were generated automatically via the repository Chemotion and adjusted and enriched with additional information.

3. Syntheses and analytical data

General procedure 1: Aromatic-cyclotrimerizations

A pressure tube was charged with $Cu(OAc)_2*H_2O$ (0.10 equiv.) and 1,4 dioxane. Cyanoacetate (1.00 equiv.) was added and the mixture was bubbled with argon for 5 min. The mixture was heated to 130 °C for 72 h. After cooling to room temperature, the mixture was filtered off, and the solvent was removed under reduced pressure. The crude product was purified by column chromatography (cyclohexane/ethyl acetate).

General procedure 2: Azide-synthesis

Trimethyl 2,4,6-triaminobenzene-1,3,5-tricarboxylate (1.00 equiv.) was solved in THF and cooled to 0 °C. Tert-butyl nitrite (9.00 equiv.) was added dropwise. The mixture was stirred for 30 minutes, followed by the addition of azido(trimethyl)silane (slow, 6.00 equiv.). The mixture was stirred for 72 h. The solvent was (carefully) removed under reduced pressure and the residue was purified by column chromatography (cyclohexane/ethyl acetate).

3.1. Syntheses of triaminobenzene-1,3,5-alkyltricarboxylates 2a-g

Trimethyl-2,4,6-triaminobenzene-1,3,5-tricarboxylate (2a)

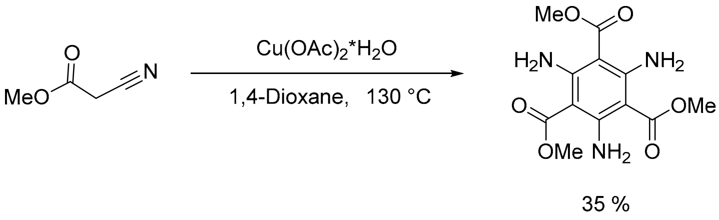

According to GP1, the reaction was carried out with methyl 2-cyanoacetate (1.50 g, 15.1 mmol, 1.00 equiv.) and $Cu(OAc)_2*H_2O$ (302 mg, 1.51 mmol, 0.10 equiv.) in 1,4-dioxane (30 mL). Column chromatography (cyclohexane/ethyl acetate 10:1 to 6:1). The product **2a** was obtained as a colorless solid (519 mg, 1.75 mmol) in a yield of 35%.

Multi-gram-scale: performed in a 1000 mL Duran bottle (pressure plus) with an oil bath for heating. $Cu(OAc)_2*H_2O$ (5.04 g, 25.2 mmol, 0.10 equiv.), 500 mL 1,4-dioxane, methyl 2-cyanoacetate (25.0 g, 252 mmol, 1.00 equiv.). GP 1. The product was obtained as a colorless solid (8.66 g, 29.1 mmol) in a yield of 35%.

R_f = 0.32 (cyclohexane/ethyl acetate 4:1). ^{1}H NMR (400 MHz, $CDCl_3$): δ [ppm] = 8.78 (s, 6H, N*H*$_2$), 3.87 (s, 9H, C*H*$_3$). ^{13}C NMR (101 MHz, $CDCl_3$): δ [ppm] = 169.2, 159.4, 86.7, 51.24. MS (FAB, 3-NBA), m/z (%): 298 (57) $[M+H]^+$, 297 (77) $[M]^+$, 267 (15), 266 (100), 234 (23), 154 (17), 147 (15), 136 (23), 119 (16), 109 (27), 107 (22), 105 (22), 97 (26), 95 (49), 93 (27), 91 (44). HRMS–FAB *(m/z)*: $[M]^+$ calcd for $C_{12}H_{15}O_6N_3$: 297.0955; found 297.0954. IR (ATR, ṽ) = 3479 (m), 3442 (m), 3282 (m), 3247 (m), 2993 (w), 2948 (w), 2904 (w), 1754 (vw), 1677 (w), 1655 (m), 1596 (m), 1560 (vs), 1509 (vs), 1466 (s), 1426 (vs), 1295 (w), 1215 (vs), 1163 (vs), 1064 (vs), 976 (s), 850 (m), 809 (vs), 789 (s), 722 (s), 671 (s), 602 (m), 596 (m), 581 (w), 528 (s), 469 (vs), 438 (vs), 392 (vs) cm^{-1}.

The structure **2a** could be confirmed by single-crystal X-ray diffraction (see 3 Crystallographic Information).

The analytical data are in accordance with the literature [35].

Additional information on the chemical synthesis is available via the Chemotion repository: https://dx.doi.org/10.14272/reaction/SA-FUHFF-UHFFFADPSC-KLJBLDCULB-UHFFFADPSC-NUHFF-NUHFF-NUHFF-ZZZ

Additional information on the analysis of the target compound is available via the Chemotion repository: https://dx.doi.org/10.14272/KLJBLDCULBNQFL-UHFFFAOYSA-N.1

Triethyl-2,4,6-triaminobenzene-1,3,5-tricarboxylate (2b)

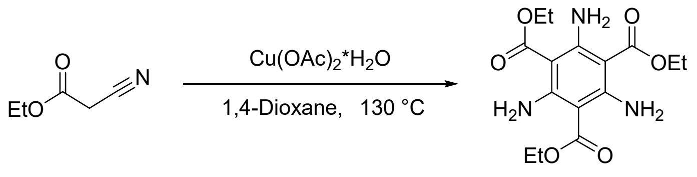

28 %

According to GP1, the reaction was carried out with ethyl 2-cyanoacetate (1.80 g, 15.9 mmol, 1.00 equiv.) and $Cu(OAc)_2*H_2O$ (318 mg, 1.59 mmol, 0.10 equiv.) in 1,4-dioxane (32 mL). Column chromatography (cyclohexane/ethyl acetate 10:1). The product **2b** was obtained as a colorless solid (504 mg, 1.498 mmol) in a yield of 28%.

R_f = 0.31 (cyclohexane/ethyl acetate 10:1). ^{1}H NMR (400 MHz, $CDCl_3$): δ [ppm] = 8.81(s, 6H, N*H*$_2$), 4.36 (q, $^3J_{H,H}$ = 7.0 Hz, 6H, C*H*$_2$), 1.39 (t, $^3J_{H,H}$ = 7.1 Hz, 9H, C*H*$_3$). ^{13}C NMR (101 MHz, $CDCl_3$): δ [ppm] = 168.9, 159.4, 86.8, 60.6, 14.6. MS (FAB, 3-NBA), m/z (%): 532 (10), 531 (28), 442 (11), 441 (33), 418 (26) $[M+H]^+$, 417 (30) $[M]^+$, 403 (17), 391 (38), 390 (86), 372 (63), 344 (23), 260 (15), 232 (19), 216 (17), 206 (17), 191 (15), 190 (16), 189 (21), 188 (52), 186 (25), 182 (15), 161 (20), 160 (30), 159 (16), 155 (25), 154 (83), 152 (17), 150 (16), 149 (15), 147 (47), 139 (20), 138 (35), 137 (100), 136 (95), 131 (18), 129 (16), 124 (20), 123 (16), 121 (15), 120 (16), 119 (19), 117 (15), 115 (16), 111 (15), 109 (28), 107 (28), 105 (20), 97 (46), 95 (29), 93 (28), 91 (25). HRMS–FAB *(m/z)*: $[M]^+$ calcd for $C_{15}H_{15}O_6N_9$: 417.1140; found 417.1141. IR (ATR, ṽ) = 2990 (w), 2965 (w), 2942 (w), 2113 (vs), 1731 (vs), 1713 (vs), 1561 (vs), 1462 (w), 1443 (w), 1422 (m), 1384 (w), 1377 (m), 1363 (s), 1307 (s), 1281 (vs), 1256 (vs), 1211 (vs), 1176 (vs), 1160 (vs), 1133 (s), 1115 (m), 1092 (s), 1017 (vs), 972 (s), 911 (m), 892 (m), 860 (s), 834 (m), 803 (w), 782 (m), 766 (w), 747 (w), 734 (m), 715 (m), 652 (w), 612 (s), 584 (m), 560 (w), 523 (s), 487 (w), 458 (w), 431 (w), 425 (w), 418 (w), 405 (w), 384 (m) cm^{-1}.

The structure **2b** could be confirmed by single-crystal X-ray diffraction (see 5 Crystallographic Information).

Additional information on the chemical synthesis is available via the Chemotion repository: https://dx.doi.org/10.14272/reaction/SA-FUHFF-UHFFFADPSC-HCVSNXJKNA-UHFFFADPSC-NUHFF-NUHFF-NUHFF-ZZZ

Additional information on the analysis of the target compound is available via the Chemotion repository: https://dx.doi.org/10.14272/HCVSNXJKNAAQBQ-UHFFFAOYSA-N.1

Triisopropyl-2,4,6-triaminobenzene-1,3,5-tricarboxylate (2c)

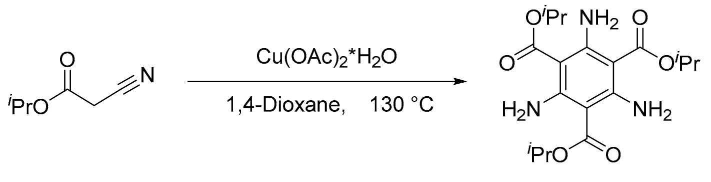

30 %

According to GP1, the reaction was carried out with isopropyl 2-cyanoacetate (2.00 g, 1.97 mL, 15.7 mmol, 1.00 equiv.) and $Cu(OAc)_2*H_2O$ (314 mg, 1.57 mmol, 0.300 equiv.) in 1,4-dioxane (31.5 mL). The mixture was heated to 100 °C for 4 d. Column chromatography (cyclohexane/ethyl acetate 10:1). The product **2c** was obtained as a colorless solid (504 mg, 1.498 mmol) in a yield of 30%.

R_f (cyclohexane/ethyl acetate 10:1) = 0.28. ^{1}H-NMR (400 MHz, $CDCl_3$): δ [ppm] = 8.78 (s, 6H, NH_2), 5.28 (hept, $^3J_{H,H}$ = 6.3 Hz, 3H, CH), 1.38 (d, $^3J_{H,H}$ = 6.2 Hz, 18H, CH_3). ^{13}C NMR (101 MHz, $CDCl_3$): δ [ppm] = 168.6 159.3, 87.1, 68.3, 22.4. MS (FAB, 3-NBA), m/z (%): 383 (15), 382 (69), 381 (100) [M]+, 322 (71), 238 (22), 220 (18), 193 (20). HRMS–FAB *(m/z)*: [M + H]$^+$ calcd for $C_{18}H_{27}O_6N_3$: 381.1900. found 381.1901. IR (ATR, ṽ) = 3500 (m), 3458 (m), 3397 (m), 3364 (w), 3251 (w), 2982 (w), 2931 (w), 2873 (vw), 1730 (vw), 1672 (s), 1659 (s), 1632 (w), 1562 (vs), 1519 (vs), 1452 (s), 1432 (s), 1385 (m), 1370 (s), 1346 (m), 1217 (vs), 1174 (vs), 1145 (vs), 1105 (vs), 1057 (vs), 924 (vs), 833 (m), 809 (vs), 748 (w), 725 (m), 705 (m), 681 (m), 609 (vs), 552 (m), 507 (vs), 460 (vs), 446 (vs), 435 (vs), 380 (vs) cm^{-1}.

The structure **2c** could be confirmed by single-crystal X-ray diffraction (see 5 Crystallographic Information).

Additional information on the chemical synthesis is available via the Chemotion repository: https://dx.doi.org/10.14272/reaction/SA-FUHFF-UHFFFADPSC-RJJPADYKFC-UHFFFADPSC-NUHFF-NUHFF-NUHFF-ZZZ

Additional information on the analysis of the target compound is available via the Chemotion repository: https://dx.doi.org/10.14272/RJJPADYKFCVKLL-UHFFFAOYSA-N.1

Tris(tert-Butyl)-2,4,6-triaminobenzene-1,3,5-tricarboxylate (2d)

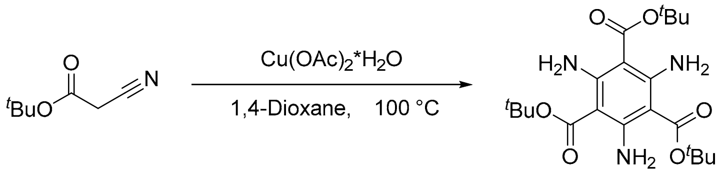

14 %

According to GP1, the reaction was carried out with tert-butyl 2-cyanoacetate (5.00 g, 35.4 mmol, 1.00 equiv.) and $Cu(OAc)_2$*H_2O (707 mg, 3.54 mmol, 0.100 equiv.) in 1,4-dioxane (32 mL). The mixture was heated to 100 °C for 4 d. Column chromatography (cyclohexane/ethyl acetate 10:1 to 4:1). The product **2d** was obtained as a colorless solid (683 mg, 1.61 mmol) in a yield of 14%.

R_f = 0.76 (cyclohexane/ethyl acetate 4:1). ^{1}H NMR (400 MHz, $CDCl_3$): δ [ppm] = 8.68 (s, 6H, NH_2), 1.59 (s, 27H, CH_3). ^{13}C NMR (101 MHz, $CDCl_3$): δ [ppm]= 168.7, 159.0, 88.0, 82.1, 28.9. MS (FAB, 3-NBA), m/z (%): 425 (12), 424 (51) [M+H]$^+$, 423 (42) [M]$^+$, 350 (15), 256 (40), 255 (100), 238 (79), 220 (18), 194 (20), 193 (29), 154 (29), 137 (18), 136 (23). HRMS–FAB *(m/z)*: [M + H]$^+$ calcd for $C_{21}H_{34}O_6N_3$: 424.2442; found 242.2443. IR (ATR, ṽ) = 3458 (s), 3272 (w), 3237 (w), 3221 (w), 3200 (w), 3155 (vw), 3007 (w), 2976 (w), 2929 (w), 2884 (vw), 1638 (s), 1577 (vs), 1526 (m), 1475 (m), 1452 (m), 1426 (w), 1387 (m), 1363 (s), 1302 (vw), 1235 (vs), 1159 (s), 1132 (vs), 1071 (vs), 924 (m), 902 (m), 861 (s), 800 (vs), 771 (m), 728 (w), 717 (w), 667 (w), 622 (vw), 609 (vw), 595 (vw), 578 (vw), 568 (vw), 554 (vw), 516 (s), 465 (vs), 385 (w) cm^{-1}.

Additional information on the chemical synthesis is available via the Chemotion repository: https://dx.doi.org/10.14272/reaction/SA-FUHFF-UHFFFADPSC-JYBSZBKAER-UHFFFADPSC-NUHFF-NUHFF-NUHFF-ZZZ

Additional information on the analysis of the target compound is available via the Chemotion repository: https://dx.doi.org/10.14272/JYBSZBKAERXMLG-UHFFFAOYSA-N.1

Tri(iso-Butyl)-2,4,6-triaminobenzene-1,3,5-tricarboxylate (2e)

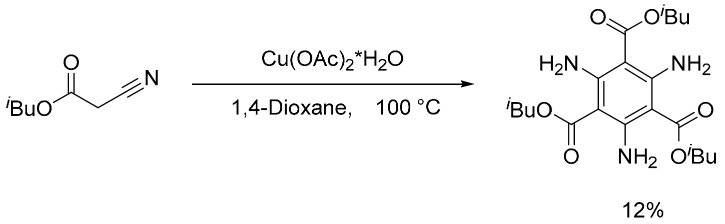

According to GP1, the reaction was carried out with iso-butyl 2-cyanoacetate (4.50 g, 31.9 mmol, 1.00 equiv.) and $Cu(OAc)_2*H_2O$ (636 mg, 3.19 mmol, 0.100 equiv.) in 1,4-dioxane (30 mL). The mixture was stirred for 3 d at 100 °C. Column chromatography (cyclohexane/ethyl acetate 10:1). The product **2e** was obtained as a colorless solid (527 mg, 1.24 mmol) in a yield of 12%.

R_f (cyclohexane/ethyl acetate 4:1) = 0.74. ^{1}H NMR (400 MHz, $CDCl_3$): δ [ppm] = 8.87 (s, 6H, NH_2), 4.13 (d, J = 6.8 Hz, 6H, CH_2), 2.17–2.04 (m, 3H, C*H*), 1.03 (d, J = 6.8 Hz, 18H, CH_3). ^{13}C NMR (101 MHz, $CDCl_3$): δ = 169.1, 159.5, 86.9, 70.9, 27.7, 19.6. MS (FAB, 3-NBA), m/z (%): 425 (16), 424 (71) $[M+H]^+$, 423 (100) $[M]^+$, 351 (18), 350 (80), 220 (18). HRMS–FAB *(m/z)*: $[M + H]^+$ calcd for $C_{21}H_{33}O_6N_3$: 423.2364; found 423.2362. IR (ATR, ṽ) = 3500 (m), 3458 (m), 3394 (m), 3276 (w), 2966 (m), 2958 (m), 2928 (w), 2912 (w), 2871 (w), 1667 (s), 1629 (w), 1555 (vs), 1516 (vs), 1458 (s), 1375 (s), 1366 (s), 1303 (w), 1249 (m), 1221 (vs), 1167 (vs), 1102 (m), 1060 (vs), 997 (vs), 982 (vs), 963 (s), 949 (s), 926 (m), 912 (m), 895 (m), 856 (m), 809 (vs), 785 (m), 724 (s), 714 (m), 673 (s), 660 (s), 642 (vs), 613 (m), 586 (m), 534 (m), 487 (vs), 467 (vs), 425 (vs), 375 (vs) cm^{-1}.

Additional information on the chemical synthesis is available via the Chemotion repository: https://dx.doi.org/10.14272/reaction/SA-FUHFF-UHFFFADPSC-MZVWXNMVEP-UHFFFADPSC-NUHFF-NUHFF-NUHFF-ZZZ

Additional information on the analysis of the target compound is available via the Chemotion repository: https://dx.doi.org/10.14272/MZVWXNMVEPSDOD-UHFFFAOYSA-N.1

Tri(neo-Pentyl)-2,4,6-triaminobenzene-1,3,5-tricarboxylate (2f)

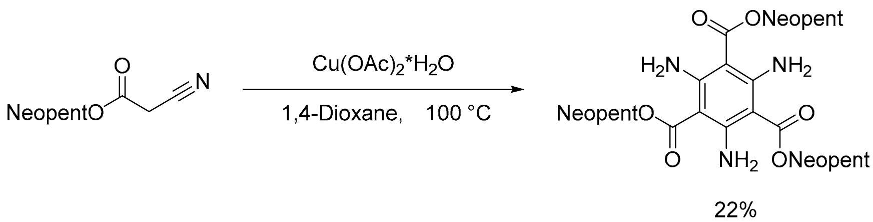

According to GP1, the reaction was carried out with 2,2-dimethylpropyl 2-cyanoacetate (5.00 g, 32.2 mmol, 1.00 equiv.) and $Cu(OAc)_2*H_2O$ (643 mg, 3.22 mmol, 0.100 equiv.) in 1,4-dioxane (30 mL). The mixture was stirred for 3d at 100 °C. Column chromatography (cyclohexane/ethyl acetate 10:1). The product **2f** was obtained as a colorless solid (1.09 g, 2.34 mmol) in a yield of 22%

R_f (cyclohexane/ethyl acetate 4:1) = 0.76. ^{1}H NMR (400 MHz, $CDCl_3$): δ [ppm] = 8.87 (s, 6H, N*H*$_2$), 4.04 (s, 6H, C*H*$_2$), 1.02 (s, 27H, C*H*$_3$). ^{13}C NMR (101 MHz, $CDCl_3$): δ [ppm] = 169.2, 159.5, 86.9, 74.3, 31.5, 27.0. MS (FAB, 3-NBA), m/z (%): 467 (14), 466 (55) $[M+H]^+$, 465 (100) $[M]^+$, 378 (63), 220 (15). HRMS–FAB *(m/z)*: $[M]^+$ calcd for $C_{24}H_{39}O_6N_3$: 465.2833; found 465.2835. IR (ATR, ṽ) = 3463 (s), 3306 (w), 3278 (w), 3242 (w), 2959 (w), 2904 (w), 2898 (w), 2887 (w), 2868 (w), 1655 (s), 1570 (vs), 1523 (vs), 1475 (m), 1431 (s), 1397 (w), 1366 (m), 1269 (w), 1221 (vs), 1071 (vs), 1037 (m), 977 (vs), 949 (m), 938 (w), 928 (m), 916 (w), 888 (w), 810 (vs), 758 (w), 730 (w), 671 (s), 527 (w), 482 (vs), 449 (vs), 431 (vs), 402 (vs) cm^{-1}.

Additional information on the chemical synthesis is available via the Chemotion repository: https://dx.doi.org/10.14272/reaction/SA-FUHFF-UHFFFADPSC-GSTZJTYYYM-UHFFFADPSC-NUHFF-NUHFF-NUHFF-ZZZ

Additional information on the analysis of the target compound is available via the Chemotion repository: https://dx.doi.org/10.14272/GSTZJTYYYMCAPJ-UHFFFAOYSA-N.1

Tribenzyl-2,4,6-triaminobenzene-1,3,5-tricarboxylate (2g)

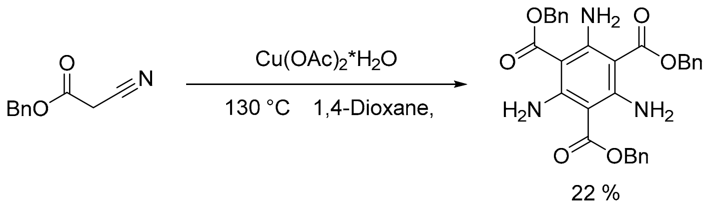

According to GP1, the reaction was carried out with benzyl 2-cyanoacetate (5.00 g, 28.54 mmol, 1.00 equiv.) and $Cu(OAc)_2*H_2O$ (570 mg, 2.85 mmol, 0.100 equiv.) in 1,4-dioxane (35 mL). The mixture was stirred for 64 h at 130 °C. Column chromatography (cyclohexane/ethyl acetate 4:1). The product **2g** was obtained as a colorless solid (1.09 g, 2.07 mmol) in a yield of 22%.

R_f (cyclohexane/ethyl acetate 4:1) = 0.39. ^{1}H NMR (400 MHz, $CDCl_3$): δ [ppm] = 8.80 (s, 6H, N*H*$_2$), 7.44 – 7.29 (m, 15H, Ar*H*), 5.32 (s, 6H, C*H*$_2$). ^{13}C NMR (101 MHz, $CDCl_3$): δ [ppm] = 168.6, 159.6, 135.9, 128.9, 128.5, 128.3, 86.6, 66.4. MS (FAB, 3-NBA), m/z (%): 527 (18), 526 (62) $[M+H]^+$, 525 (52) $[M]^+$, 419 (18), 418 (63), 155 (16), 154 (53), 138 (20), 137 (35), 136 (45), 91 (100). HRMS–FAB *(m/z)*: $[M + H]^+$ calcd for $C_{30}H_{27}O_6N_3$: 525.1894; found 525.1892. IR (ATR, ṽ) = 3495 (m), 3455 (m), 3384 (m), 3305 (w), 3282 (w), 3254 (w), 3089 (w), 3058 (w), 3033 (w), 3004 (w), 2945 (w), 2888 (w), 1676 (m), 1650 (m), 1629 (w), 1560 (vs), 1514 (vs), 1496 (vs), 1451 (s), 1434 (vs), 1370 (m), 1364 (m), 1310 (w), 1279 (w), 1231 (vs), 1215 (vs), 1157 (vs), 1057 (vs), 1026 (vs), 1000 (s), 967 (s), 916 (s), 899 (s), 894 (s), 853 (w), 836 (m), 807 (vs), 785 (m), 755 (s), 725 (vs), 696 (vs), 677 (s), 643 (vs), 613 (s), 588 (vs), 565 (vs), 523 (vs), 497 (vs), 463 (vs), 453 (vs), 416 (vs), 395 (s), 380 (s) cm^{-1}.

Additional information on the chemical synthesis is available via the Chemotion repository: https://dx.doi.org/10.14272/reaction/SA-FUHFF-UHFFFADPSC-UOEJOXKVDY-UHFFFADPSC-NUHFF-NUHFF-NUHFF-ZZZ

Additional information on the analysis of the target compound is available via the Chemotion repository: https://dx.doi.org/10.14272/UOEJOXKVDYCQAH-UHFFFAOYSA-N.1

3.2. Syntheses of 2,4,6-triazidobenzene-1,3,5-alkyltricarboxylates 3a-c, 3g, 2,4,6-Triazidobenzene-1,3,5-tricarboxylic acid (4) and Trimethyl-2,4,6-tribromobenzene-1,3,5-tricarboxylate (5)

Trimethyl-2,4,6-triazidobenzene-1,3,5-tricarboxylate (3a)

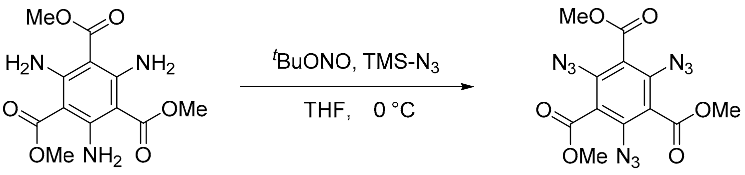

62 %

According to GP2, the reaction was carried out with trimethyl-2,4,6-triaminobenzene-1,3,5-tricarboxylate (**2a)** (1.00 g, 3.36 mmol, 1.00 equiv.); tert-butyl nitrite (3.12 g, 3.60 mL, 30.3 mmol, 9.00 equiv.); and trimethylsilyl azide (2.33 g, 2.65 mL, 20.2 mmol, 6.00 equiv.). Column chromatography (cyclohexane/ethyl acetate 20:1). The product **3a** was obtained as a yellow solid (784 mg, 2.09 mmol) in a yield of 62%.

R_f = 0.19 (cyclohexane/ethyl acetate 10:1). ^{1}H NMR (400 MHz, $CDCl_3$): δ [ppm] = 3.98 (s, 9H, C*H*$_3$). ^{13}C NMR (101 MHz, $CDCl_3$): δ [ppm] = 163.6, 137.7, 118.7, 53.2. MS (FAB, 3-NBA), m/z (%): 376 (17) $[M+H]^+$, 348 (15), 307 (25), 289 (14), 155 (28), 154 (100), 139 (19), 138 (36), 137 (83), 136 (70), 124 (15), 107 (26), 91 (15), 90 (20), 89 (17). HRMS–FAB *(m/z)*: $[M + H]^+$ calcd for $C_{12}H_{10}O_6N_9$: 376.0749; found 376.0749. IR (ATR, ṽ) = 2953 (w), 2122 (vs), 2102 (s), 1970 (w), 1962 (w), 1949 (w), 1921 (vw), 1741 (vs), 1723 (vs), 1561 (vs), 1453 (w), 1409 (vs), 1387 (s), 1327 (s), 1298 (vs), 1249 (s), 1213 (vs), 1194 (vs), 1181 (vs), 1157 (vs), 1118 (vs), 1007 (vs), 946 (m), 931 (vs), 875 (m), 860 (m), 790 (s), 744 (m), 687 (s), 657 (w), 612 (m), 584 (s), 555 (m), 524 (s), 483 (w), 460 (w), 443 (w), 421 (m), 388 (w) cm^{-1}.

The structure **3a** could be confirmed by single-crystal X-ray diffraction (see 5 Crystallographic Information).

Additional information on the chemical synthesis is available via the Chemotion repository: https://dx.doi.org/10.14272/reaction/SA-FUHFF-UHFFFADPSC-DHWXSONNLZ-UHFFFADPSC-NUHFF-NUHFF-NUHFF-ZZZ

Additional information on the analysis of the target compound is available via the Chemotion repository: https://dx.doi.org/10.14272/DHWXSONNLZCPDN-UHFFFAOYSA-N.1

Triethyl-2,4,6-triazidobenzene-1,3,5-tricarboxylate (3b)

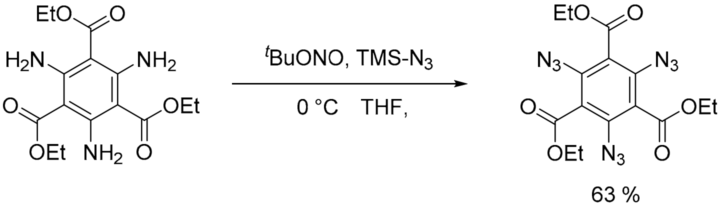

63 %

According to GP2, the reaction was carried out with triethyl-2,4,6-triaminobenzene-1,3,5-tricarboxylate (**2b)** (0.300 g, 0.884 mmol, 1.00 equiv.); tert-butyl nitrite (0.730 g, 7.08 mmol, 8.01 equiv.); and trimethylsilyl azide (0.544 g, 4.72 mmol, 5.34 equiv.). Column chromatography (cyclohexane/ethyl acetate 20:1). The product **3b** was obtained as a yellow solid (0.233 g, 0.559 mmol) in a yield of 63%.

R_f = 0.31 (cyclohexane/ethyl acetate 10:1). ^{1}H NMR (400 MHz, $CDCl_3$): δ [ppm] = 4.45 (q, J = 7.0 Hz, 6H, CH_2), 1.42 (t, J = 7.1 Hz, 9H, CH_3). ^{13}C NMR (101 MHz, $CDCl_3$): δ [ppm] = 163.3, 137.5, 119.6, 62.8, 14.0. MS (FAB, 3-NBA), m/z (%): 531 (28), 441 (33), 418 (26) $[M+H]^+$, 417 (30) $[M]^+$, 403 (17), 391 (38), 390 (86), 372 (63), 344 (23), 232 (19), 216 (17), 206 (17), 189 (21), 188 (52), 186 (25), 161 (20), 160 (30), 155 (25), 154 (83), 152 (17), 147 (47), 139 (20), 138 (35), 137 (100), 136 (95), 131 (18), 124 (20), 119 (19), 109 (28), 107 (28), 105 (20), 97 (46), 95 (29), 93 (28), 91 (25). HRMS–FAB *(m/z)*: $[M]^+$ calcd for $C_{15}H_{15}O_6N_9$: 417.1140; found 417.1141. IR (ATR, ṽ) = 2990 (w), 2965 (w), 2942 (w), 2113 (vs), 1731 (vs), 1713 (vs), 1561 (vs), 1462 (w), 1443 (w), 1422 (m), 1384 (w), 1377 (m), 1363 (s), 1307 (s), 1281 (vs), 1256 (vs), 1211 (vs), 1176 (vs), 1160 (vs), 1133 (s), 1115 (m), 1092 (s), 1017 (vs), 972 (s), 911 (m), 892 (m), 860 (s), 834 (m), 803 (w), 782 (m), 766 (w), 747 (w), 734 (m), 715 (m), 652 (w), 612 (s), 584 (m), 560 (w), 523 (s), 487 (w), 458 (w), 431 (w), 425 (w), 418 (w), 405 (w), 384 (m) cm^{-1}.

Additional information on the chemical synthesis is available via the Chemotion repository: https://dx.doi.org/10.14272/reaction/SA-FUHFF-UHFFFADPSC-ZFITVFRKCN-UHFFFADPSC-NUHFF-NUHFF-NUHFF-ZZZ

Additional information on the analysis of the target compound is available via the Chemotion repository: https://dx.doi.org/10.14272/ZFITVFRKCNNFJG-UHFFFAOYSA-N.1

Tri(isopropyl)-2,4,6-triazidobenzene-1,3,5-tricarboxylate (3c)

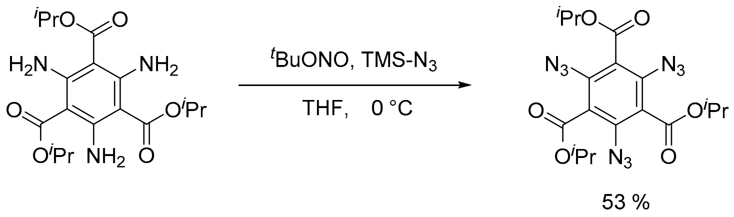

According to GP2, the reaction was carried out with triisopropyl-2,4,6-triaminobenzene-1,3,5-tricarboxylate (**2c)** (0.400 g, 1.05 mmol, 1.00 equiv.); tert-butyl nitrite (0.973 g, 9.44 mmol, 9.00 equiv.); and trimethylsilyl azide (0.725 g, 6.29 mmol, 6.00 equiv.). Column chromatography (cyclohexane/ethyl acetate 50:1). The product **3c** was obtained as a light-yellow oil (0.242 g, 0.526 mmol) in a yield of 53%.

R_f = 0.47 (cyclohexane/ethyl acetate 10:1). ^{1}H NMR (400 MHz, $CDCl_3$): δ [ppm] = 5.32 (sept, J = 6.1 Hz, 3H, CH), 1.41 (d, J = 6.3 Hz, 18H, CH_3). ^{13}C NMR (101 MHz, $CDCl_3$): δ [ppm] = 163.0, 137.4, 120.9, 71.5, 21.9. MS (FAB, 3-NBA), m/z (%): 547 (24), 541 (15), 503 (36), 497 (16), 460 (10) $[M+H]^+$, 459 (47) $[M]^+$, 453 (15), 415 (44), 371 (39), 327 (27), 188 (38), 186 (18), 178 (16), 176 (15), 162 (19), 161 (18), 160 (31), 159 (18), 155 (24), 154 (100), 152 (15), 149 (15), 147 (56), 139 (16), 138 (33), 137 (83), 136 (89), 131 (28), 129 (15), 128 (15), 120 (22), 119 (22), 117 (19), 115 (21), 109 (21), 107 (37), 105 (25), 97 (36), 95 (35), 93 (21), 91 (56), 90 (21), 89 (32). HRMS–FAB *(m/z)*: $[M]^+$ calcd for $C_{18}H_{21}O_6N_9$: 459.1609; found 459.1609. IR (ATR, ṽ) = 2980 (w), 2935 (w), 2876 (vw), 2140 (w), 2109 (vs), 1720 (vs), 1707 (vs), 1562 (vs), 1455 (w), 1415 (m), 1390 (w), 1374 (m), 1349 (w), 1337 (w), 1289 (vs), 1264 (vs), 1225 (vs), 1181 (s), 1164 (m), 1145 (s), 1096 (vs), 987 (s), 925 (m), 914 (s), 882 (m), 853 (w), 826 (s), 813 (m), 798 (w), 779 (w), 749 (w), 708 (w), 684 (w), 586 (m), 561 (w), 540 (w), 526 (m), 504 (w), 499 (w), 486 (w), 459 (w), 449 (w), 428 (m), 394 (vw), 387 (vw) cm^{-1}.

Additional information on the chemical synthesis is available via the Chemotion repository: https://dx.doi.org/10.14272/reaction/SA-FUHFF-UHFFFADPSC-ROXLWZLNQX-UHFFFADPSC-NUHFF-NUHFF-NUHFF-ZZZ

Additional information on the analysis of the target compound is available via the Chemotion repository: https://dx.doi.org/10.14272/ROXLWZLNQXFRED-UHFFFAOYSA-N.1

Tribenzyl-2,4,6-triazidobenzene-1,3,5-tricarboxylate (3g)

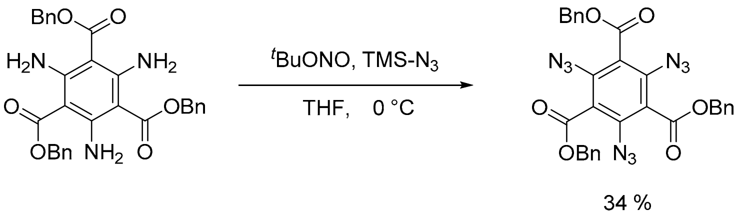

According to GP2, the reaction was carried out with tribenzyl-2,4,6-triaminobenzene-1,3,5-tricarboxylate (**2g)** (0.500 g, 0.951 mmol, 1.00 equiv.); tert-butyl nitrite (0.883 g, 1.02 mL, 8.56 mmol, 9.00 equiv.); and trimethylsilyl azide (0.658 g, 0.751 mL, 5.71 mmol, 6.00 equiv.). Column chromatography (cyclohexane/ethyl acetate 50:1 to 30:1). The product **3g** was obtained as a light-yellow oil (0.196 g, 0.325 mmol) in a yield of 34%.

R_f = 0.24 (cyclohexane/ethyl acetate 10:1). ^{1}H NMR (400 MHz, $CDCl_3$): δ [ppm] = 7.46–7.31 (m, 15H, Ar*H*), 5.37 (s, 6H, C*H*$_2$). ^{13}C NMR (101 MHz, $CDCl_3$): δ [ppm] = 163.2, 137.9, 134.3, 129.0, 129.0, 128.9, 119.2, 68.7. MS (FAB, 3-NBA), m/z (%): 603 (2) [M]$^+$, 496 (10) [$C_{23}H_{14}N_9O_5$]$^+$, 181 (7), 179 (4), 167 (4), 165 (5), 155 (5), 154 (15), 153 (4), 152 (4), 139 (7), 138 (7), 137 (10), 136 (11), 131 (5), 121 (2), 119 (4), 115 (4), 107 (7), 106 (6), 105 (6), 95 (12), 92 (22), 91 (100), 89 (11). HRMS–FAB *(m/z)*: [M + H]$^+$ calcd for $C_{30}H_{21}O_6N_9$: 603.1609; found 603.1612. IR (ATR, ṽ) = 3091 (vw), 3067 (vw), 3034 (vw), 2953 (vw), 2891 (vw), 2112 (vs), 1725 (vs), 1564 (vs), 1497 (w), 1455 (m), 1417 (s), 1370 (s), 1279 (vs), 1255 (vs), 1198 (vs), 1157 (s), 1123 (vs), 1081 (m), 1028 (m), 997 (s), 976 (s), 939 (s), 908 (s), 861 (m), 849 (m), 826 (m), 747 (vs), 696 (vs), 613 (m), 579 (m), 527 (m), 503 (m), 462 (m) cm^{-1}.

Additional information on the chemical synthesis is available via the Chemotion repository: https://dx.doi.org/10.14272/reaction/SA-FUHFF-UHFFFADPSC-KOPNHBUFTQ-UHFFFADPSC-NUHFF-NUHFF-NUHFF-ZZZ

Additional information on the analysis of the target compound is available via the Chemotion repository: https://dx.doi.org/10.14272/KOPNHBUFTQTRII-UHFFFAOYSA-N.1

2,4,6-Triazidobenzene-1,3,5-tricarboxylic acid (4)

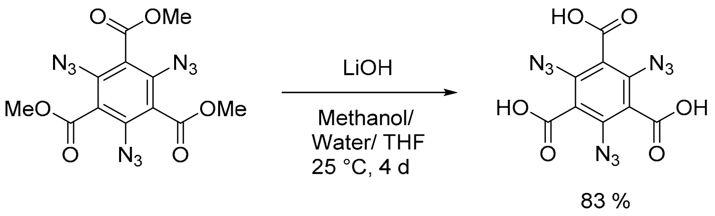

Trimethyl-2,4,6-triazidobenzene-1,3,5-tricarboxylate (**3a**) (200 mg, 533 µmol, 1.00 equiv.) and lithium hydroxide (127 mg, 5.33 mmol, 10.0 equiv.) were stirred at 25 °C for 4 d in methanol (2.00 mL), oxidane (2.00 mL) and oxolane (1.00 mL). 1M HCl was added to achieve a pH of 4. EtOAc was added and the phases were separated. The aqueous phase was extracted three times with EtOAc. The combined organic layers were dried over sodium sulfate. The solvent was removed under reduced pressure to give 2,4,6-triazidobenzene-1,3,5-tricarboxylic acid (**4**, 147 mg, 441 µmol, 83% yield) as an off-white powder.

^{1}H NMR (400 MHz, DMSO-d$_6$): δ [ppm] = 14.50 (br, 3H, O*H*). ^{13}C NMR (101 MHz, DMSO-d$_6$): δ [ppm] = 164.3, 134.9, 121.2. MS (EI, 70 eV, 30 °C), m/z (%): 334 (6) [M+H]+, 307 (13), 219 (28), 217 (29), 155 (36), 154 (100), 147 (26), 139 (22), 138 (42), 137 (72), 136 (85), 131 (21), 121 (21), 119 (24), 109 (27), 107 (38), 105 (27), 97 (36), 95 (49), 93 (22), 91 (54), 89 (23). HRMS–EI (m/z): [M]+ calcd for $C_9H_4O_6N_9$: 334.0281; found 334.0279. IR (ATR, ṽ) = 3387 (w), 3097 (w), 2908 (w), 2649 (w), 2138 (vs), 2112 (vs), 1758 (w), 1686 (vs), 1545 (vs), 1428 (m), 1371 (s), 1288 (vs), 1259 (vs), 1244 (vs), 1214 (vs), 1173 (vs), 1147 (s), 973 (s), 867 (s), 860 (s), 837 (s), 823 (s), 792 (m), 756 (m), 735 (s), 686 (s), 653 (s), 636 (vs), 602 (vs), 577 (s), 528 (vs), 518 (s), 483 (s), 458 (vs).

Additional information on the chemical synthesis is available via the Chemotion repository: https://dx.doi.org/10.14272/reaction/SA-FUHFF-UHFFFADPSC-LSJVJAOCLW-UHFFFADPSC-NUHFF-NUHFF-NUHFF-ZZZ

Additional information on the analysis of the target compound is available via the Chemotion repository: https://dx.doi.org/10.14272/LSJVJAOCLWCAPE-UHFFFAOYSA-N.1

Trimethyl-2,4,6-tribromobenzene-1,3,5-tricarboxylate (5)

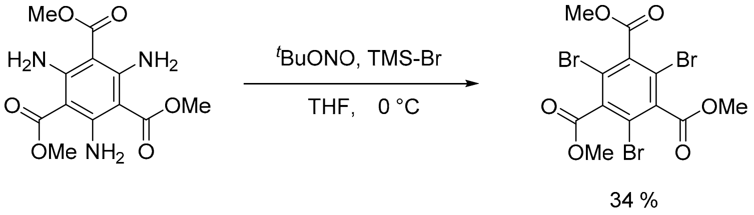

Under an argon atmosphere, trimethyl 2,4,6-triaminobenzene-1,3,5-tricarboxylate **(2a)** (1.00 g, 3.36 mmol, 1.00 equiv.) was dissolved in dry acetonitrile (25 mL) and cooled to 0 °C. Tert-butyl nitrite (3.12 g, 3.60 mL, 30.3 mmol, 9.00 equiv.) was added dropwise in alternation with bromo(trimethyl)silane (6.18 g, 5.33 mL, 40.4 mmol, 12.0 equiv.). After complete addition, the mixture was slowly warmed up to room temperature and stirred for 16 h. The resulting bromine gas in the glassware was purged into a wash bottle that was filled with a saturated aqueous solution of $Na_2S_2O_3$, and the dissolved bromine was quenched

by the addition of $Na_2S_2O_3$ solution (30 mL) to the reaction mixture. Then, CH_2Cl_2 (30 mL) was added and the layers were separated. The organic layer was washed successively with an aqueous solution of HCl (30 mL, 1M); water (30 mL); and brine (30 mL), followed by drying over Na_2SO_4. After filtration, the solvent was removed under reduced pressure and the residue was purified by column chromatography (100% CH_2Cl_2) to give trimethyl 2,4,6-tribromobenzene-1,3,5-tricarboxylate (**5**) (561 mg, 1.15 mmol, 34% yield) as a colorless solid.

R_f = 0.60 (CH_2Cl_2). ^{1}H NMR (400 MHz, $CDCl_3$): δ [ppm] = 3.99 (s, 9H, C*H*$_3$). ^{13}C NMR (100 MHz, $CDCl_3$): δ [ppm] = 165.0, 138.3, 117.8, 53.5. MS (FAB, 3-NBA), m/z (%): 493 (15) [$C_{12}H_9O_6{}^{81}Br_3$+H]+, 491 (46) [$C_{12}H_9O_6{}^{79}Br^{81}Br_2$+H]+, 489 (45) [M+H]+, 487 (16) [$C_{12}H_9O_6{}^{79}Br_3$+H]+, 461 (15), 459 (48), 457 (50), 455 (16), 155 (33), 154 (94), 52 (22), 39 (35), 138 (50), 137 (88), 136 (100), 23 (22), 121 (26), 120 (25), 119 (25), 109 (36), 107 (46), 105 (31), 97 (41), 95 (67), 93 (33), 91 (62), 89 (27). HRMS–FAB *(m/z)*: [M + H]$^+$ calcd for $C_{12}H_{10}O_6{}^{79}Br_3$: 486.8022; found 486.8021, HRMS–FAB *(m/z)*: [M + H]$^+$ calcd for $C_{12}H_{10}O_6$79$Br_1{}^{81}Br_2$: 490.7981; found 490.7983. IR (ATR, ṽ) = 2956 (w), 1721 (vs), 1548 (s), 1451 (w), 1434 (m), 1366 (w), 1344 (m), 1228 (vs), 1179 (vs), 1153 (s), 1075 (w), 983 (vs), 884 (vs), 823 (w), 803 (w), 768 (w), 756 (w), 742 (w), 664 (w), 537 (s), 482 (w), 421 (m) cm^{-1}. EA ($C_{12}H_9Br_3O_6$): Calcd C 29.48; H 1.86. Found C 29.75; H 1.82.

Additional information on the chemical synthesis is available via the Chemotion repository: https://dx.doi.org/10.14272/reaction/SA-FUHFF-UHFFFADPSC-BDZRYAKTXQ-UHFFFADPSC-NUHFF-NUHFF-NUHFF-ZZZ

Additional information on the analysis of the target compound is available via the Chemotion repository: https://dx.doi.org/10.14272/BDZRYAKTXQRGMS-UHFFFAOYSA-N.1

3.3. Click reactions of azides 3a and 3b

Trimethyl-2,4,6-tris(4-phenyl-1*H*-1,2,3-triazol-1-yl)benzene-1,3,5-tricarboxylate (6a-Ph)

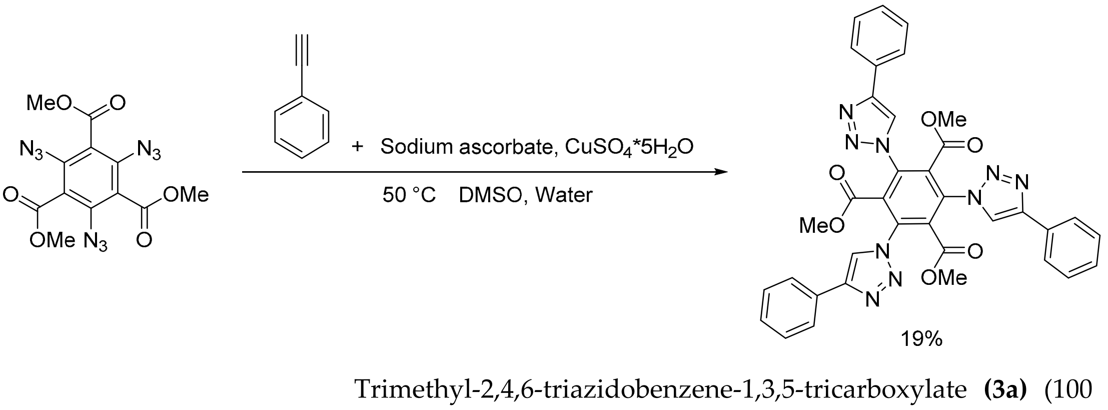

Trimethyl-2,4,6-triazidobenzene-1,3,5-tricarboxylate **(3a)** (100 mg, 267 µmol, 1.00 equiv.) and ethynylbenzene (95.3 mg, 933 µmol, 3.50 equiv.) were solved under argon in degassed DMSO (2.50 mL). Copper sulfate pentahydrate (13.3 mg, 53.3 µmol, 0.200 equiv.) and sodium ascorbate (26.4 mg, 133 µmol, 0.500 equiv.) were solved in degassed water (500 µL) and degassed DMSO (3.00 mL). The mixture was added dropwise to the ethynylbenzene solution. The mixture was stirred at 25 °C for 1 d, then 1 d at 50 °C. After cooling to 25 °C, ethyl acetate (20 mL) and water (20 mL) were added, and the phases were separated. The

organic layer was washed with brine (20 mL) and then dried over sodium sulfate. The solvent was removed under reduced pressure and the residue was purified via flash chromatography (cyclohexane/ethyl acetate 10:1 to 4:1) to give trimethyl-2,4,6-tris(4-phenyl-1H-1,2,3-triazol-1-yl)benzene-1,3,5-tricarboxylate (**6a-Ph**) (34.0 mg, 49.8 μmol, 19% yield) as a colorless powder.

R_f = 0.13 (cyclohexane/ethyl acetate 3:1). ^{1}H NMR (400 MHz, $CDCl_3$): δ [ppm] = 8.26 (s, 3H, C*H*), 7.94 – 7.87 (m, 6H, Ar*H*), 7.53 – 7.45 (m, 6H, Ar*H*), 7.49 – 7.37 (m, 3H, Ar*H*), 3.53 (s, 9H, C*H*$_3$). ^{13}C NMR (101 MHz, $CDCl_3$): δ [ppm] = 161.81, 148.58, 135.18, 130.95, 129.30, 129.26, 129.20, 126.20, 123.01, 54.12. MS (FAB, 3-NBA), m/z (%): 682 (9) [M+H]$^+$, 663 (8), 313 (9), 307 (25), 289 (14), 217 (10), 156 (8), 155 (32), 154 (100), 153 (16), 152 (9), 139 (20), 138 (63), 137 (98), 136 (63), 135 (8), 129 (8), 124 (9), 123 (8), 121 (10), 120 (12), 119 (8), 111 (8), 109 (16), 108 (13), 107 (23), 105 (12), 97 (20), 95 (20), 93 (17), 91 (17), 90 (21), 89 (15). HRMS–FAB *(m/z)*: [M + H]$^+$ calcd for $C_{36}H_{28}O_6N_9$: 682.2157; found 682.2156. IR (ATR, ṽ) = 3125 (vw), 3080 (w), 3065 (w), 2953 (w), 1734 (vs), 1588 (m), 1493 (w), 1475 (m), 1451 (w), 1432 (s), 1391 (w), 1374 (w), 1341 (w), 1310 (vw), 1283 (w), 1235 (vs), 1201 (m), 1160 (w), 1142 (w), 1072 (w), 1014 (vs), 984 (s), 912 (w), 888 (m), 840 (m), 807 (w), 789 (vw), 765 (vs), 708 (w), 693 (vs), 664 (w), 615 (w), 586 (w), 545 (w), 511 (m), 482 (w), 456 (w), 426 (w), 416 (w), 394 (w), 387 (w) cm^{-1}.

Additional information on the chemical synthesis is available via the Chemotion repository: https://dx.doi.org/10.14272/reaction/SA-FUHFF-UHFFFADPSC-SSVWEQXUTX-UHFFFADPSC-NUHFF-NUHFF-NUHFF-ZZZ

Additional information on the analysis of the target compound is available via the Chemotion repository: https://dx.doi.org/10.14272/SSVWEQXUTXDPTL-UHFFFAOYSA-N.1

Trimethyl-2,4,6-tris(4-(4-bromophenyl)-1*H*-1,2,3-triazol-1-yl)benzene-1,3,5-tricarboxylate (6a-C_6H_4Br)

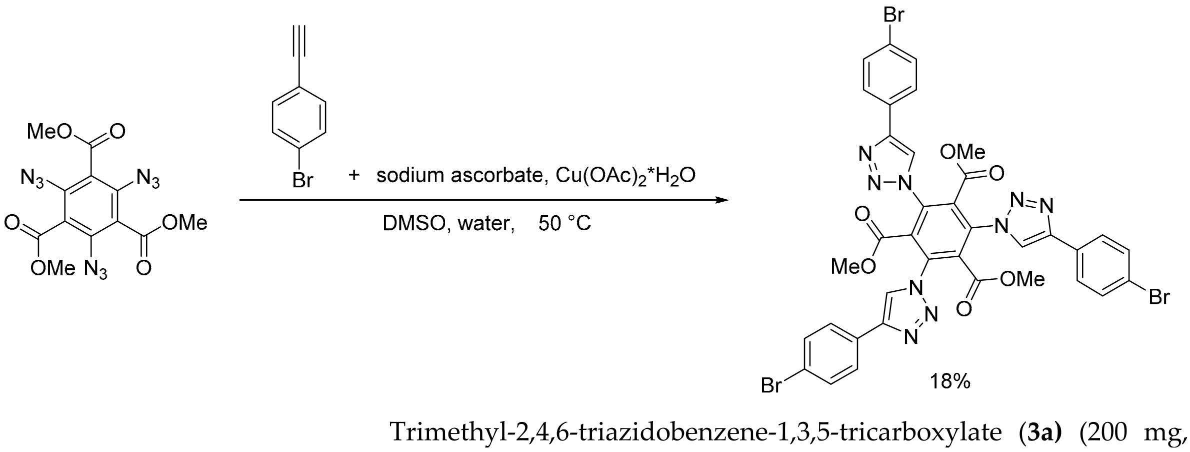

Trimethyl-2,4,6-triazidobenzene-1,3,5-tricarboxylate (**3a)** (200 mg, 533 μmol, 1.00 equiv.); $Cu(OAc)_2$*H_2O (16.0 mg, 79.9 μmol, 0.150 equiv.); 1-bromo-4-ethynylbenzene (338 mg, 1.87 mmol, 3.50 equiv.); and sodium ascorbate (31.7 mg, 160 μmol, 0.300 equiv.) were solved in degassed DMSO (10.0 mL) and degassed water (1.00 mL) in a vial. The mixture was stirred for 2 d at 30 °C, then 1 d at 50 °C, followed by 3 d at 60 °C. After cooling to 25 °C, ethyl acetate (40 mL) and water (40 mL) were added, and the phases were separated. The organic layer was washed with brine (40 mL) and then dried over sodium sulfate. The solvent was removed under reduced pressure and the residue was purified via flash chromatography

(cyclohexane/ethyl acetate 10:1 to 4:1) to give trimethyl-2,4,6-tris(4-(4-bromophenyl)-1H-1,2,3-triazol-1-yl)benzene-1,3,5-tricarboxylate **(6a-C_6H_4Br)** (90.0 mg, 98.0 µmol, 18% yield) as a light-yellow solid.

*R*f = 0.27 (cyclohexane/ethyl acetate 3:1). ^{1}H NMR (400 MHz, $CDCl_3$): δ [ppm] = 8.27 (s, 3H, C*H*), 7.79 – 7.73 (m, 6H, Ar*H*), 7.63 – 7.58 (m, 6H, Ar*H*), 3.52 (s, 9H, C*H*3). ^{13}C NMR (101 MHz, $CDCl_3$): δ [ppm] = 161.7, 147.6, 135.1, 132.5, 131.0, 128.1, 127.6, 123.3, 123.1, 54.2. MS (FAB, 3-NBA), m/z (%): 918 (1), 664 (32), 663 (75) $[C_{35}H_{21}N_9O_6]^{3*}$, 662 (44), 661 (15), 648 (19), 647 (41), 531 (10), 530 (22), 307 (20), 289 (13), 219 (12), 206 (11), 191 (14), 167 (10), 155 (30), 154 (100), 152 (13), 147 (15), 139 (20), 138 (37), 137 (54), 136 (73), 131 (12), 128 (11), 124 (10), 123 (10), 121 (14), 120 (14), 119 (16), 117 (10), 115 (14), 109 (18), 107 (29), 105 (19), 97 (18), 95 (31), 93 (16), 91 (40), 90 (13), 89 (21). HRMS–FAB (m/z): $[M + H]^+$ calcd for $C_{36}H_{25}O_6N_9{}^{79}Br_3$: 915.9472; found 915.9473. IR (ATR, ṽ) = 3122 (w), 2952 (w), 2924 (w), 2850 (w), 1731 (vs), 1591 (m), 1553 (w), 1472 (s), 1432 (s), 1408 (w), 1373 (w), 1344 (w), 1298 (w), 1239 (vs), 1186 (m), 1140 (m), 1098 (w), 1068 (m), 1024 (vs), 1009 (vs), 982 (s), 972 (m), 888 (m), 820 (vs), 764 (m), 742 (w), 720 (m), 701 (w), 686 (w), 659 (w), 616 (m), 605 (m), 510 (s), 482 (w), 467 (w), 445 (m), 415 (w), 401 (w), 380 (m) cm^{-1}.

Additional information on the chemical synthesis is available via the Chemotion repository: https://dx.doi.org/10.14272/reaction/SA-FUHFF-UHFFFADPSC-HIJXLLJBVN-UHFFFADPSC-NUHFF-NUHFF-NUHFF-ZZZ

Additional information on the analysis of the target compound is available via the Chemotion repository: https://dx.doi.org/10.14272/HIJXLLJBVNQJME-UHFFFAOYSA-N.1

Triethyl-2,4,6-tris(4-phenyl-1*H*-1,2,3-triazol-1-yl)benzene-1,3,5-tricarboxylate (6b-Ph)

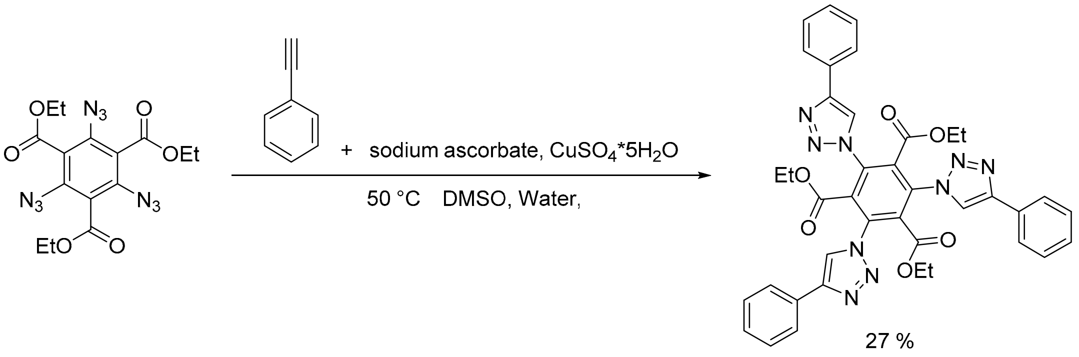

Triethyl-2,4,6-triazidobenzene-1,3,5-tricarboxylate **(3b)** (100 mg, 240 µmol, 1.00 equiv.) and ethynylbenzene (85.7 mg, 839 µmol, 3.50 equiv.) were solved under argon in degassed DMSO (2.50 mL). Copper sulfate pentahydrate (8.97 mg, 35.9 µmol, 0.150 equiv.) and sodium ascorbate (14.2 mg, 71.9 µmol, 0.300 equiv.) were solved in degassed water (500 µL) and degassed DMSO (2.50 mL). The mixture was added dropwise to the ethynylbenzene-solution. The mixture was stirred at 50 °C for 3 d. After cooling to 25 °C, ethyl acetate (20 mL) and water (20 mL) were added, and the phases were separated. The organic layer was washed with brine (20 mL) and then dried over sodium sulfate. The solvent was removed under reduced pressure and the residue was purified via flash chromatography (cyclohexane/ethyl acetate 20:1 to 4:1) to give triethyl-2,4,6-tris(4-phenyl-

1H-1,2,3-triazol-1-yl)benzene-1,3,5-tricarboxylate (**6b-Ph**) (46.0 mg, 63.6 µmol, 27% yield) as a light-yellow solid.

R_f = 0.47 (cyclohexane/ethyl acetate 4:1). ^{1}H NMR (400 MHz, $CDCl_3$): δ [ppm] = 8.26 (s, 3H, C*H*), 7.92–7.86 (m, 6H, Ar*H*), 7.51–7.45 (m, 6H, Ar*H*), 7.44–7.38 (m, 3H, Ar*H*), 3.99 (q, J = 7.2 Hz, 6H, CH_2), 0.92 (t, J = 7.1 Hz, 9H, CH_3). ^{13}C NMR (101 MHz, $CDCl_3$): δ [ppm] = 161.3, 148.4, 134.9, 131.7, 129.4, 129.3, 129.1, 126.2, 123.3, 63.9, 13.7. MS (FAB, 3-NBA), m/z (%): 725 (18), 724 (38) $[M+H]^+$, 307 (18), 217 (48), 155 (32), 154 (100), 139 (21), 138 (41), 137 (74), 136 (78), 120 (15), 107 (27), 105 (31), 97 (14), 95 (18), 91 (33), 90 (16), 89 (25). HRMS–FAB (m/z): $[M + H]^+$ calcd for $C_{39}H_{34}O_6N_9$: 724.2627; found 724.2625. IR (ATR, ṽ) = 3139 (w), 3125 (vw), 3102 (vw), 3080 (w), 3033 (vw), 2982 (w), 2965 (vw), 2936 (vw), 1728 (vs), 1588 (m), 1560 (vw), 1492 (w), 1475 (m), 1462 (m), 1449 (w), 1391 (w), 1375 (w), 1333 (w), 1298 (w), 1283 (w), 1235 (vs), 1200 (m), 1177 (w), 1143 (w), 1096 (w), 1072 (w), 1014 (vs), 933 (w), 915 (w), 858 (w), 834 (w), 765 (vs), 707 (w), 693 (vs), 662 (w), 613 (w), 592 (w), 585 (w), 558 (vw), 537 (w), 507 (w), 465 (w), 448 (w), 422 (vw), 415 (vw), 398 (w), 387 (vw) cm^{-1}.

Additional information on the chemical synthesis is available via the Chemotion repository: https://dx.doi.org/10.14272/reaction/SA-FUHFF-UHFFFADPSC-UOEJOXKVDY-UHFFFADPSC-NUHFF-NUHFF-NUHFF-ZZZ

Additional information on the analysis of the target compound is available via the Chemotion repository: https://dx.doi.org/10.14272/UOEJOXKVDYCQAH-UHFFFAOYSA-N.1

*3.4. Alkylation of amine **2a***

Trimethyl-2,4,6-tris(dimethylamino)benzene-1,3,5-tricarboxylate (7a)

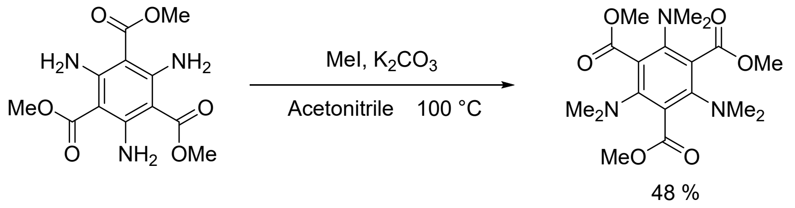

A 15 mL vial was charged with trimethyl-2,4,6-triaminobenzene-1,3,5-tricarboxylate **(2a)** (200 mg, 673 µmol, 1.00 equiv.) and potassium carbonate (558 mg, 4.04 mmol, 6.00 equiv.). Acetonitrile (2.00 mL) and iodomethane (859 mg, 377 µL, 6.06 mmol, 9.00 equiv.) were added. The pressure tube was sealed and heated to 100 °C for 16 h. After the reaction mixture was cooled to room temperature, it was diluted with dichloromethane (30 mL) and washed with water (2 x 30 mL), then with brine (30 mL). The organic layer was dried over sodium sulfate, and the solvent was removed under reduced pressure. The crude product was purified via flash chromatography on silica gel (cyclohexane/ethyl acetate 20:1) to give trimethyl-2,4,6-tris(dimethylamino)benzene-1,3,5-tricarboxylate **(7a)** (124 mg, 325 µmol, 48% yield) as a light-yellow powder.

R_f = 0.47 (cyclohexane/ethyl acetate 4:1). ^{1}H NMR (400 MHz, $CDCl_3$): δ [ppm] = 3.87 (s, 9H, OCH_3), 2.70 (s, 18H, N(CH_3)$_2$). ^{13}C NMR (101 MHz, $CDCl_3$): δ [ppm] = 168.7, 150.5, 129.2, 52.2, 43.8. MS (FAB, 3-NBA), m/z (%): 383 (20), 382 (100) $[M+H]^+$, 381 (92) $[M]^+$, 380 (78) $[M-H]^+$, 350 (56), 322 (36), 154 (26), 137 (16), 136 (18). HRMS–FAB (m/z): [M]+ calcd for

$C_{18}H_{27}O_6N_3$: 381.1894; found 381.1896. IR (ATR, ṽ) = 2995 (vw), 2948 (w), 2925 (w), 2895 (w), 2867 (w), 2850 (w), 2798 (w), 1718 (vs), 1550 (s), 1483 (w), 1449 (m), 1435 (w), 1404 (m), 1334 (w), 1298 (m), 1289 (m), 1217 (vs), 1203 (vs), 1125 (w), 1098 (w), 1058 (m), 986 (vs), 955 (w), 939 (w), 890 (w), 820 (m), 810 (w), 751 (w), 721 (w), 639 (w), 619 (m), 484 (w), 439 (w), 429 (w), 382 (w) cm^{-1}.

Additional information on the chemical synthesis is available via the Chemotion repository: https://dx.doi.org/10.14272/reaction/SA-FUHFF-UHFFFADPSC-JHAXFHTYOK-UHFFFADPSC-NUHFF-NUHFF-NUHFF-ZZZ

Additional information on the analysis of the target compound is available via the Chemotion repository: https://dx.doi.org/10.14272/JHAXFHTYOKXUIN-UHFFFAOYSA-N.1

Trimethyl-2,4,6-tris(diethylamino)benzene-1,3,5-tricarboxylate (7b)

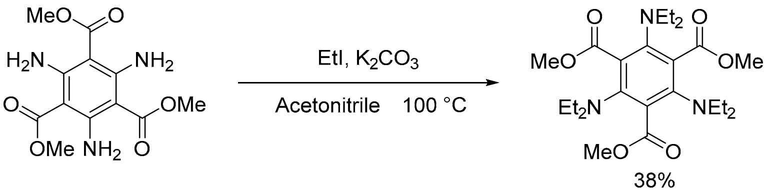

A 15 mL pressure tube was charged with trimethyl-2,4,6-triaminobenzene-1,3,5-tricarboxylate **(2a)** (400 mg, 1.35 mmol, 1.00 equiv.); dipotassium carbonate (1.12 g, 8.07 mmol, 6.00 equiv.); and acetonitrile (13.0 mL). Iodoethane (2.52 g, 1.30 mL, 16.1 mmol, 12.0 equiv.) was added and the mixture was heated to 100 °C for 2 d. The reaction mixture was cooled to room temperature, diluted with dichloromethane (30 mL) and washed with water (2 x 30 mL), then with brine (30 mL). Chromatographic separation with silica gel (cyclohexane/ethyl acetate 10:1) gave trimethyl-2,4,6-tris(diethylamino)benzene-1,3,5-tricarboxylate **(7b)** (236 mg, 507 µmol, 38% yield) as a light-yellow powder.

R_f = 0.71 (cyclohexane/ethyl acetate 4:1). ^1H NMR (400 MHz, $CDCl_3$): δ [ppm] = 3.82 (s, 9H, OCH_3), 2.89 (q, J = 7.1 Hz, 12H, CH_2), 0.97 (t, J = 7.1 Hz, 18H, CH_3). ^{13}C NMR (101 MHz, $CDCl_3$): δ [ppm] = 168.9, 147.9, 134.0, 52.1, 48.2, 14.3. MS (FAB, 3-NBA), m/z (%): 478 (20), 467 (12), 466 (48), 465 (41) $[M]^+$, 464 (100), 450 (16), 434 (34), 406 (53). HRMS–FAB (m/z): [M]+ calcd for $C_{24}H_{39}O_6N_3$: 465.2833; found 465.2835. HRMS–FAB (m/z): [M + H]+ calcd for $C_{24}H_{40}O_6N_3$: 466.2912 found 466.2912. IR (ATR, ṽ) = 2973 (w), 2949 (w), 2934 (w), 2846 (w), 1728 (vs), 1554 (m), 1446 (w), 1421 (m), 1378 (m), 1360 (w), 1334 (w), 1299 (w), 1225 (vs), 1197 (vs), 1173 (vs), 1120 (m), 1088 (w), 1064 (m), 1006 (vs), 975 (m), 874 (w), 816 (w), 799 (w), 771 (w), 741 (w), 727 (vw), 629 (m), 503 (w), 426 (vw) cm^{-1}.

Additional information on the chemical synthesis is available via the Chemotion repository: https://dx.doi.org/10.14272/reaction/SA-FUHFF-UHFFFADPSC-RPBKGFQGWR-UHFFFADPSC-NUHFF-NUHFF-NUHFF-ZZZ

Additional information on the analysis of the target compound is available via the Chemotion repository: https://dx.doi.org/10.14272/RPBKGFQGWRGASM-UHFFFAOYSA-N.1

Trimethyl-2,4,6-tris(diethylamino)benzene-1,3,5-tricarboxylate (7c)

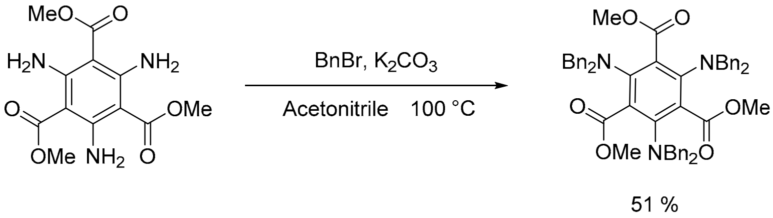

A 50 mL pressure tube was charged with trimethyl-2,4,6-triaminobenzene-1,3,5-tricarboxylate **(2a)** (400 mg, 1.35 mmol, 1.00 equiv.); dipotassium carbonate (1.12 g, 8.07 mmol, 6.00 equiv.); bromomethylbenzene (2.76 g, 16.1 mmol, 12.0 equiv.); and acetonitrile (20.0 mL). The pressure tube was sealed and heated to 100 °C for 2 d. After the reaction mixture was cooled to room temperature, it was diluted with dichloromethane (30 mL) and washed with water (2 x 30 mL), then with brine (30 mL). The organic layer was dried over sodium sulfate, and the solvent was removed under reduced pressure. The crude product was purified via flash chromatography on silica gel (cyclohexane/ethyl acetate 10:1) to give trimethyl-2,4,6-tris(diethylamino)benzene-1,3,5-tricarboxylate **(7c)** (571 mg, 681 µmol, 51% yield) as a light-yellow powder.

R_f = 0.68 (cyclohexane/ethyl acetate 4:1). ^{1}H NMR (400 MHz, $CDCl_3$): δ [ppm] = 7.27–7.20 (m, 18H, Ar*H*), 7.16–7.13 (m, 12H, Ar*H*), 4.04 (s, 12H, CH_2), 3.67 (s, 9H, CH_3). ^{13}C NMR (101 MHz, $CDCl_3$): δ [ppm] = 168.6, 148.4, 138.3, 132.5, 129.4, 128.1, 127.1, 57.3, 52.3. MS (FAB, 3-NBA), m/z (%): 838 (10) $[M+H]^+$, 746 (24), 91 (100). HRMS–FAB *(m/z)*: $[M+H]^+$ calcd for $C_{54}H_{52}O_6N_3$: 838.3851; found 838.3853. IR (ATR, ṽ) = 3087 (vw), 3064 (vw), 3030 (w), 3006 (vw), 2949 (w), 2924 (w), 2890 (w), 2871 (w), 2847 (w), 2830 (w), 2809 (vw), 1949 (vw), 1737 (vs), 1711 (vs), 1604 (w), 1585 (w), 1551 (m), 1494 (m), 1479 (w), 1452 (m), 1438 (w), 1415 (m), 1390 (w), 1363 (m), 1332 (w), 1302 (w), 1249 (s), 1227 (vs), 1211 (vs), 1183 (s), 1153 (w), 1118 (s), 1075 (m), 1028 (w), 993 (m), 958 (s), 919 (w), 911 (w), 891 (w), 844 (w), 832 (w), 795 (vw), 739 (vs), 730 (vs), 697 (vs), 656 (s), 620 (w), 611 (m), 592 (w), 572 (w), 555 (w), 541 (w), 507 (m), 487 (s), 473 (m), 460 (m), 441 (w), 407 (w), 394 (w) cm^{-1}.

Additional information on the chemical synthesis is available via the Chemotion repository: https://dx.doi.org/10.14272/reaction/SA-FUHFF-UHFFFADPSC-UTSLQYVRSX-UHFFFADPSC-NUHFF-NUHFF-NUHFF-ZZZ

Additional information on the analysis of the target compound is available via the Chemotion repository: https://dx.doi.org/10.14272/UTSLQYVRSXYTGW-UHFFFAOYSA-N.1

4. Crystallographic Information

Crystal Structure Determinations of 2a, 2b, 2c and 3a

The single-crystal X-ray diffraction study was carried out on a Bruker D8 Venture diffractometer with a PhotonII detector at 123(2) K; 173(2) K; or 298(2) K using Cu-K radiation (= 1.54178 Å) or Mo-K radiation (= 0.71073 Å). Dual space methods (SHELXT for **5a**) [G. M. Sheldrick, *Acta Crystallogr.* 2015, **A71**, 3–8] were used for the structure solution, and refinement was carried out using SHELXL-2014 (full-matrix least-squares on F^2) [G. M. Sheldrick, *Acta Crystallogr.* 2015, **C71**, 3–8]. Hydrogen atoms were localized by difference electron density determination and refined using a riding model (H(n) free). Semi-empirical absorption corrections were applied. Due to the bad quality of the crystal of **3a** (only 6 of 12 runs were used with a completeness of approx. 82%), the data were not deposited with The Cambridge Crystallographic Data Centre, but the molecular structure could be unambiguously determined.

2a: Colorless crystals, $C_{12}H_{15}N_3O_6$, M_r = 297.27, crystal size 0.35 × 0.25 × 0.10 mm, monoclinic, space group *C*2/c (no. 15). *a* = 24.1680(6) Å; *b* = 6.4859(2) Å; *c* = 17.8719(5) Å; β = 110.312(1)°; *V* = 2627.24(3) Å^3; *Z* = 8; ρ = 1.503 Mg/m^{-3}; μ(Mo-K$_\alpha$) = 0.12 mm^{-1}; *F*(000) = 1248; *T* = 173 K; $2\theta_{max}$ = 55.0°. A total of 28104 reflections, of which 3027 were independent (R_{int} = 0.051), 211 parameters, six restraints. R_1 = 0.037 (for 2763 I > 2σ(I)); wR_2 = 0.105 (all data); *S* = 1.07, largest diff. peak / hole = 0.32 / -0.22 e Å^{-3}.

2b: Colorless crystals, $C_{15}H_{21}N_3O_6$, M_r = 339.35, crystal size 0.18 × 0.12 × 0.04 mm, monoclinic, space group *P*2$_1$/n (no. 14). *a* = 9.8213(3) Å; *b* = 13.5768(4) Å; *c* = 11.9177(3) Å; β = 98.061(1)°; *V* = 1573.43(8) Å^3; *Z* = 4; ρ = 1.433 Mg/m^{-3}; μ(Cu-K$_\alpha$) = 0.94 mm^{-1}; *F*(000) = 720; *T* = 123 K; $2\theta_{max}$ = 144.4°. A total of 16668 reflections, of which 3100 were independent (R_{int} = 0.026), 235 parameters, six restraints. R_1 = 0.031 (for 2887 I > 2σ(I)); wR_2 = 0.083 (all data); *S* = 1.05, largest diff. peak / hole = 0.24 / -0.20 e Å^{-3}.

2c: Colorless crystals, $C_{18}H_{27}N_3O_6$, M_r = 381.42, crystal size 0.30 × 0.20 × 0.15 mm, monoclinic, space group *P*2$_1$/n (no. 14). *a* = 11.5118(5) Å; *b* = 14.5038(6) Å; *c* = 12.9688(5) Å; β = 115.290(1)°; *V* = 1957.80(14) Å^3; *Z* = 4; ρ = 1.294 Mg/m^{-3}; μ(Cu-K$_\alpha$) = 0.81 mm^{-1}; *F*(000) = 816; *T* = 123 K; $2\theta_{max}$ = 144.4°. A total of 26326 reflections, of which 3820 were independent (R_{int} = 0.024), 262 parameters, six restraints. R_1 = 0.033 (for 3778 I > 2σ(I)); wR_2 = 0.086 (all data); *S* = 1.07, largest diff. peak / hole = 0.28 / -0.16 e Å^{-3}.

3a: Yellow crystals, $C_{12}H_9N_9O_6$, M_r = 375.28, crystal size 0.16 × 0.10 × 0.03 mm, triclinic, space group *P-1* (no. 2). *a* = 8.173(3) Å; *b* = 10.031(4) Å; *c* = 10.833(4) Å; α = 75.25(2)°; β = 73.84(2)°; γ = 71.15(2)°; *V* = 793.9(5) Å^3; *Z* = 2; ρ = 1.570 Mg/m^{-3}; μ(Cu-K$_\alpha$) = 1.13 mm^{-1}; *F*(000) = 348; *T* = 298 K; $2\theta_{max}$ = 144.0°. A total of 6393 reflections, of which 2573 were independent (R_{int} = 0.055), 248 parameters, 189 restraints. R_1 = 0.069 (for 1924 I > 2σ(I)); wR_2 = 0.217 (all data); *S* = 1.05, largest diff. peak / hole = 0.38 / -0.34 e Å^{-3}. Due to the bad quality of the crystal of **3a** (see cif.file for details, completeness approx. 82%), the data were not deposited with The Cambridge Crystallographic Data Centre.

CCDC 2102766 (**2a**), 2102767 (**2b**) and 2102768 (**2c**) contain the supplementary crystallographic data for this paper. These data can be obtained free of charge from The Cambridge Crystallographic Data Centre via www.ccdc.cam.ac.uk/data_request/cif. Due to the bad quality of the data of **3a** (completeness approx. 82%), the data were not deposited with The Cambridge Crystallographic Data Centre.

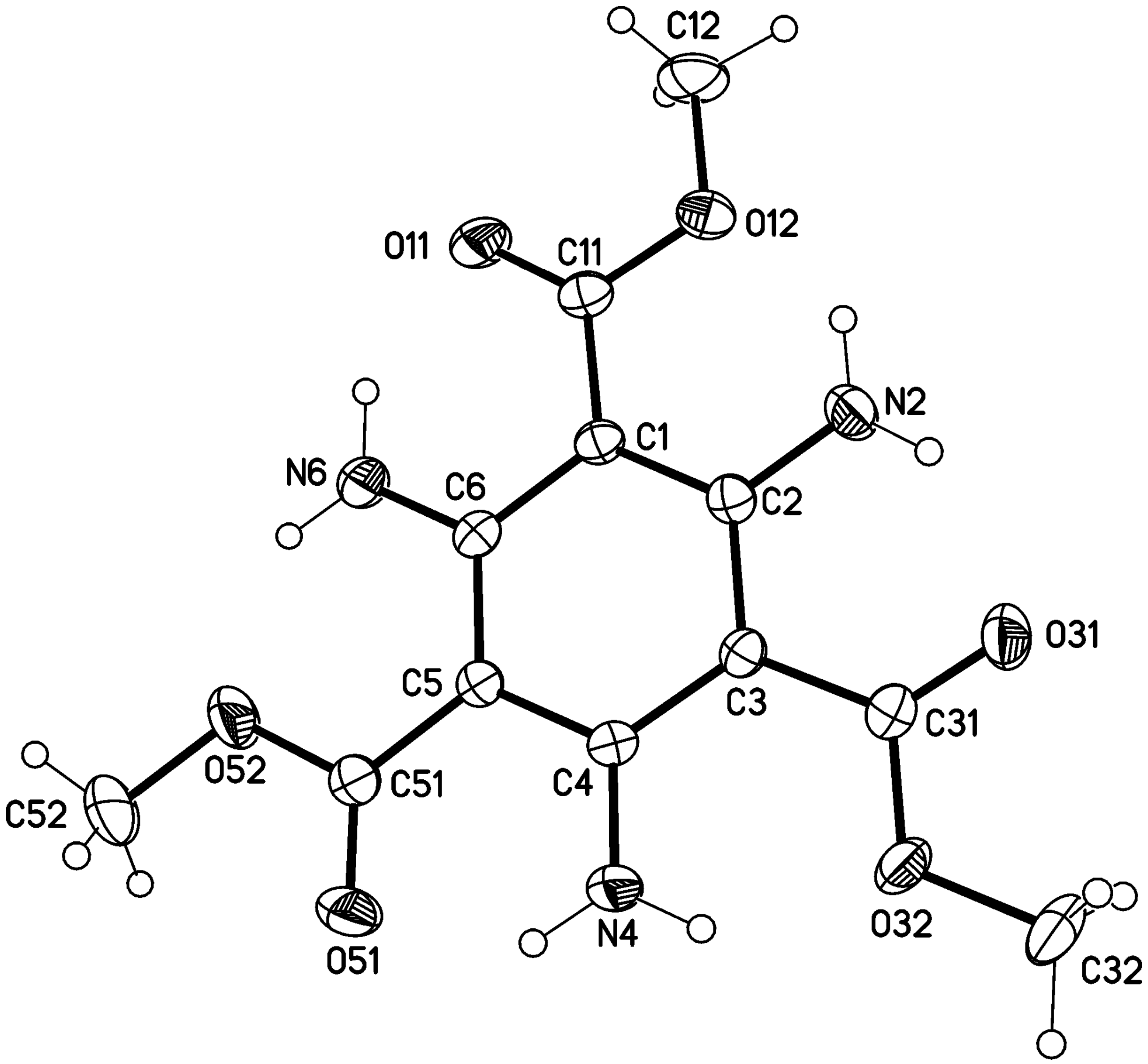

Figure S1. 1x. Molecular structure of **2a** (displacement parameters are drawn at 50 % probability level).

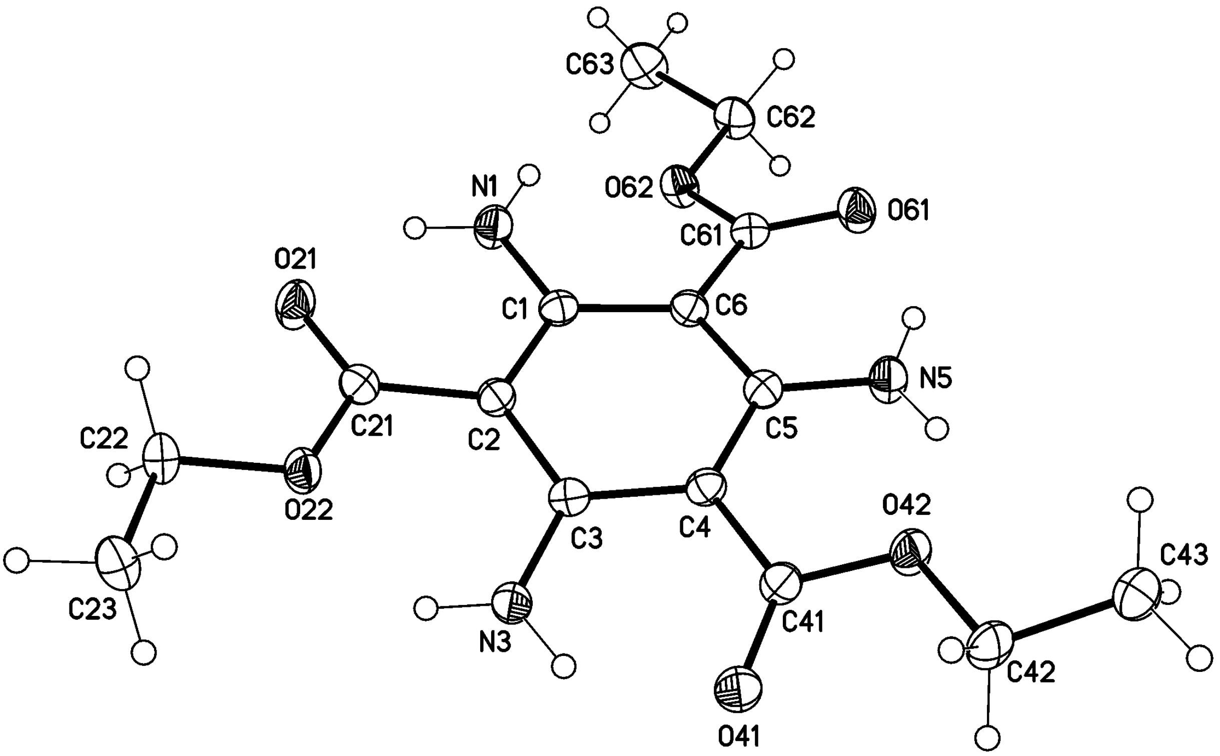

Figure S2. 2x. Molecular structure of **2b** (displacement parameters are drawn at 50 % probability level).

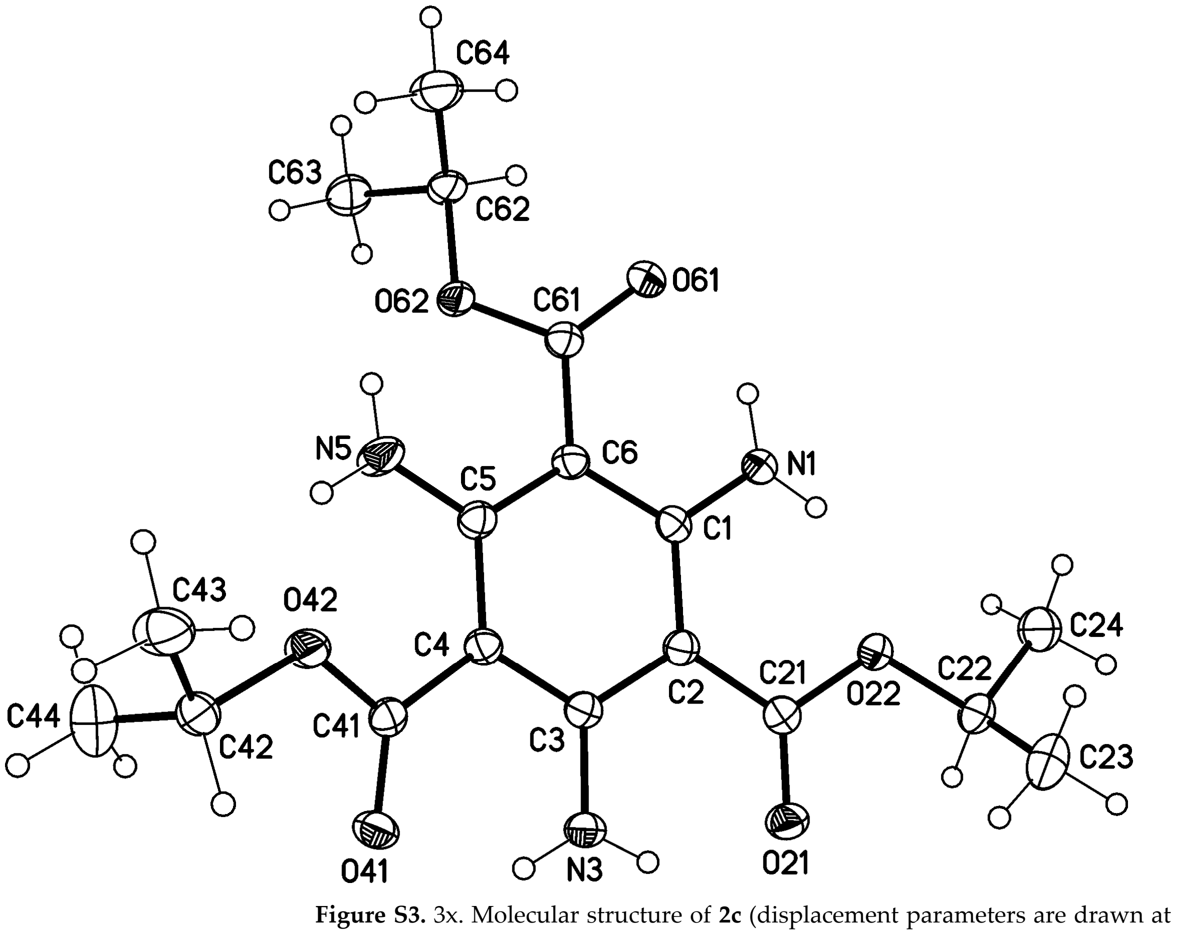

Figure S3. 3x. Molecular structure of **2c** (displacement parameters are drawn at 50 % probability level).

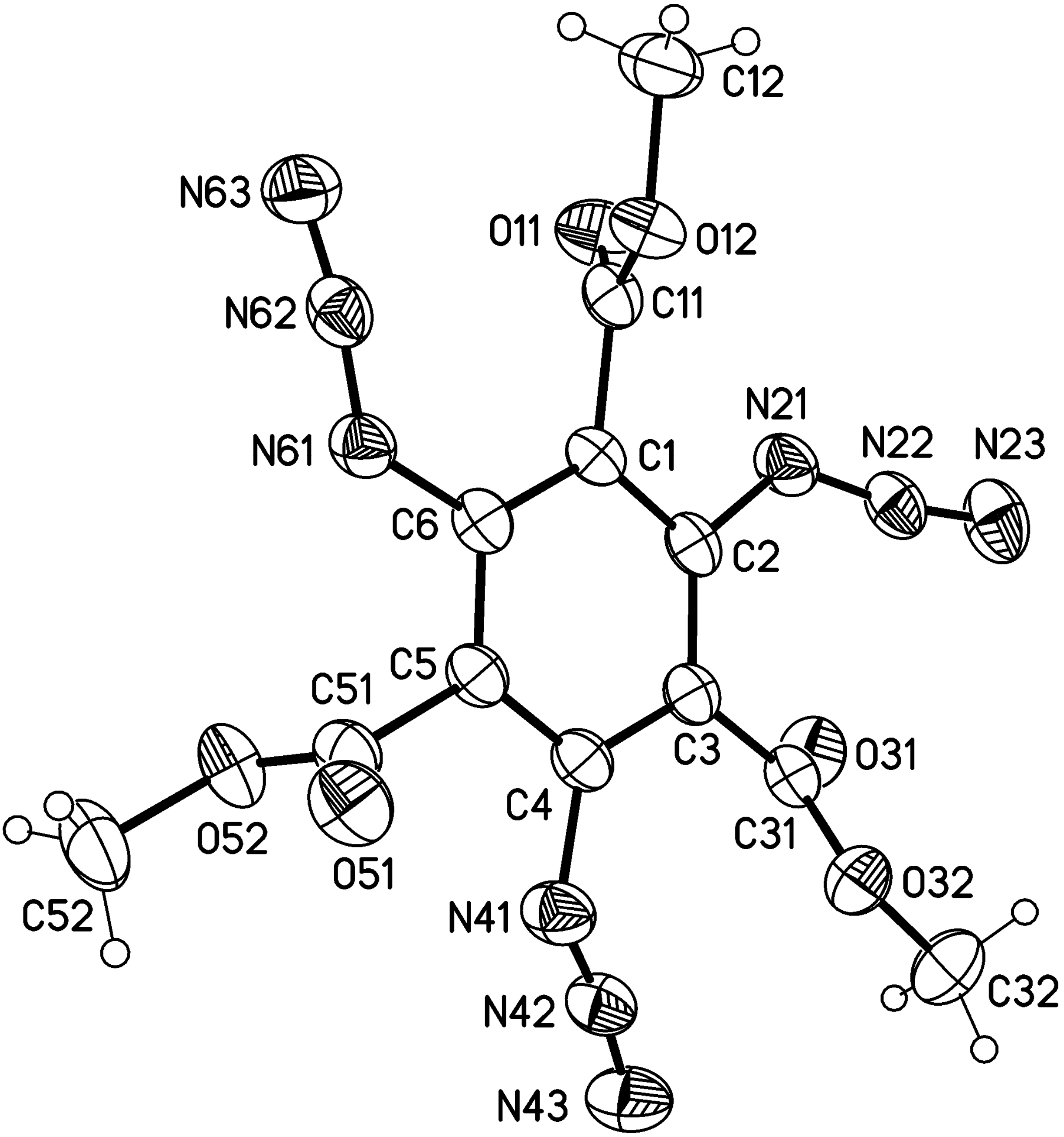

Figure S4. 4x. Molecular structure of **3a** (displacement parameters are drawn at 30 % probability level).

5. H- and ^{13}C-NMR spectra

Compound ***2a***

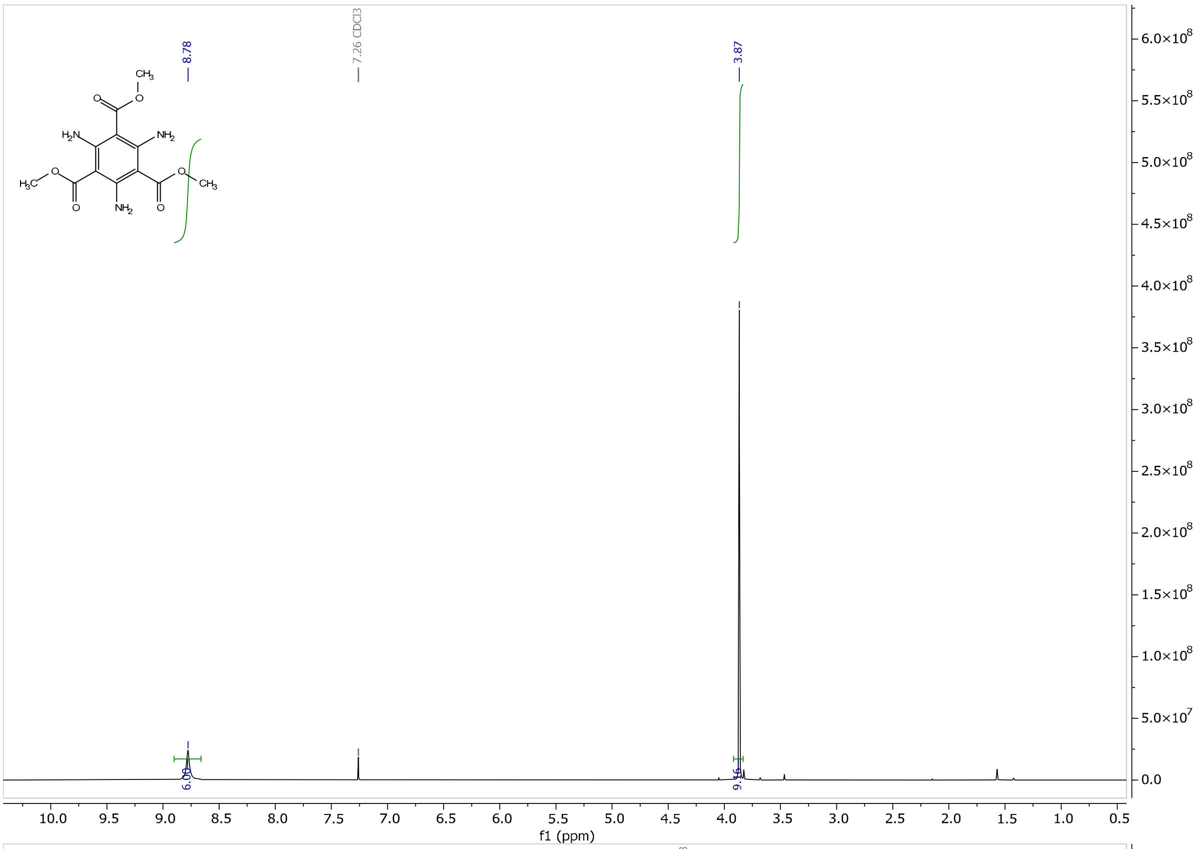

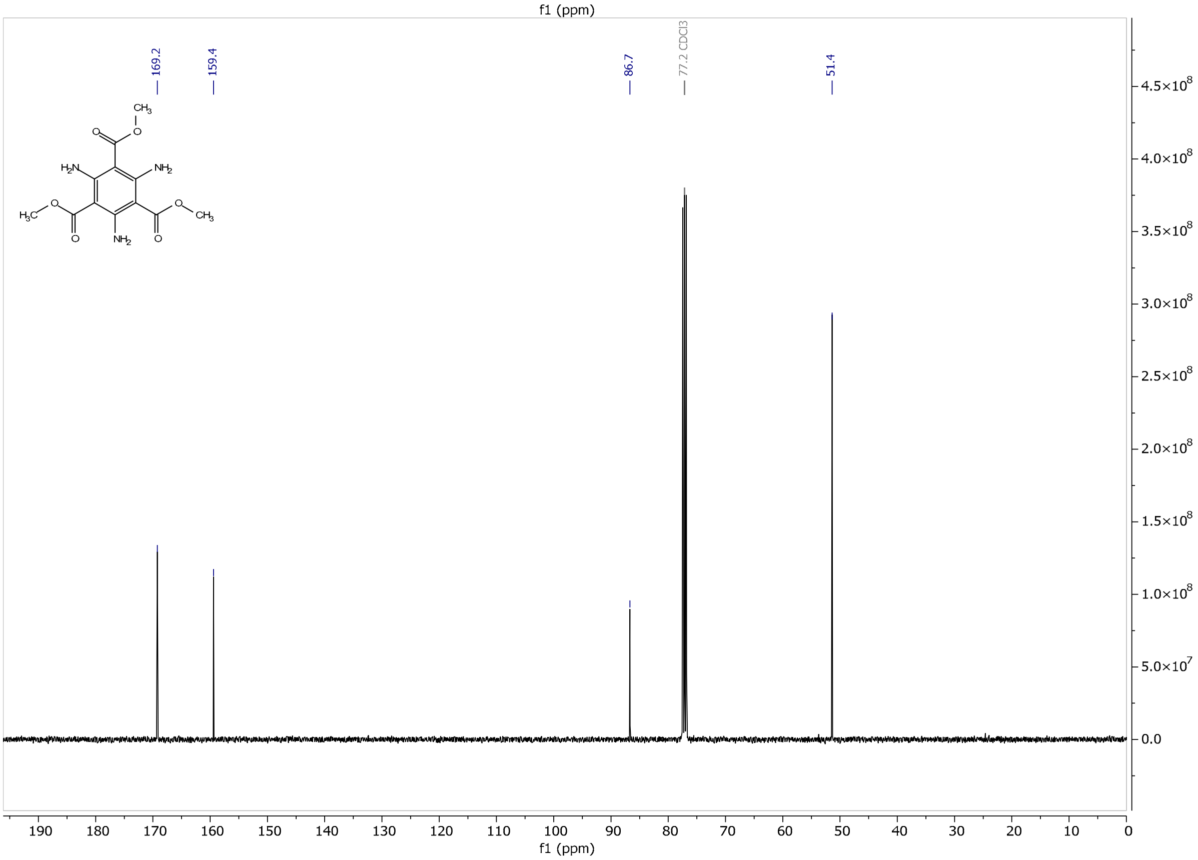

Compound ***2b***

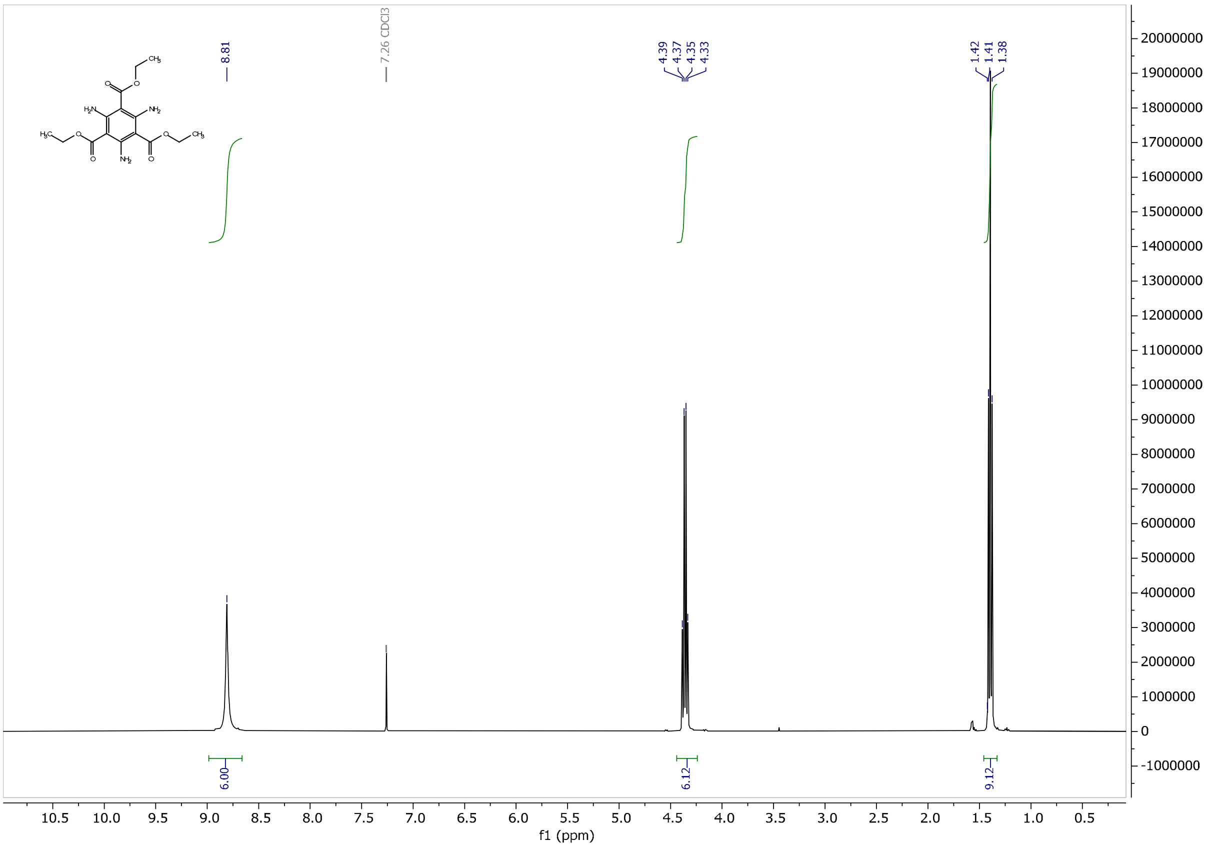
8.81
7.26 CDCl3
4.39
4.37
4.35
4.33
1.42
1.41
1.38
6.00
6.12
9.12
f1 (ppm)

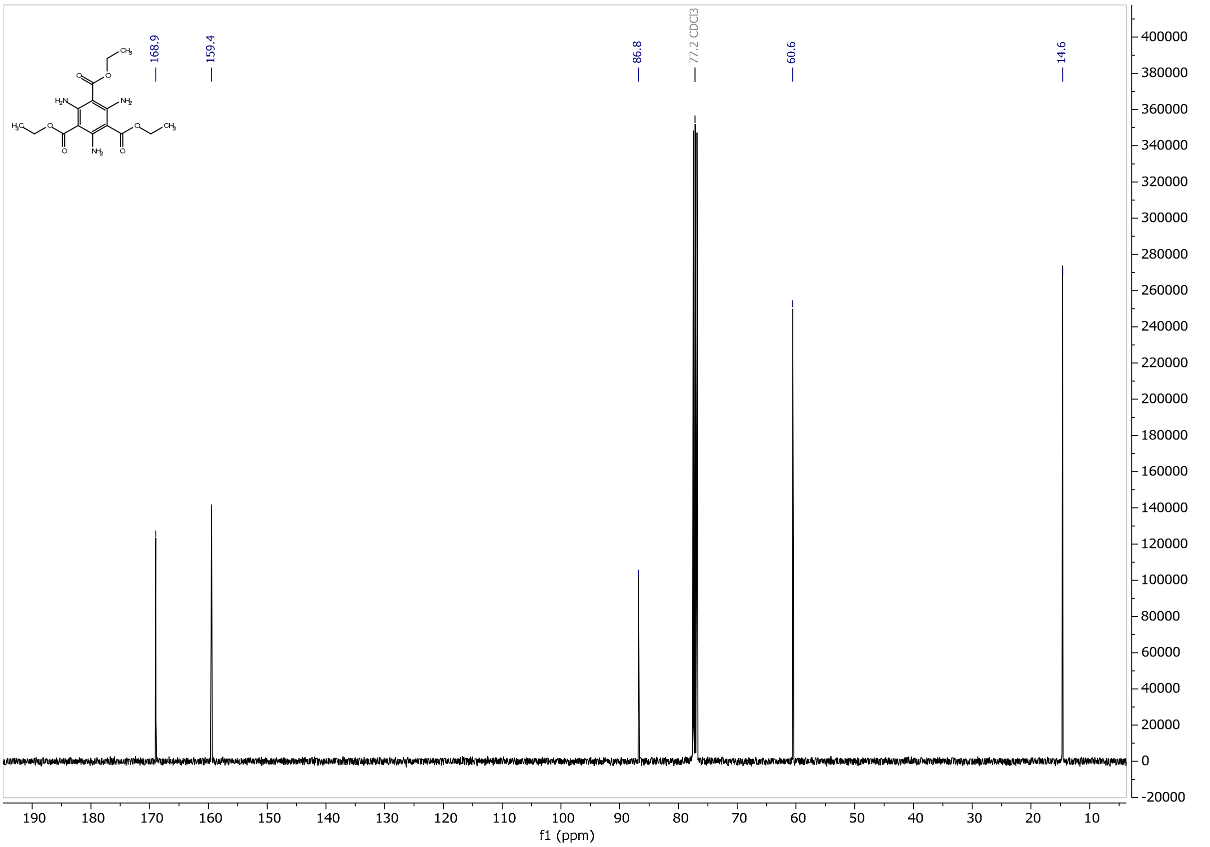
168.9
159.4
86.8
77.2 CDCl3
60.6
14.6
f1 (ppm)

Compound ***2c***

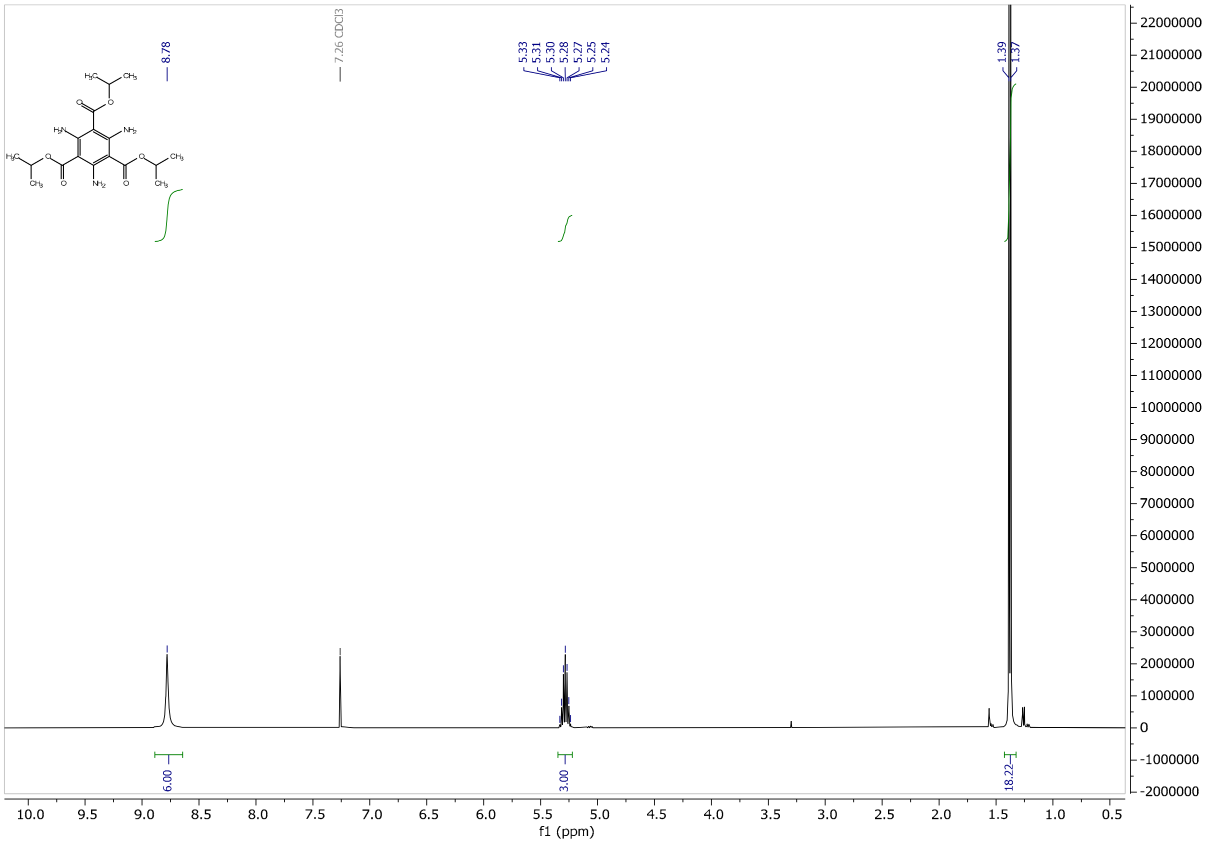
8.78
7.26 CDCl3
5.33
5.31
5.30
5.28
5.27
5.25
5.24
1.39
1.37
6.00
3.00
18.22
f1 (ppm)

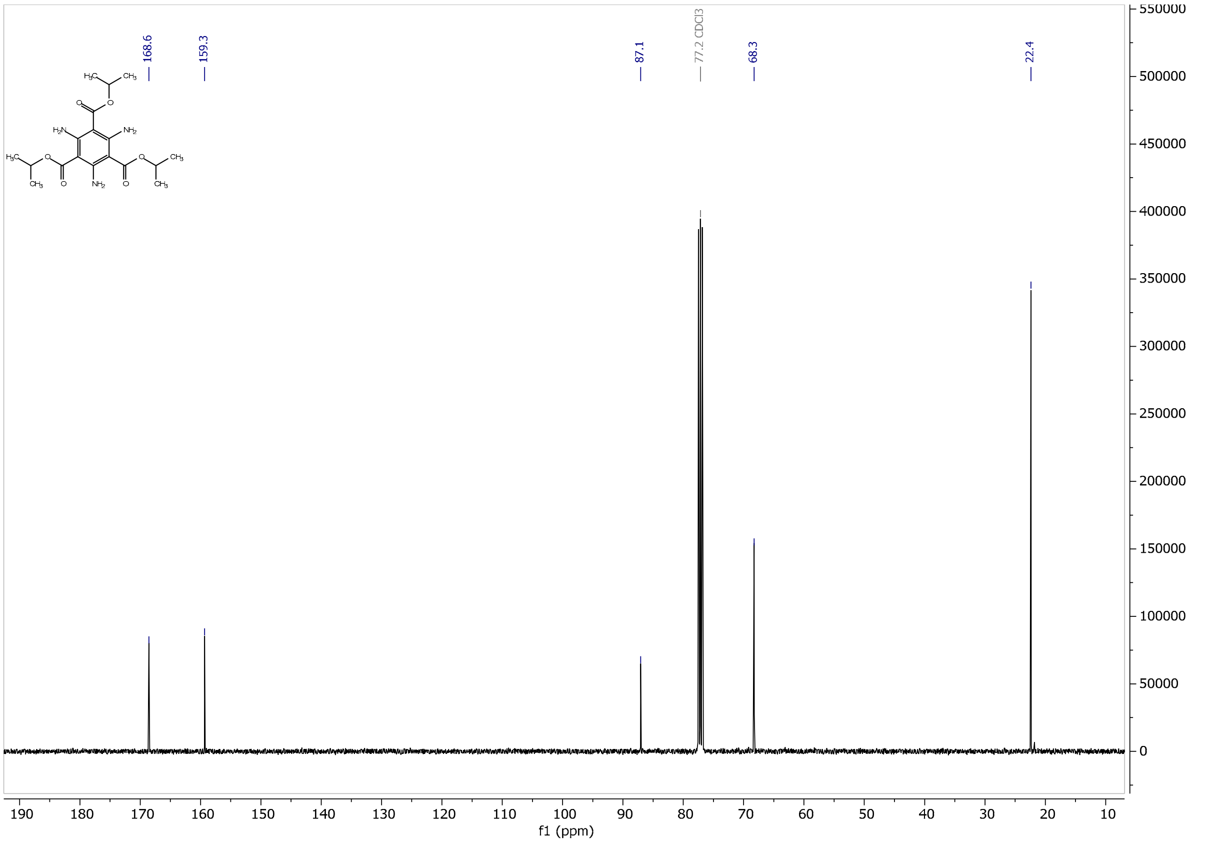
168.6
159.3
87.1
77.2 CDCl3
68.3
22.4
f1 (ppm)

Compound ***2d***

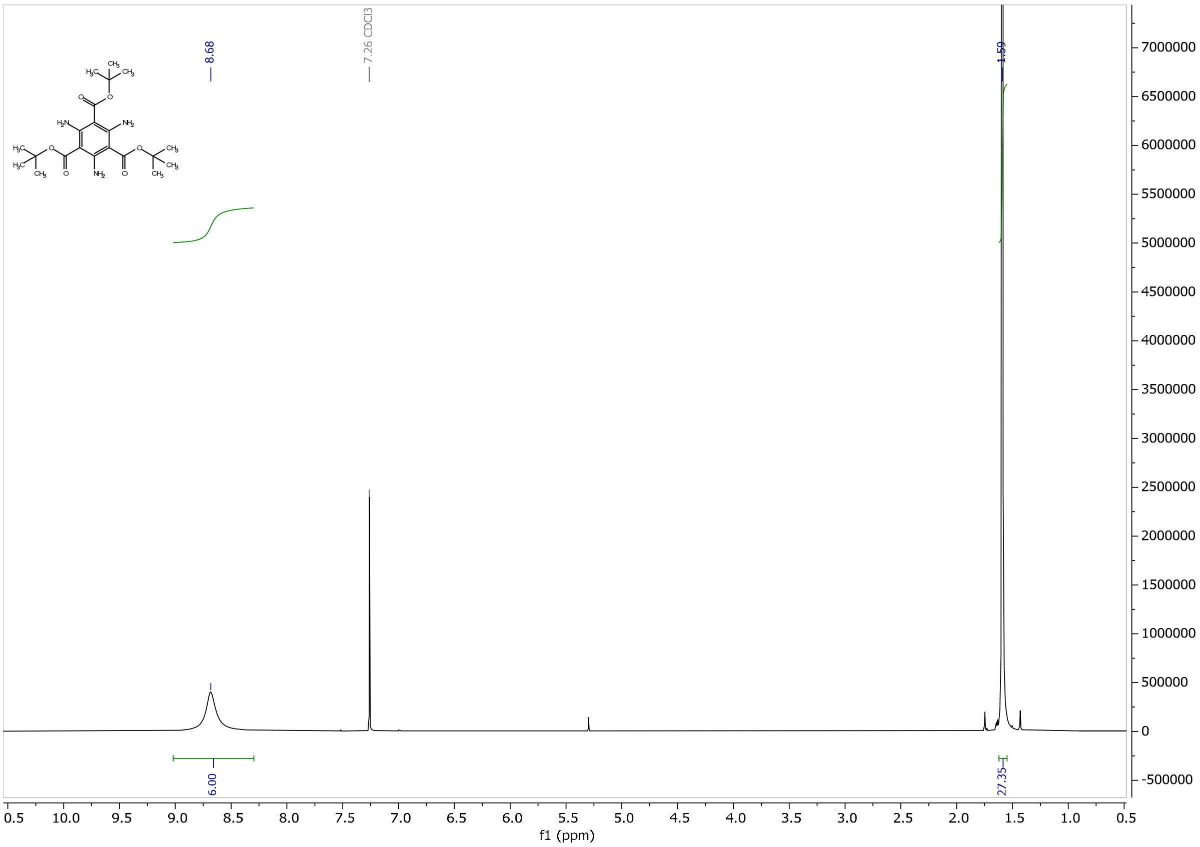
8.68
7.26 CDCl3
1.59
6.00
27.35
f1 (ppm)

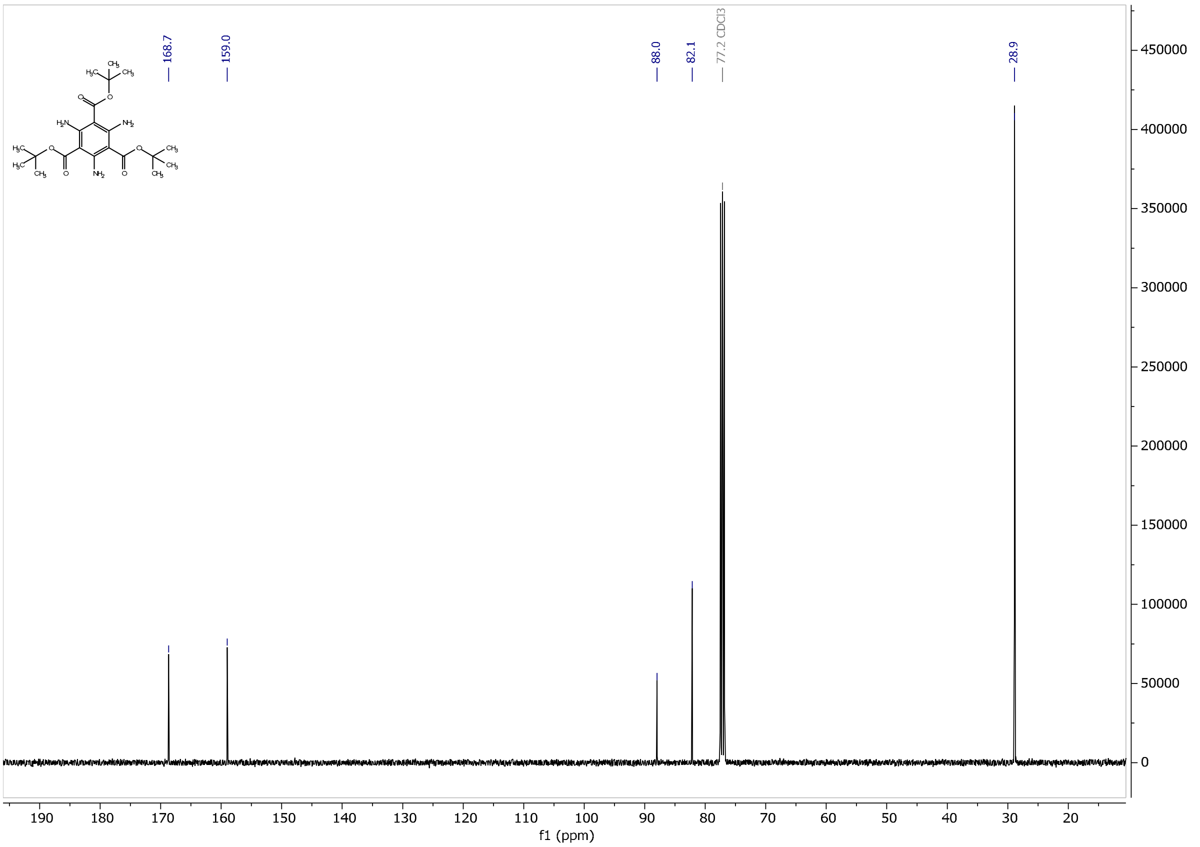
168.7
159.0
88.0
82.1
77.2 CDCl3
28.9
f1 (ppm)

*Compound **2e***

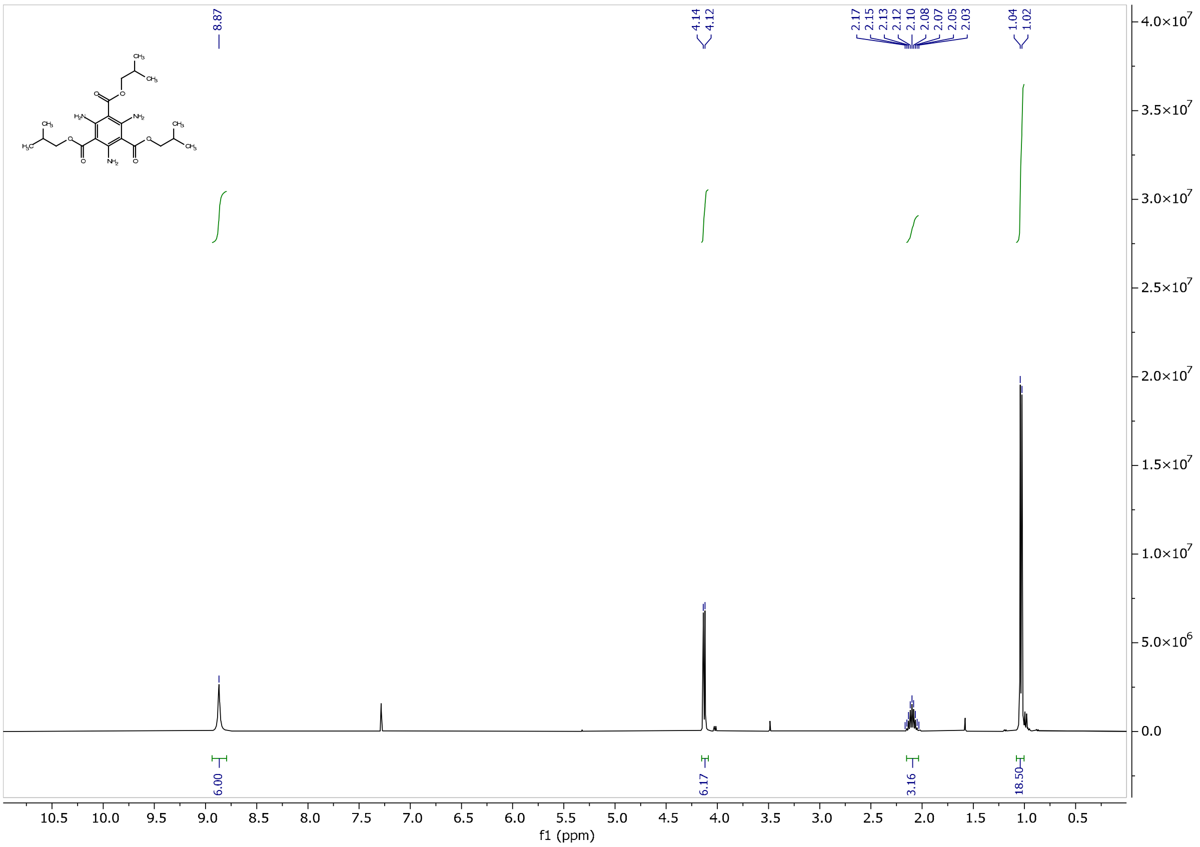
8.87
4.14
4.12
2.17
2.15
2.13
2.12
2.10
2.08
2.07
2.05
2.03
1.04
1.02
6.00
6.17
3.16
18.50
f1 (ppm)

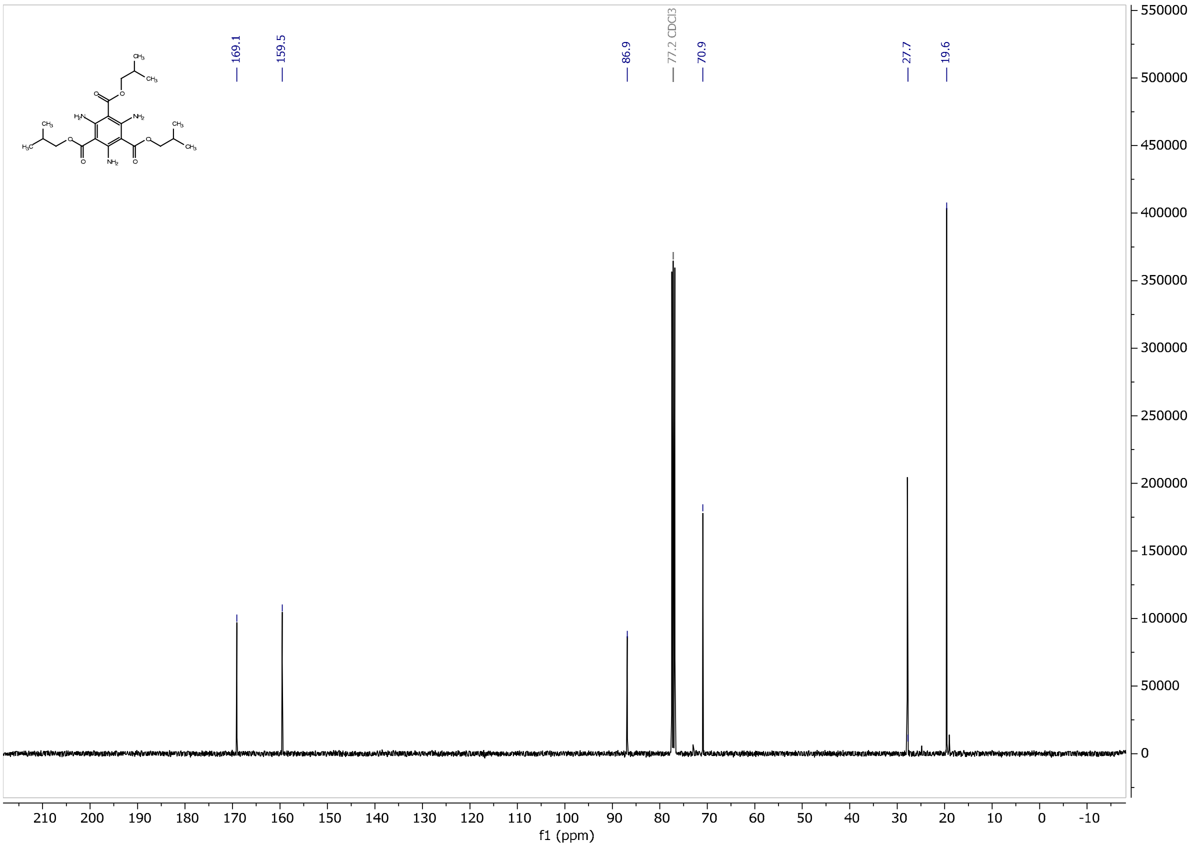
169.1
159.5
86.9
77.2 CDCl3
70.9
27.7
19.6
f1 (ppm)

Compound ***2f***

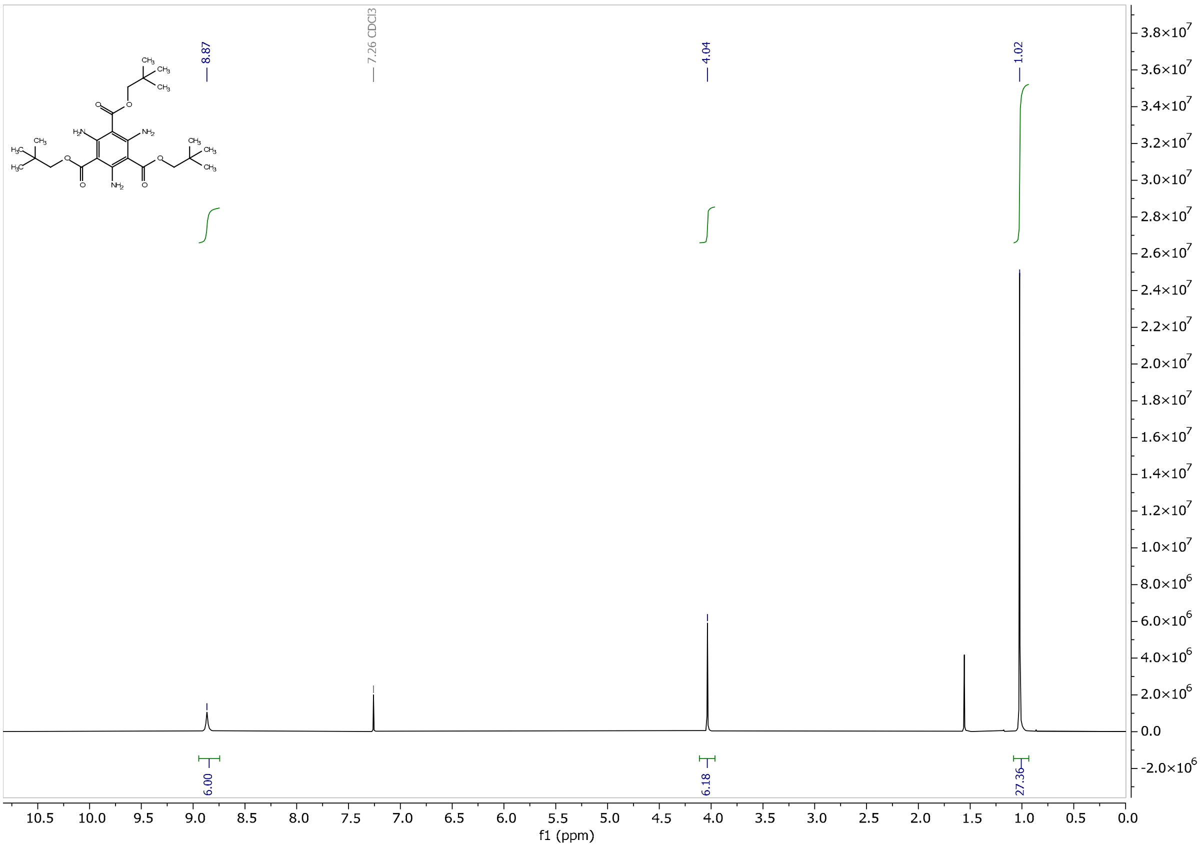
8.87
7.26 CDCl3
4.04
1.02
6.00
6.18
27.36
f1 (ppm)

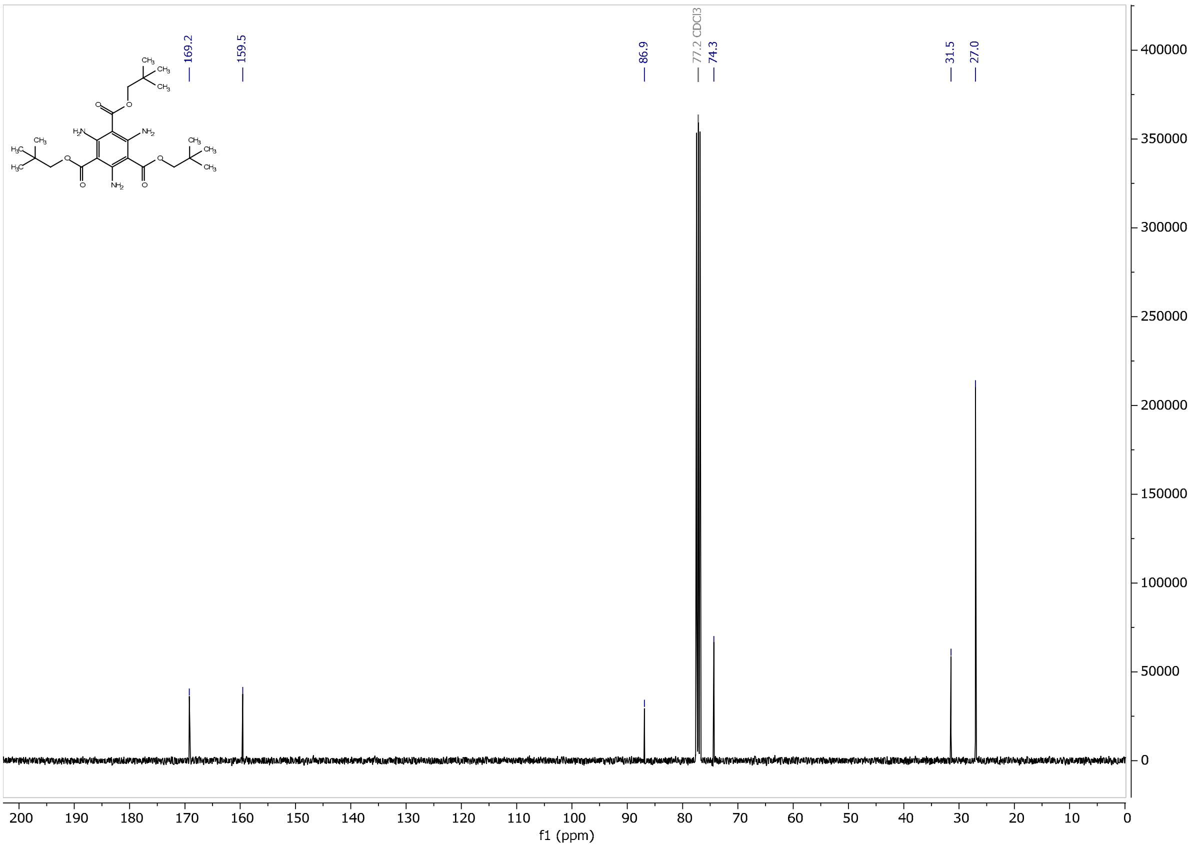
169.2
159.5
86.9
77.2 CDCl3
74.3
31.5
27.0
f1 (ppm)

Compound ***2g***

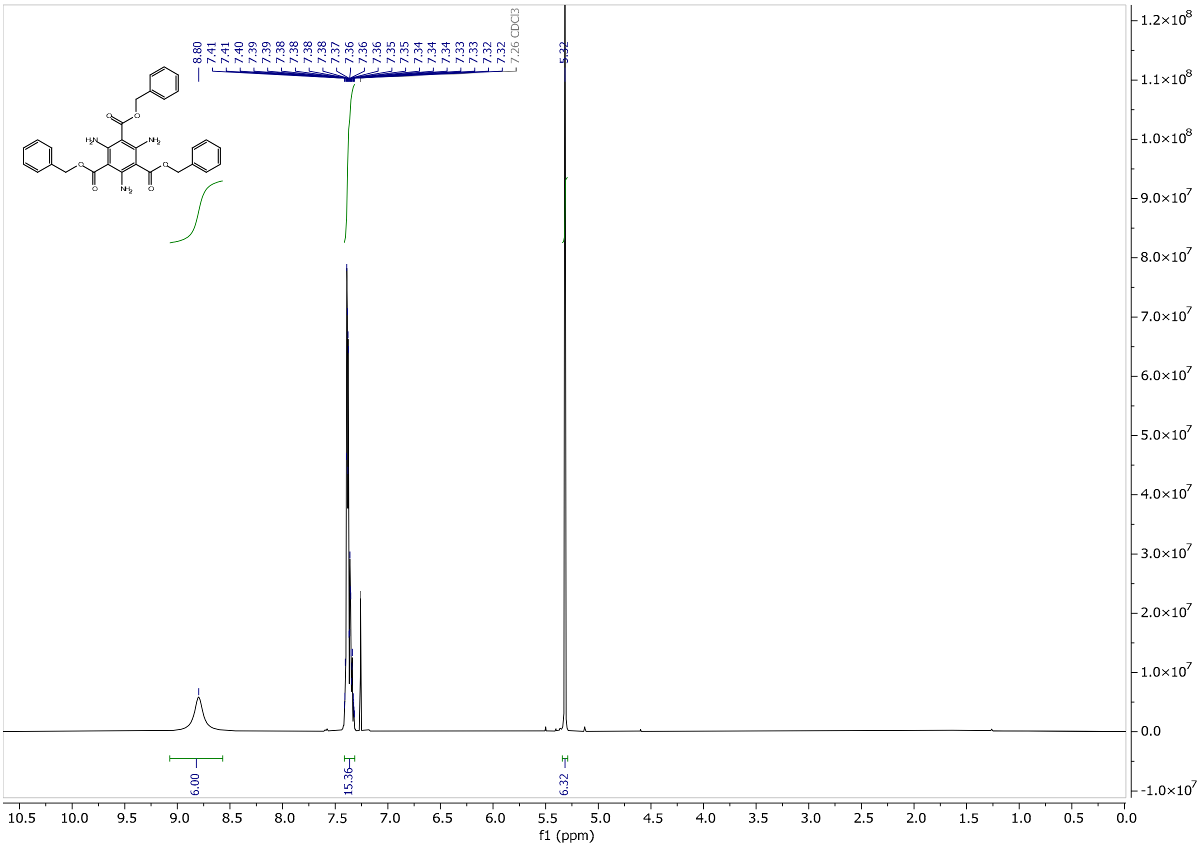
8.80
7.41
7.41
7.40
7.39
7.39
7.38
7.38
7.38
7.38
7.37
7.36
7.36
7.36
7.35
7.35
7.34
7.34
7.34
7.33
7.33
7.32
7.32
7.26 CDCl3
5.32
6.00
15.36
6.32
f1 (ppm)

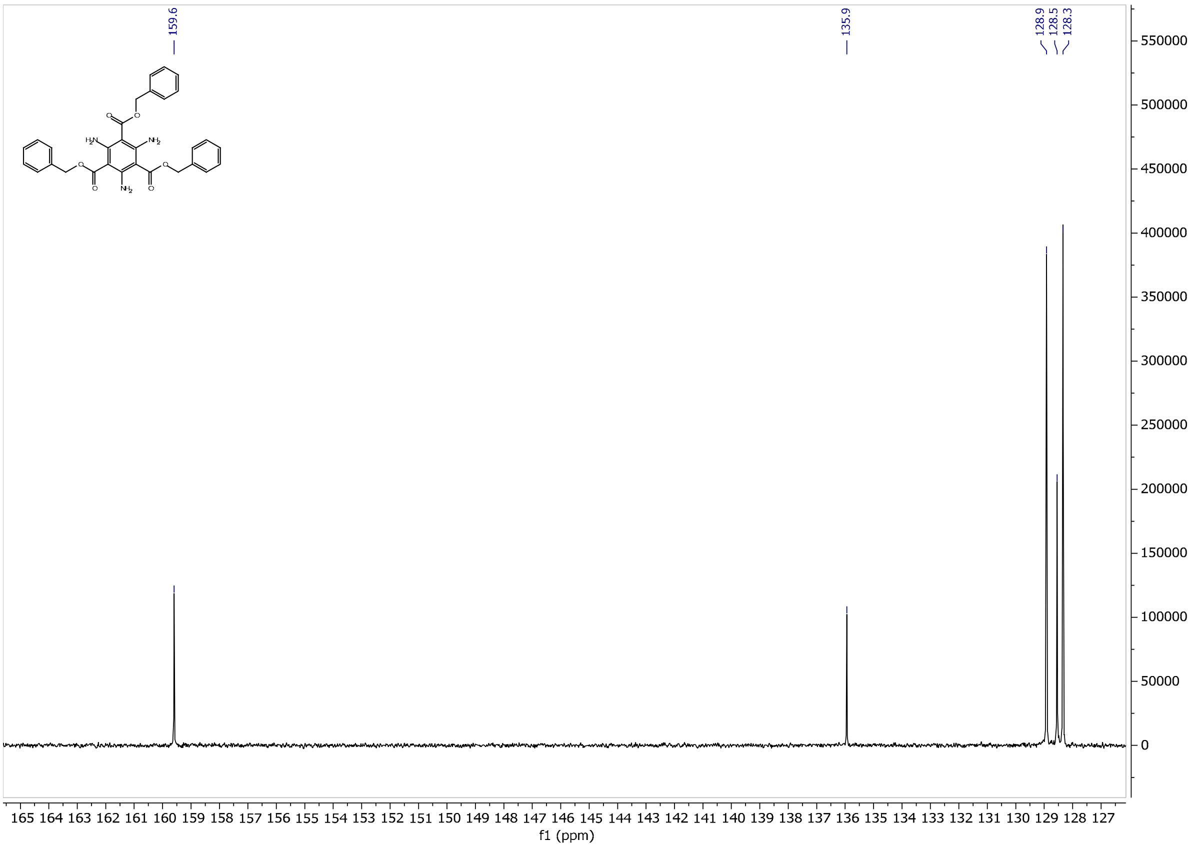
159.6
135.9
128.9
128.5
128.3
f1 (ppm)

*Compound **3a***

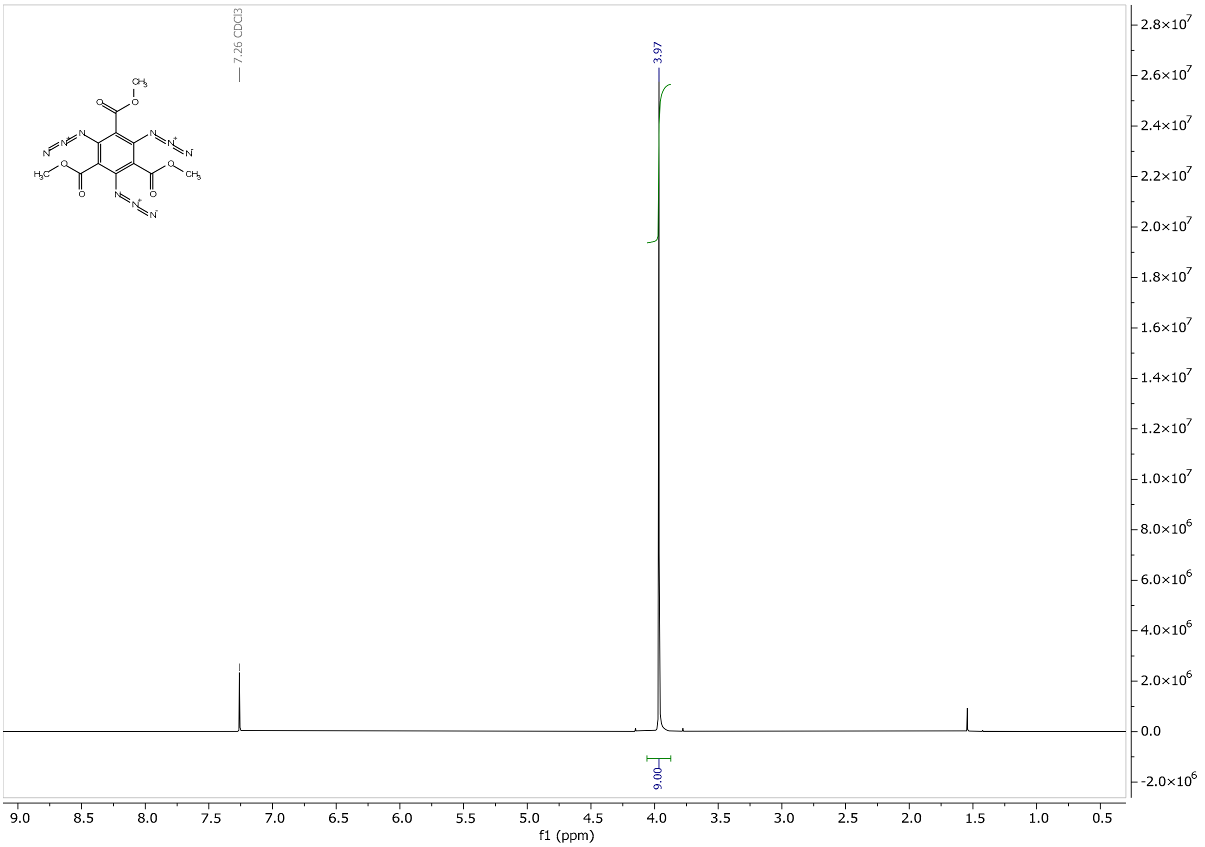

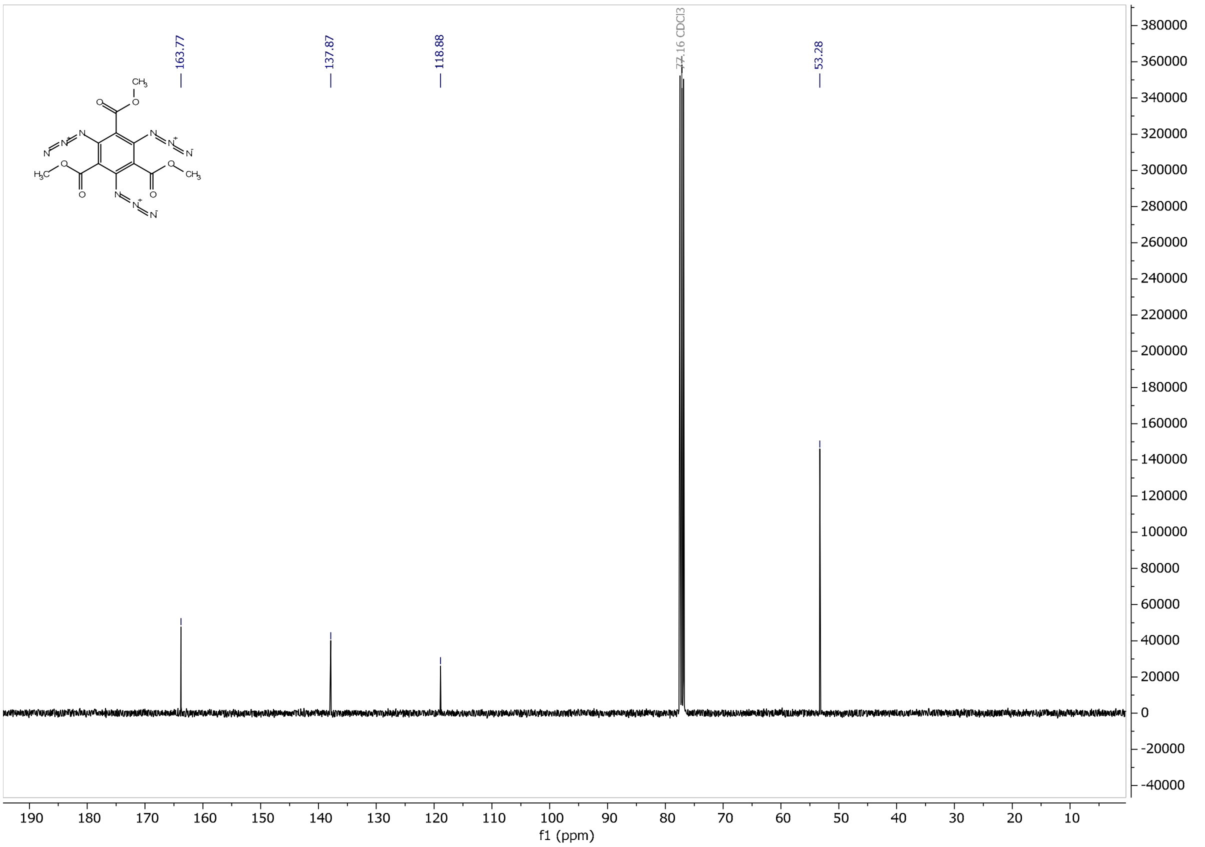

Compound ***3b***

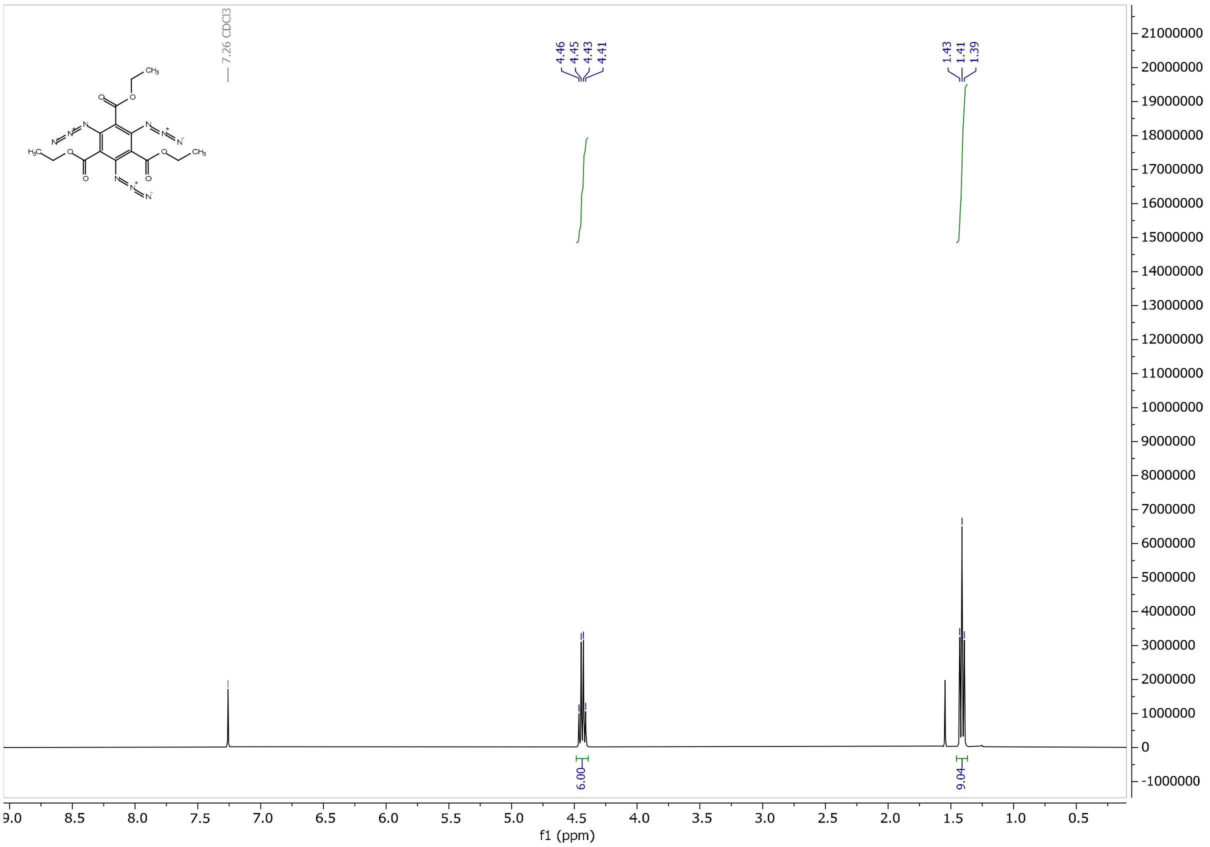
7.26 CDCl3
4.46
4.45
4.43
4.41
1.43
1.41
1.39
6.00
9.04
f1 (ppm)

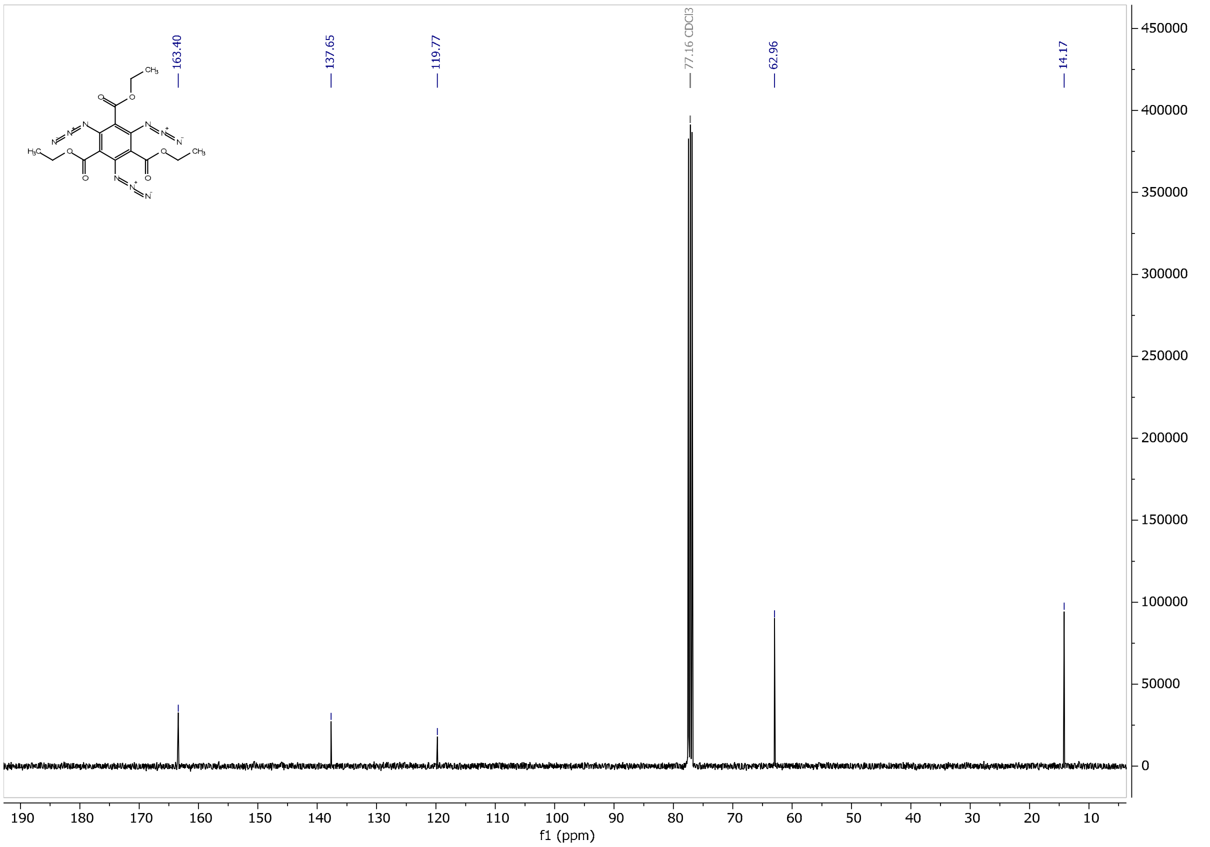
163.40
137.65
119.77
77.16 CDCl3
62.96
14.17
f1 (ppm)

Compound ***3c***

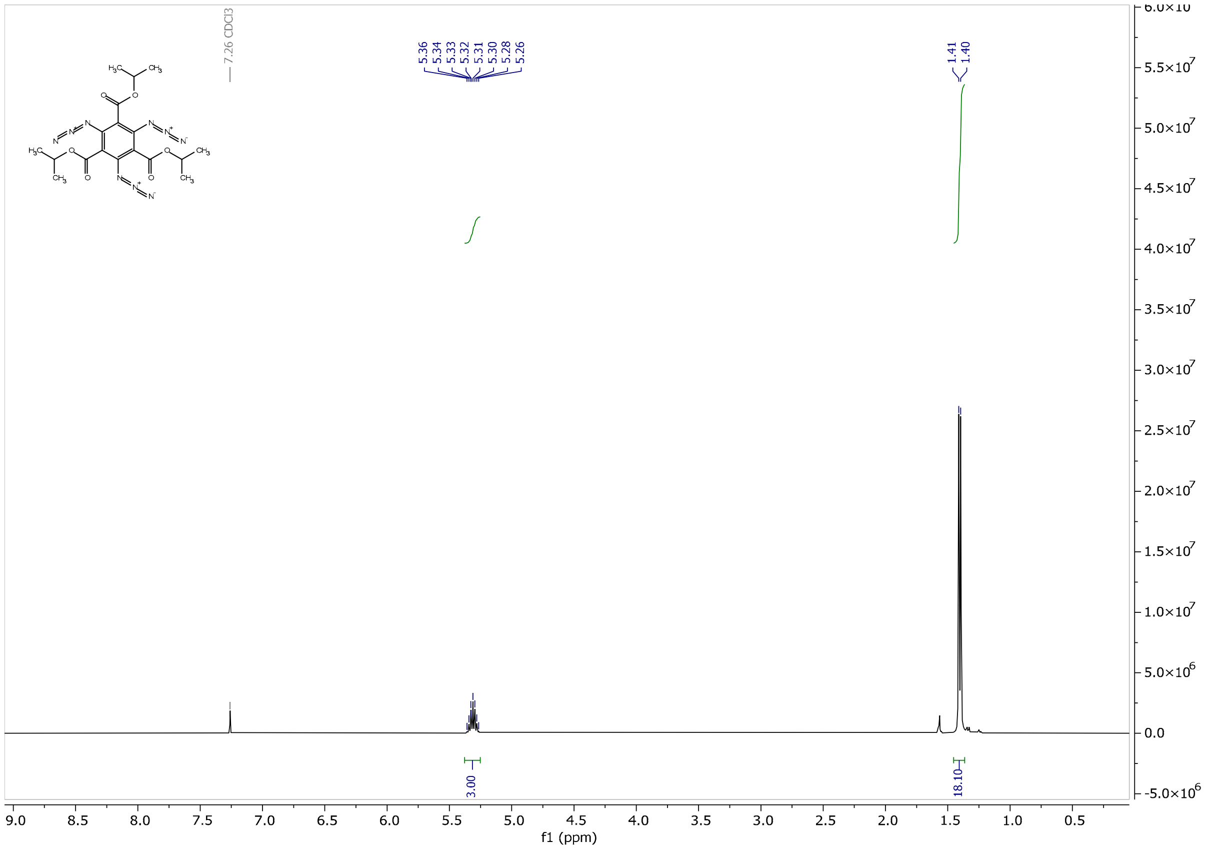

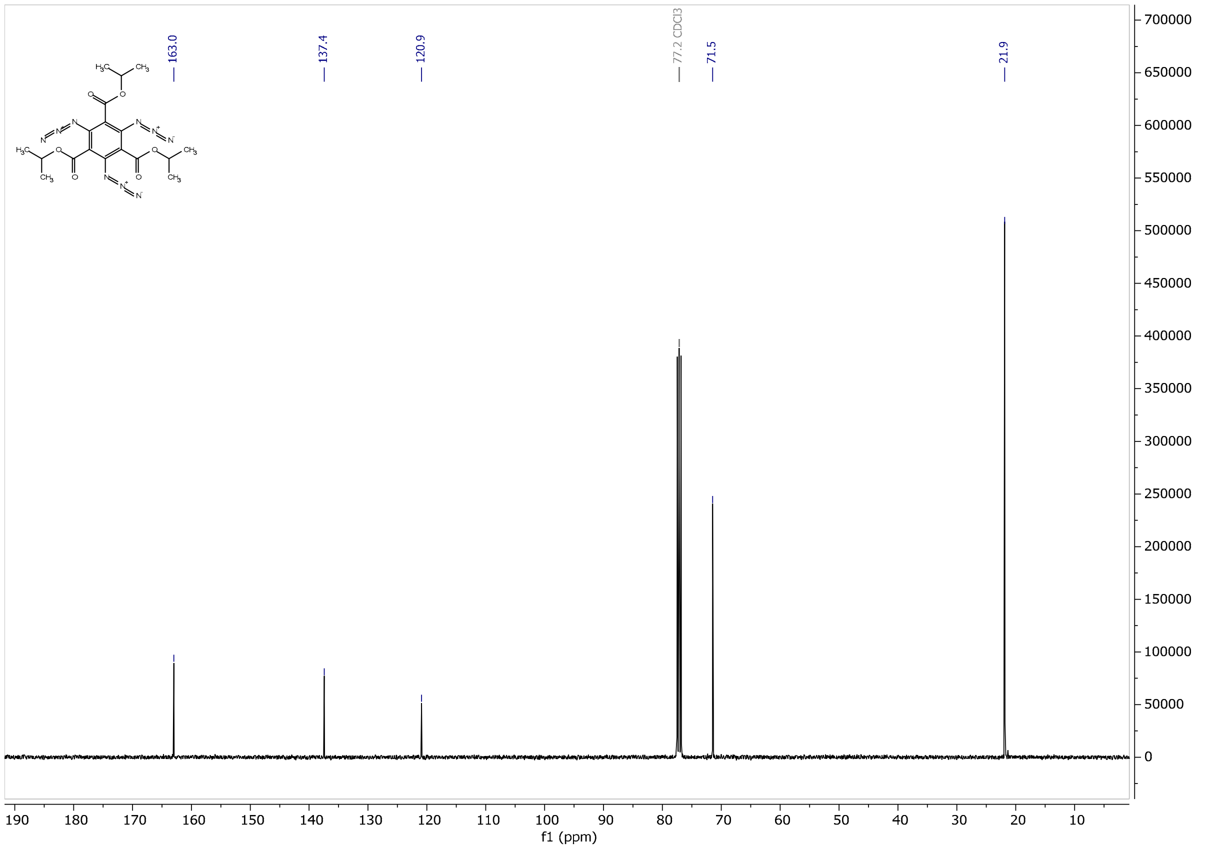

Compound ***3g***

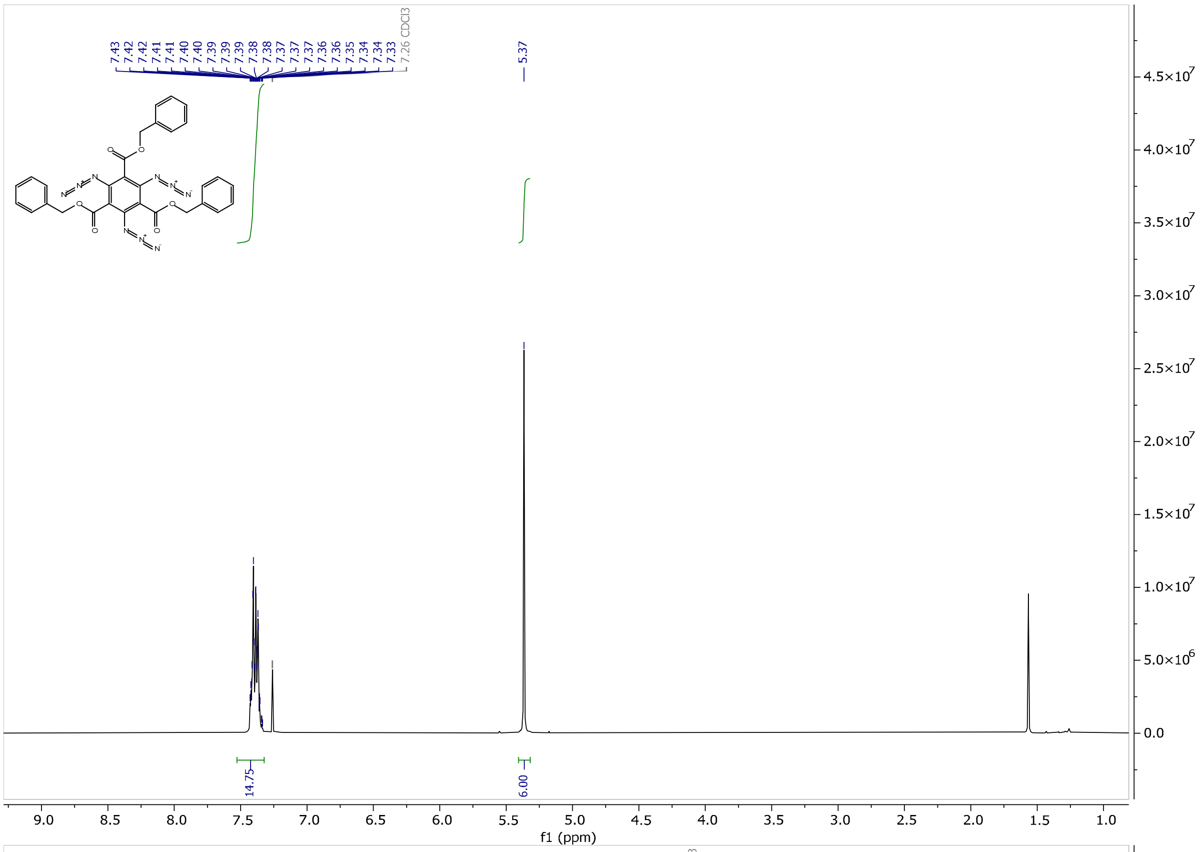
7.43
7.42
7.42
7.41
7.41
7.40
7.40
7.39
7.39
7.39
7.39
7.38
7.38
7.37
7.37
7.37
7.36
7.36
7.35
7.34
7.34
7.33
7.26 CDCl3
5.37
14.75
6.00
f1 (ppm)

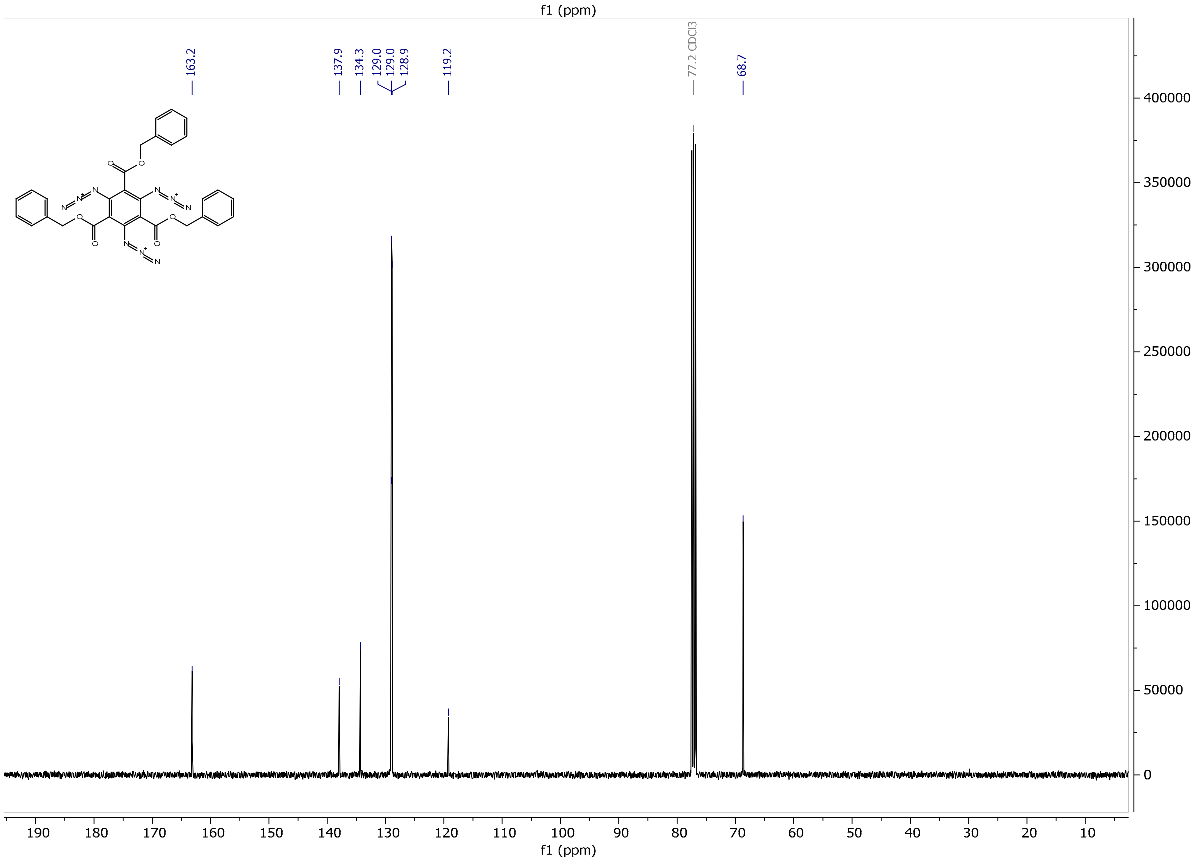
163.2
137.9
134.3
129.0
129.0
128.9
119.2
77.2 CDCl3
68.7
f1 (ppm)

Compound ***4***

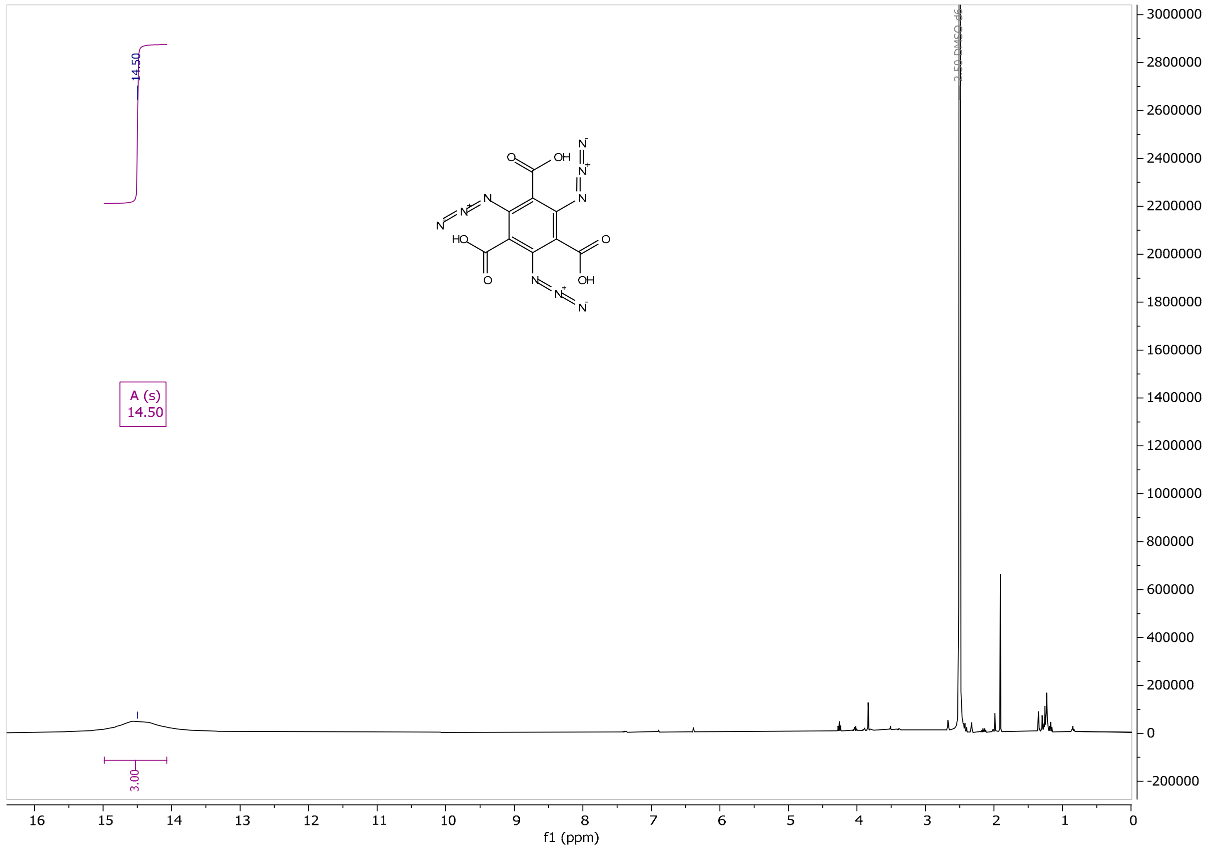
14.50
A (s)
14.50
3.00
3.50 DMSO-d6
f1 (ppm)

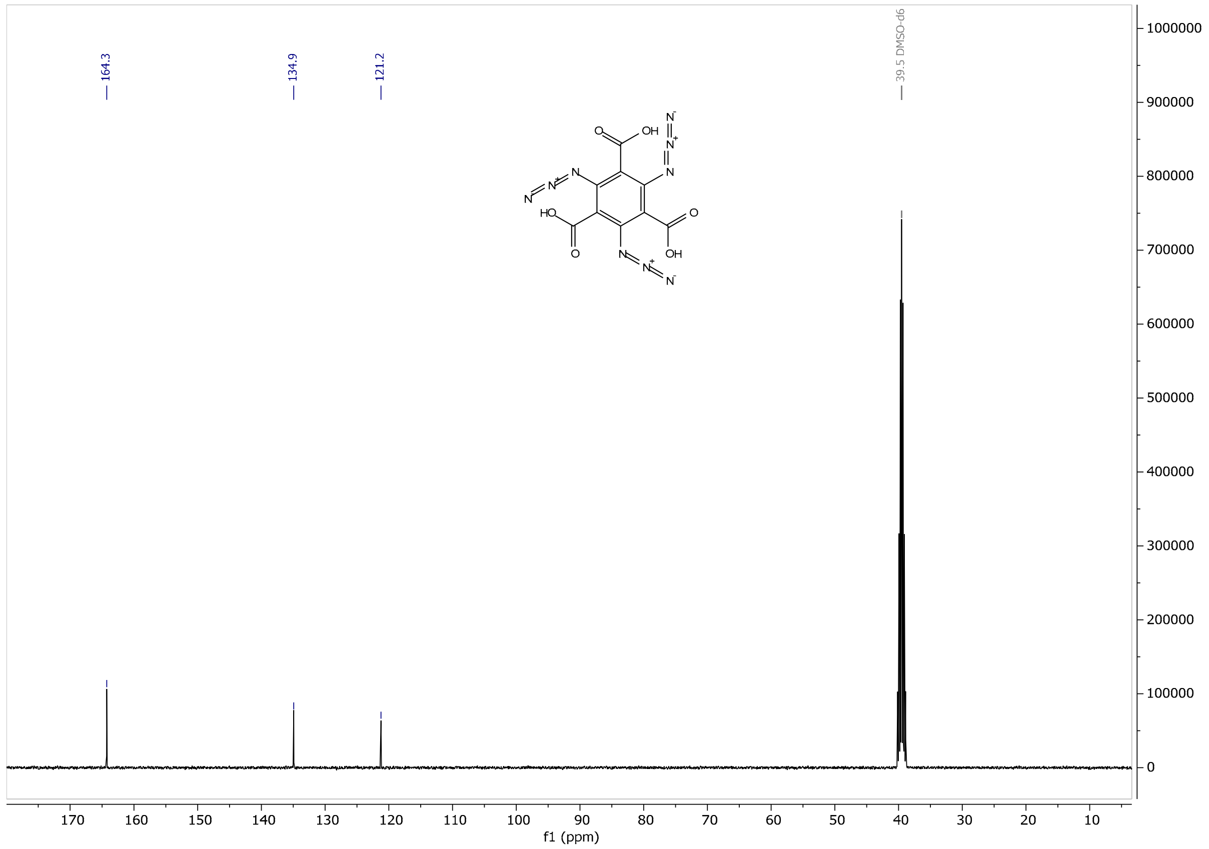
164.3
134.9
121.2
39.5 DMSO-d6
f1 (ppm)

Compound **5**

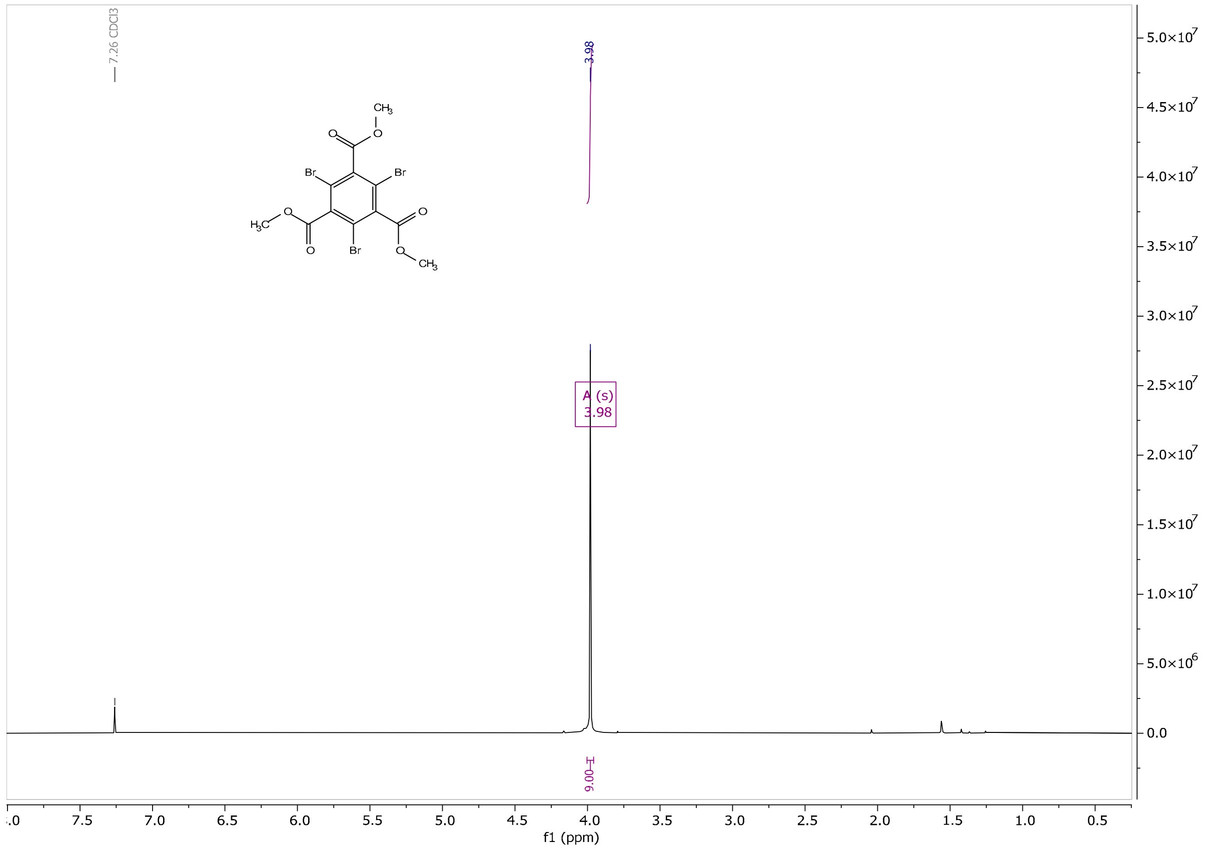
7.26 CDCl3
3.98
A (s)
3.98
9.00
f1 (ppm)

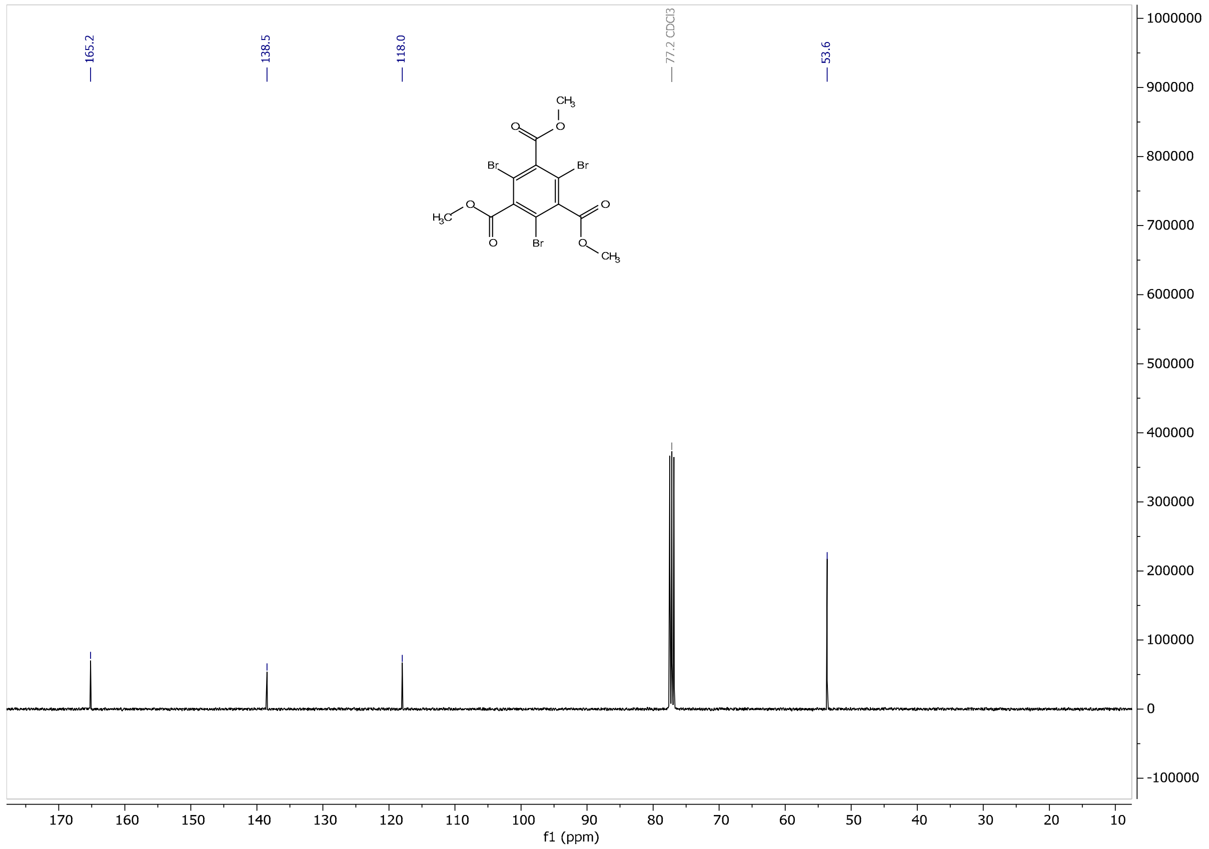
165.2
138.5
118.0
77.2 CDCl3
53.6
f1 (ppm)

Compound ***6a-Ph***

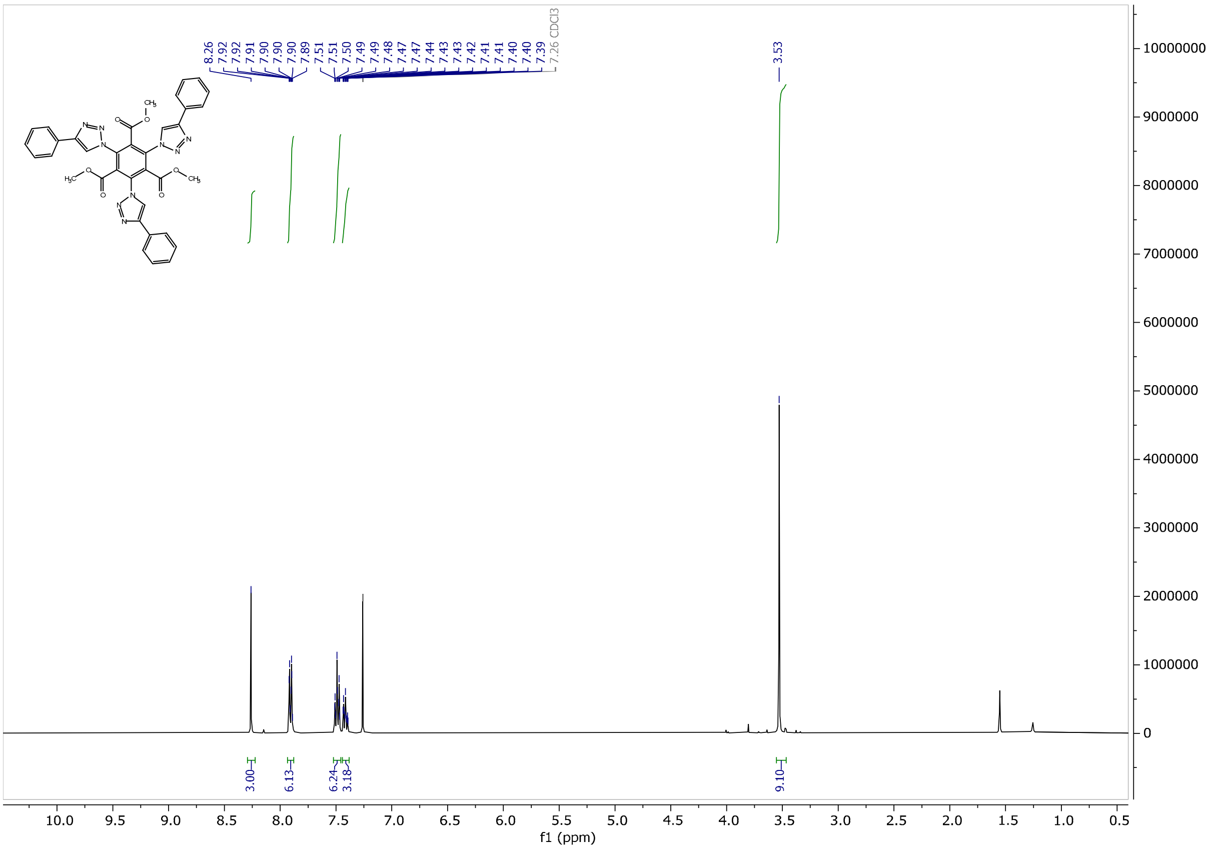

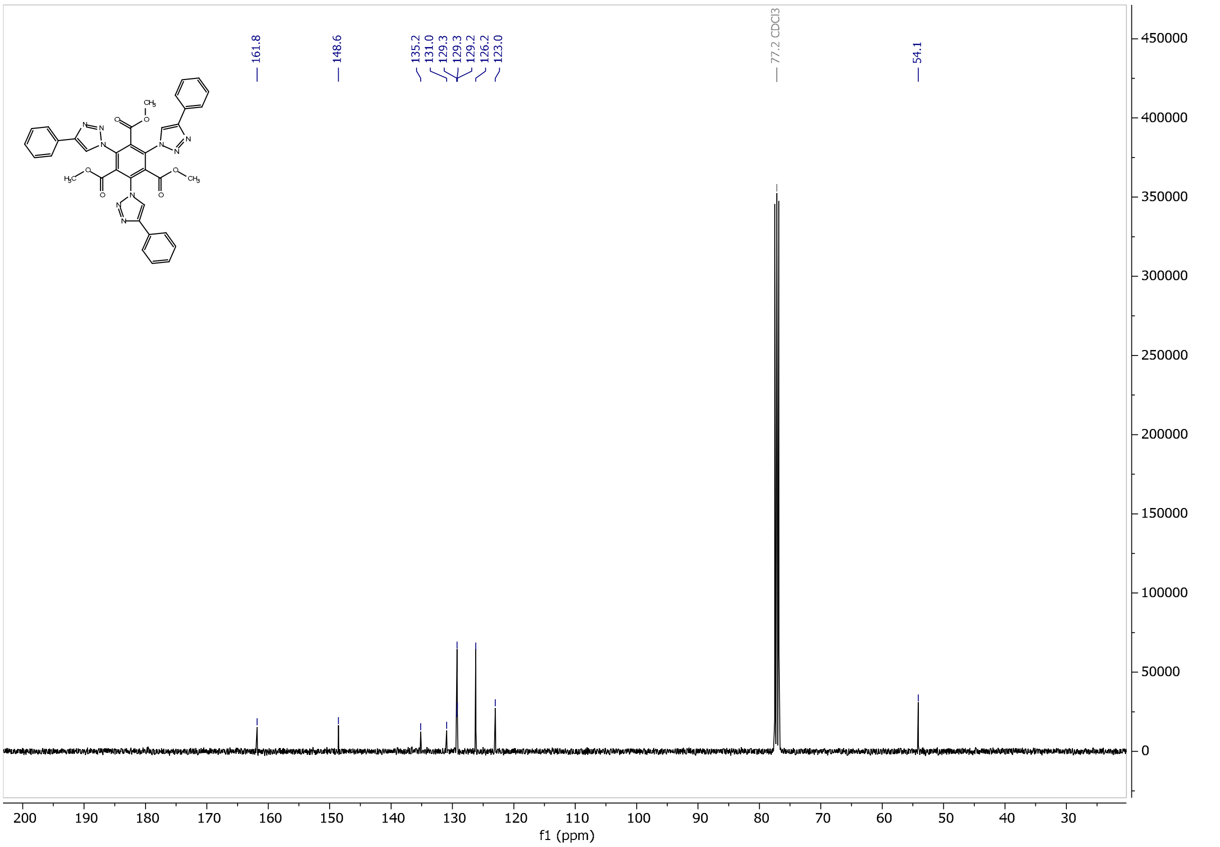

Compound ***6a-C₆H₄Br***

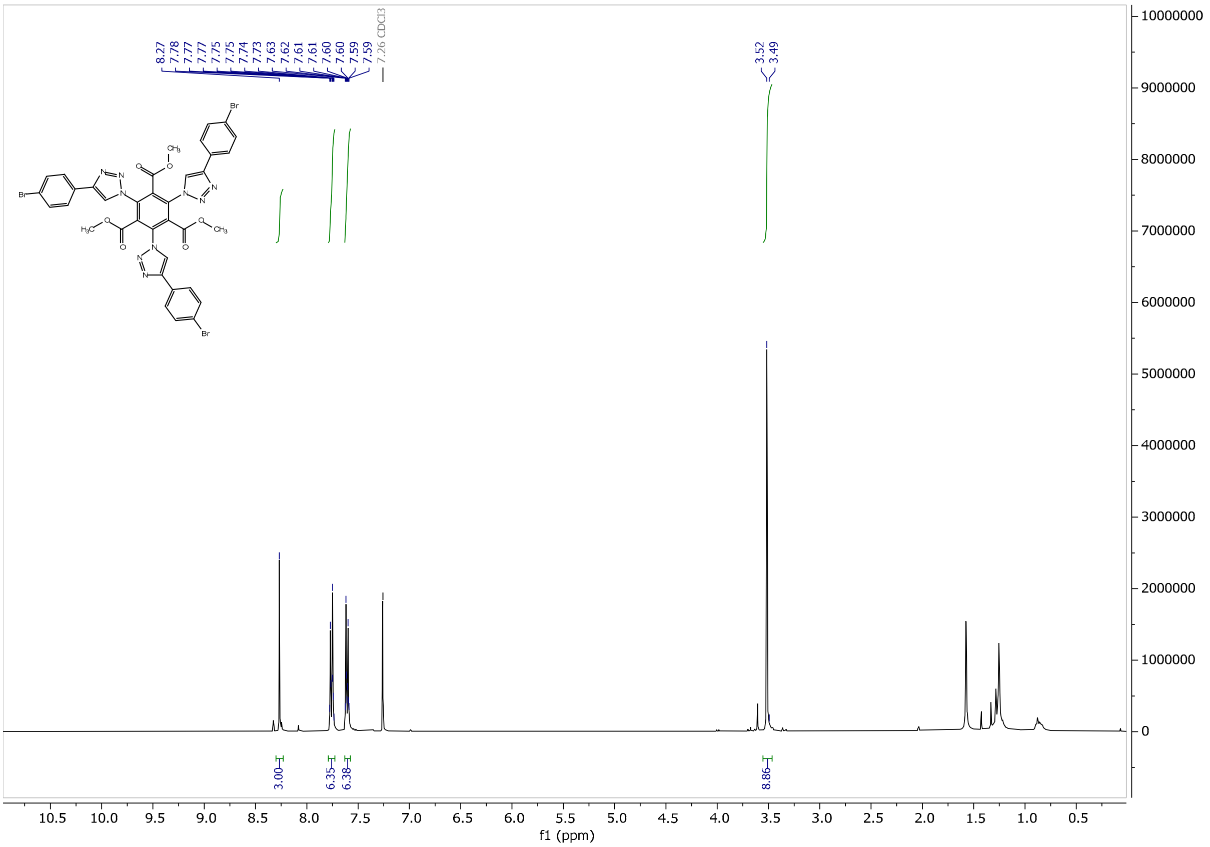

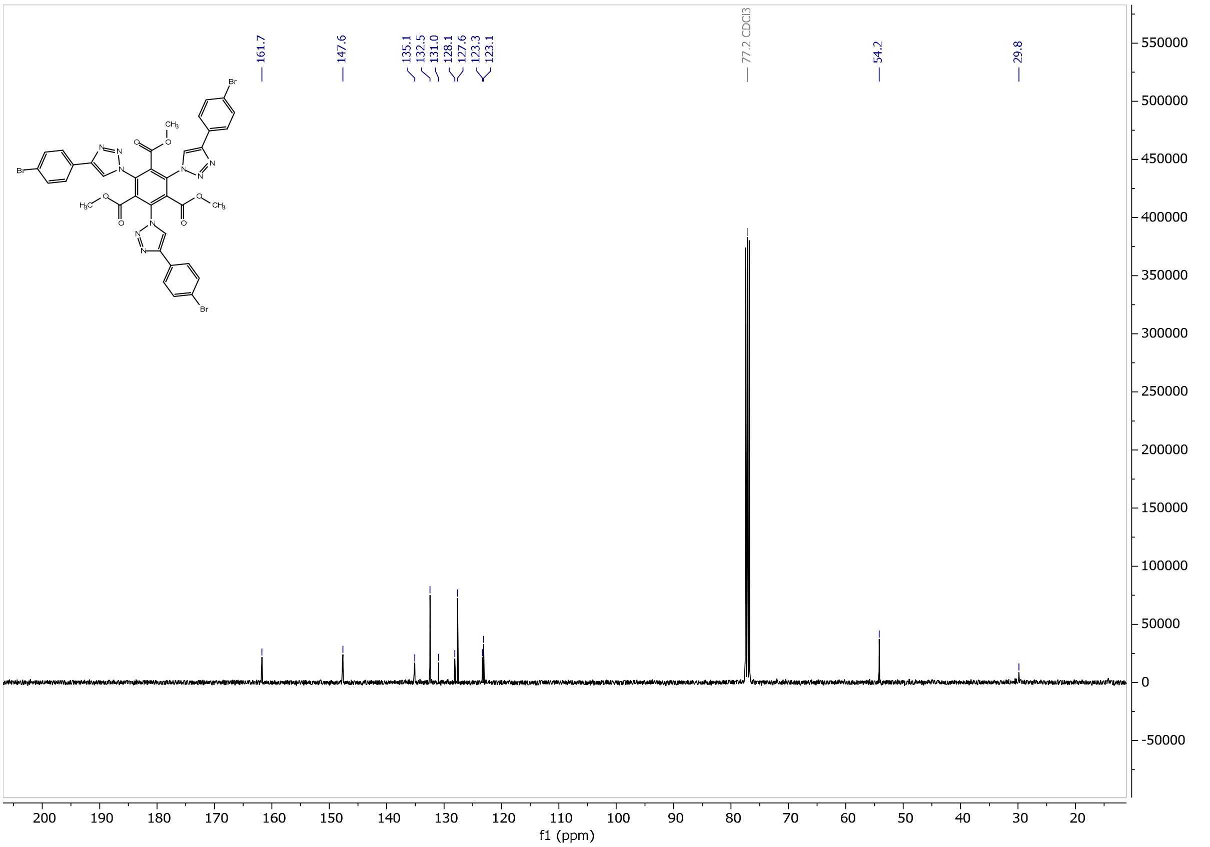

Compound ***6b-Ph***

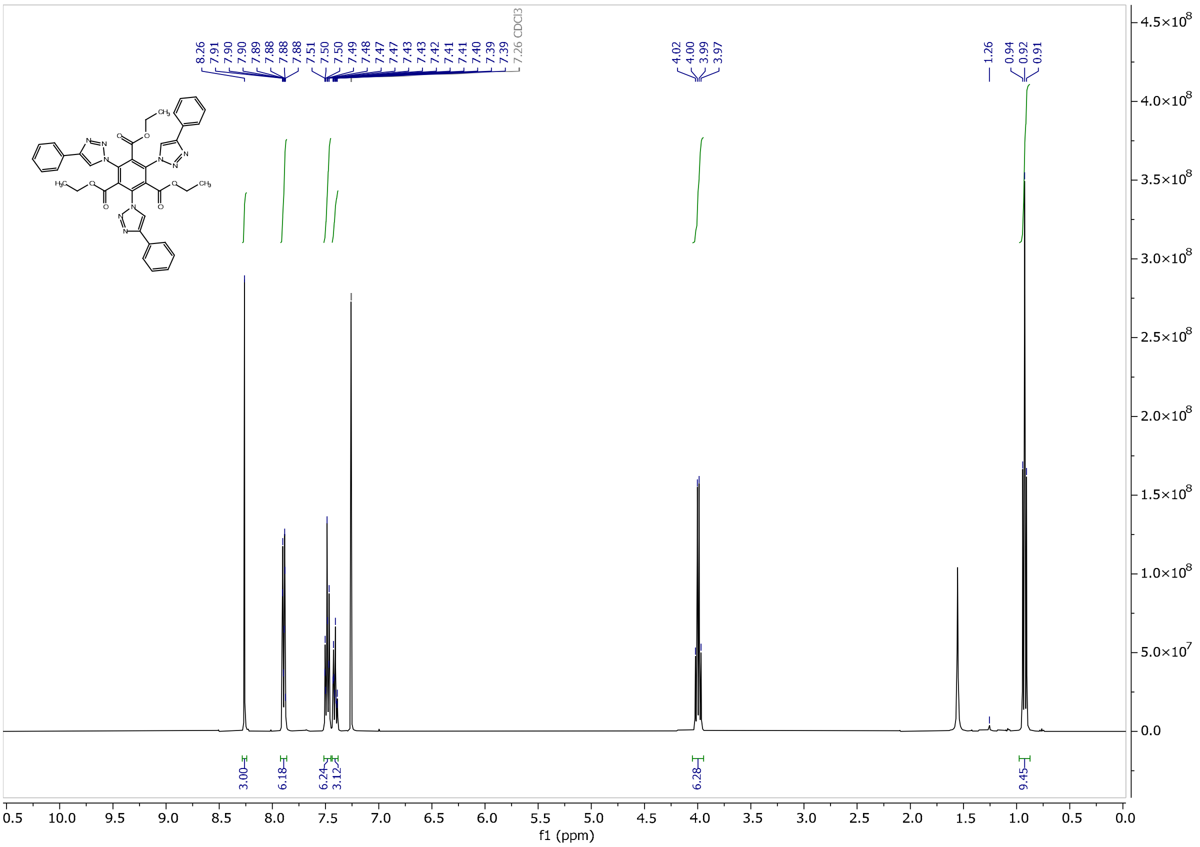

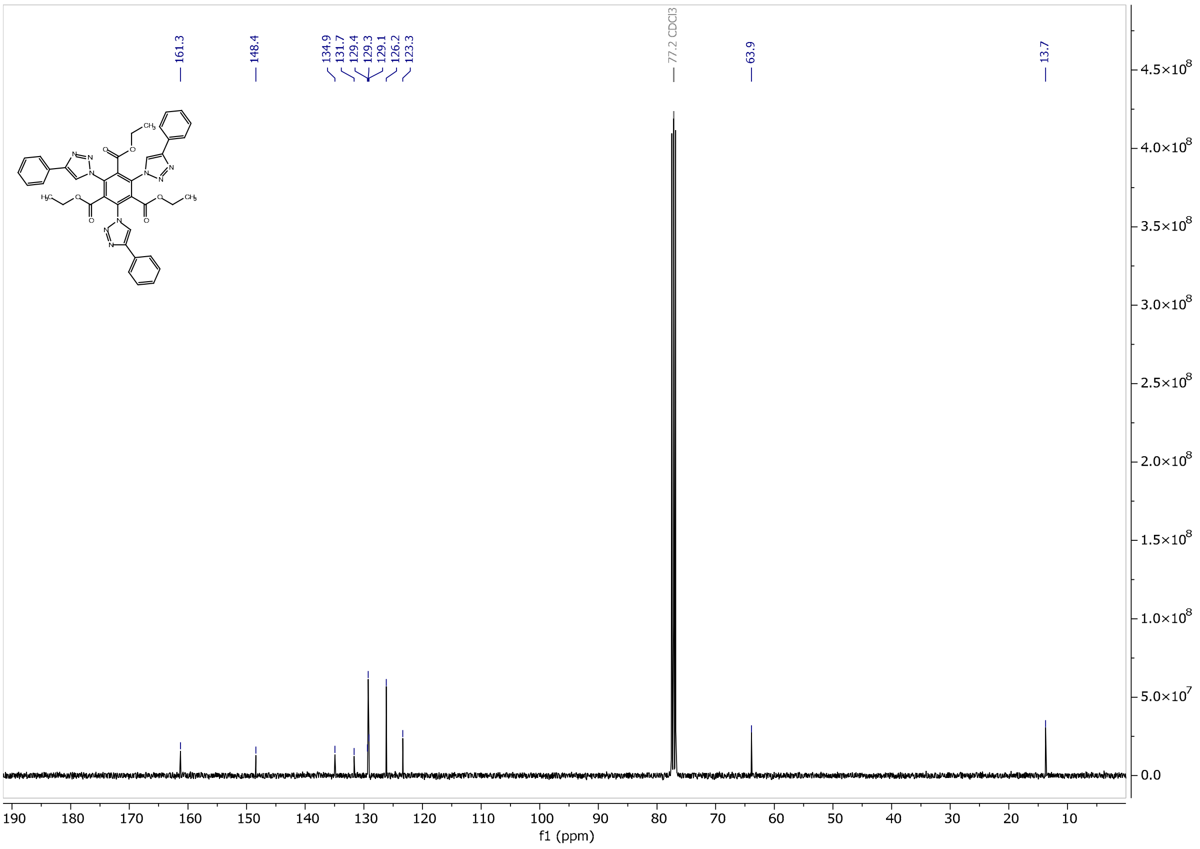

*Compound **7a***

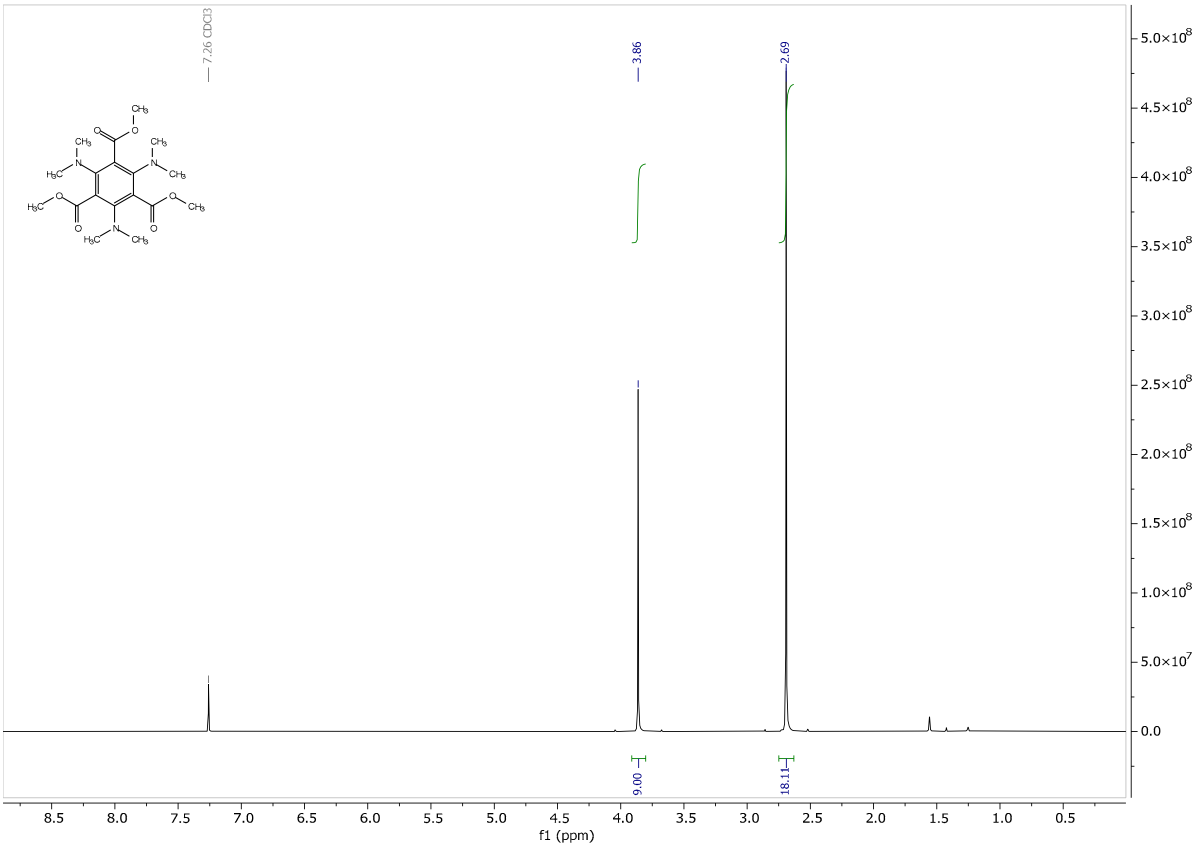
7.26 CDCl3
3.86
2.69
9.00
18.11
f1 (ppm)

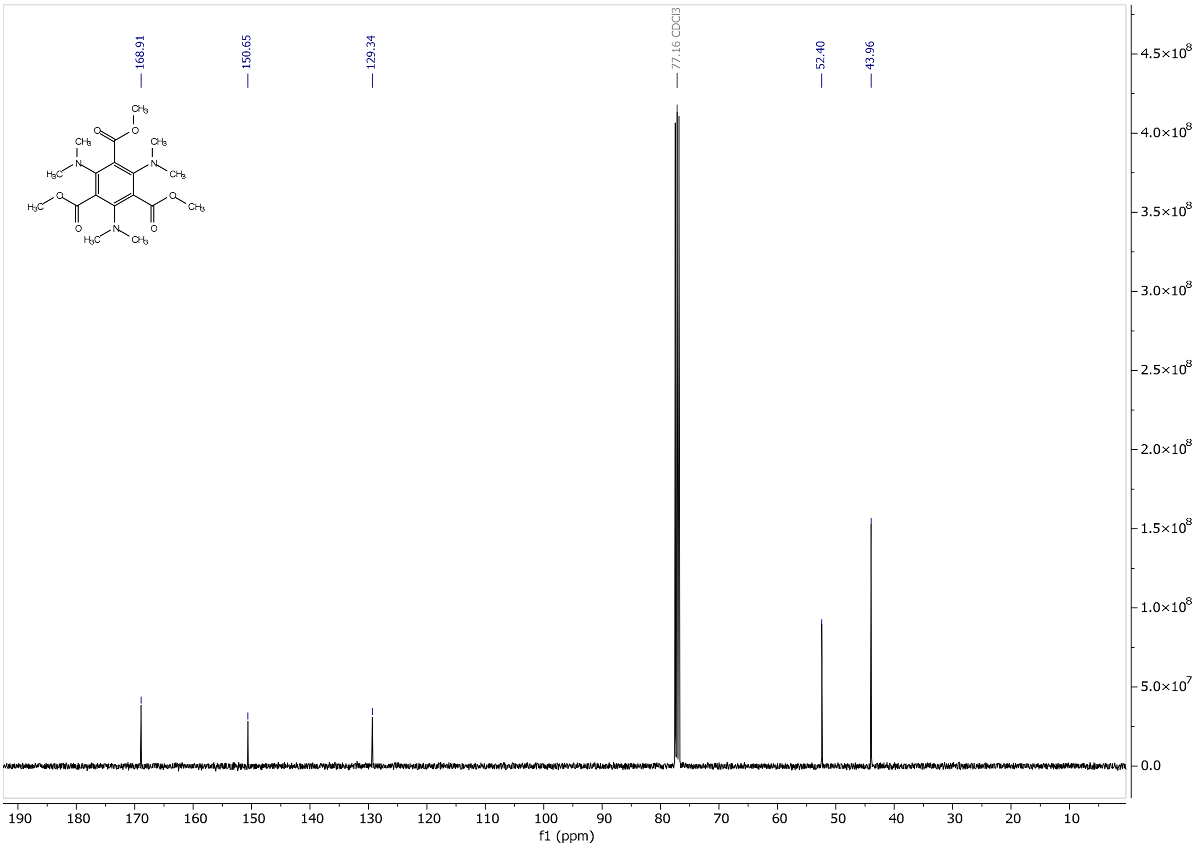
168.91
150.65
129.34
77.16 CDCl3
52.40
43.96
f1 (ppm)

*Compound **7b***

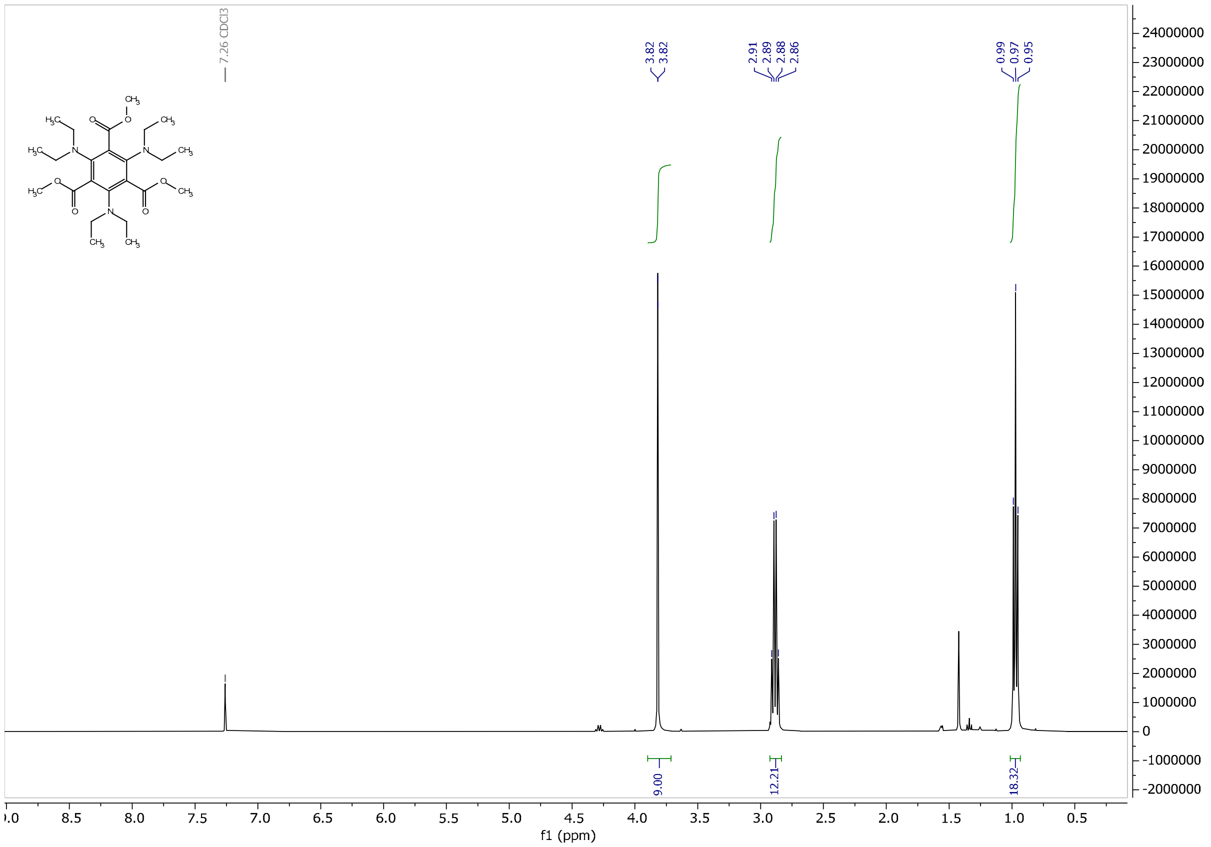
7.26 CDCl3
3.82
3.82
2.91
2.89
2.88
2.86
0.99
0.97
0.95
9.00
12.21
18.32
f1 (ppm)

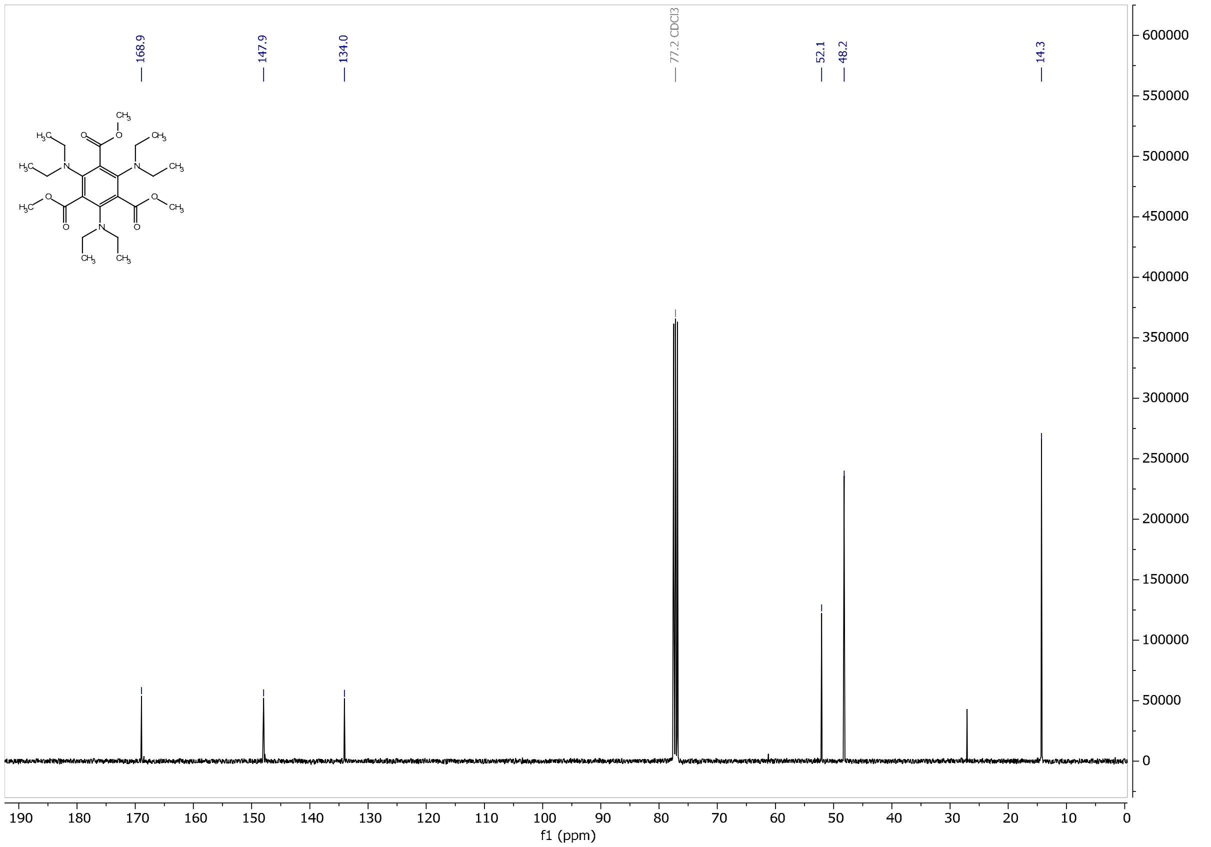
168.9
147.9
134.0
77.2 CDCl3
52.1
48.2
14.3
f1 (ppm)

Compound ***7c***

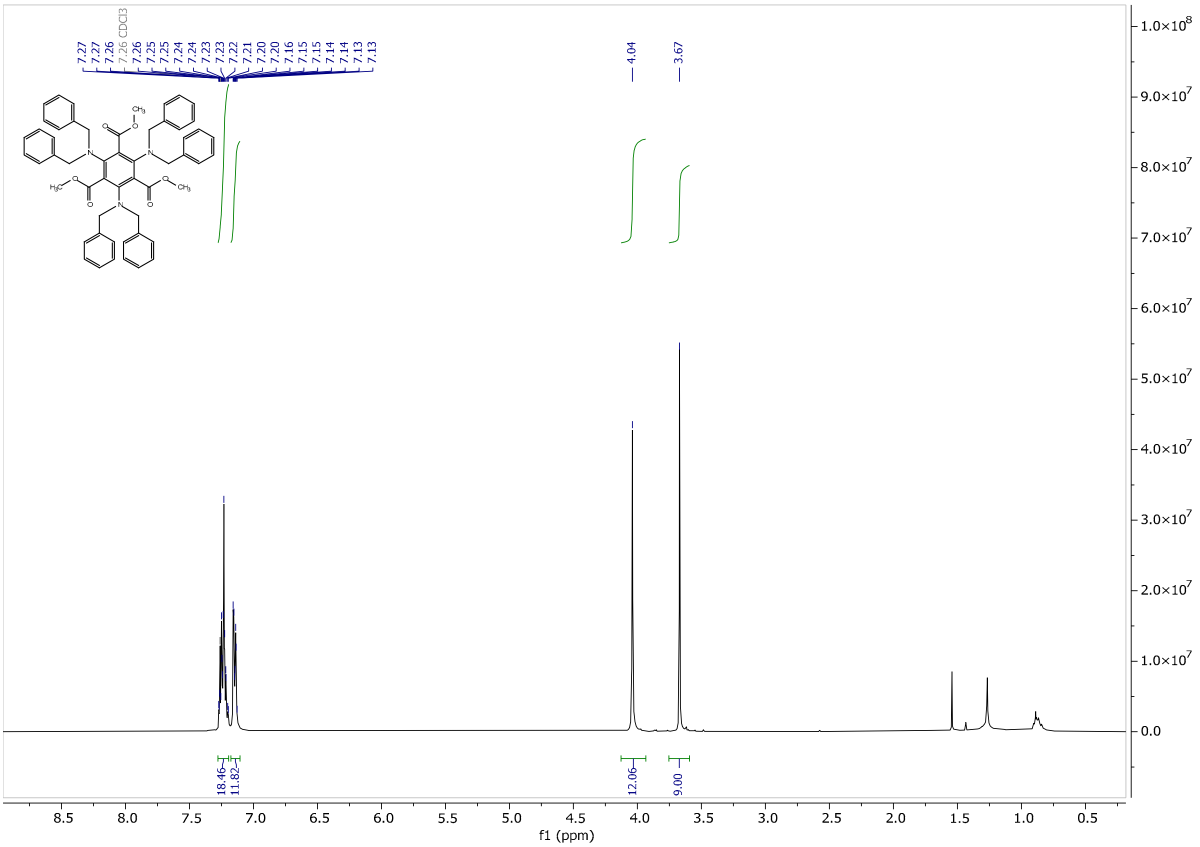
7.27
7.27
7.26
7.26 CDCl3
7.26
7.25
7.25
7.24
7.24
7.23
7.23
7.22
7.21
7.20
7.20
7.16
7.15
7.15
7.14
7.14
7.13
7.13
4.04
3.67
18.46
11.82
12.06
9.00
f1 (ppm)

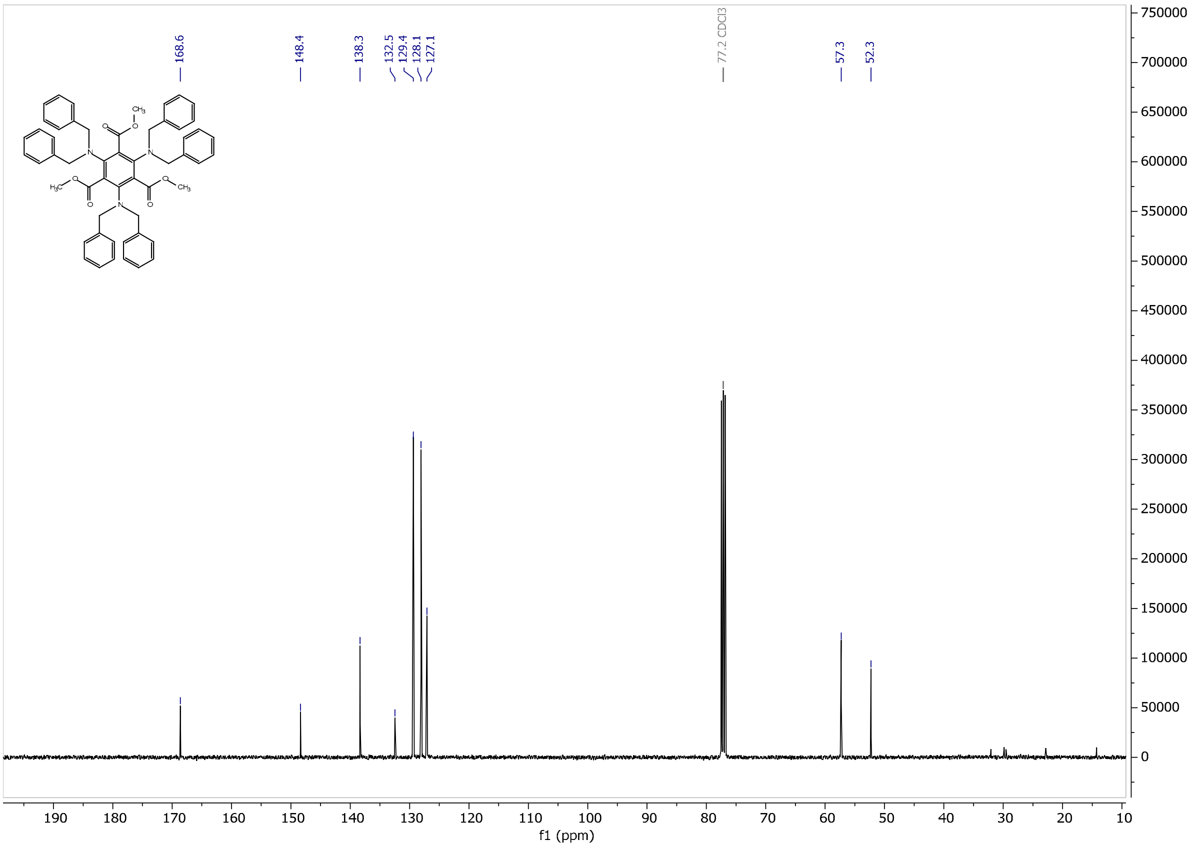
168.6
148.4
138.3
132.5
129.4
128.1
127.1
77.2 CDCl3
57.3
52.3
f1 (ppm)

References

1. Including heterocycles
2. Tamura, Y.; Takezawa, H.; Fujita, M., A Double-Walled Knotted Cage for Guest-Adaptive Molecular Recognition. *J. Am. Chem. Soc.* **2020**, *142*, 5504–5508.
3. Thomaides, J.; Maslak, P.; Breslow, R., Electron-rich hexasubstituted benzene derivatives and their oxidized cation radicals, dications with potential triplet ground states, and polycations. *J. Am. Chem. Soc.* **1988**, *110*, 3970–3979.
4. Omenat, A.; Barbera, J.; Serrano, J. L.; Houbrechts, S.; Persoons, A., Columnar liquid crystals with highly polar groups. Evaluation of the nonlinear optical properties. *Adv. Mater. (Weinheim, Ger.)* **1999**, *11*, 1292–1295.
5. Zhang, W.; Horoszewski, D.; Decatur, J.; Nuckolls, C., A Folded, Secondary Structure in Step-Growth Oligomers from Covalently Linked, Crowded Aromatics. *J. Am. Chem. Soc.* **2003**, *125*, 4870–4873.
6. Bushey, M. L.; Hwang, A.; Stephens, P. W.; Nuckolls, C., Enforced Stacking in Crowded Arenes. *J. Am. Chem. Soc.* **2001**, *123*, 8157–8158.
7. Bucsis, L.; Friedrich, K., Polycyanobenzenes, V. An easy access to tetracyanohydroquinone. Preparation of the 1,4-dihalotetracyanobenzenes. *Chem. Ber.* **1976**, *109*, 2462–2468.
8. Friedrich, K.; Oeckl, S., Polycyanobenzenes. III. Reactions of hexacyanobenzene with alcohols. Pentacyanophenyl ethers and polycyano-substituted Meisenheimer complexes. *Chem. Ber.* **1973**, *106*, 3796–3802.
9. Howard, S. T.; Krygowski, T. M.; Ciesielski, A.; Wisiorowski, M., Angular group-induced bond alternation II. The magnitude and the nature of the effect and its application to polynuclear benzenoid systems. *Tetrahedron* **1998**, *54*, 3533–3548.
10. Bayard, E.; Hamel, S.; Rochefort, A., Hydrogen bonding and π-stacking in highly organized arenes-based molecular wire. *Org. Electron.* **2006**, *7*, 144–154.
11. Bushey, M. L.; Hwang, A.; Stephens, P. W.; Nuckolls, C., The Consequences of chirality in crowded arenes-macromolecular helicity, hierarchical ordering, and directed assembly. *Angew. Chem., Int. Ed.* **2002**, *41*, 2828–2831.
12. Nguyen, T.-Q.; Bushey, M. L.; Brus, L. E.; Nuckolls, C., Tuning Intermolecular Attraction to Create Polar Order and One-Dimensional Nanostructures on Surfaces. *J. Am. Chem. Soc.* **2002**, *124*, 15051–15054.
13. Rochefort, A.; Bayard, E.; Hadj-Messaoud, S., Competitive hydrogen bonding in π-stacked oligomers. *Adv. Mater. (Weinheim, Ger.)* **2007**, *19*, 1992–1995.
14. Halder, A.; Ghosh, M.; Khayum M, A.; Bera, S.; Addicoat, M.; Sasmal, H. S.; Karak, S.; Kurungot, S.; Banerjee, R., Interlayer Hydrogen-Bonded Covalent Organic Frameworks as High-Performance Supercapacitors. *J. Am. Chem. Soc.* **2018**, *140*, 10941–10945.
15. Barluenga, J.; González, J. M.; García-Martín, M. A.; Campos, P. J., Polyiodination on benzene at room temperature a regioselective synthesis of derivatives. *Tetrahedron Lett.* **1993**, *34*, 3893–3896.
16. Akimov, A.; Masitov, A.; Korchagin, D.; Chapyshev, S.; Misochko, E.; Savitsky, A., W-band EPR studies of high-spin nitrenes with large spin-orbit contribution to zero-field splitting. *J. Chem. Phys.* **2015**, *143*, 084313/1–084313/6.
17. Mendez-Vega, E.; Mieres-Perez, J.; Chapyshev, S. V.; Sander, W., Persistent Organic High-Spin Trinitrenes. *Angew. Chem., Int. Ed.* **2019**, *58*, 12994–12998.
18. Misochko, E. Y.; Akimov, A. V.; Masitov, A. A.; Korchagin, D. V.; Aldoshin, S. M.; Chapyshev, S. V., Matrix isolation ESR spectroscopy and magnetic anisotropy of D3h symmetric septet trinitrenes. *J. Chem. Phys.* **2013**, *138*, 204317/1-204317/6.
19. Misochko, E. Y.; Akimov, A. V.; Mazitov, A. A.; Korchagin, D. V.; Chapyshev, S. V., Magnetic anisotropy parameters of matrix-isolated septet 1,3,5-trinitreno-2,4,6-trichlorobenzene. *Russ. Chem. Bull.* **2012**, *61*, 2218–2224.
20. Misochko, E. Y.; Akimov, A. V.; Mazitov, A. A.; Korchagin, D. V.; Yakushchenko, I. K.; Chapyshev, S. V., Magnetic anisotropy parameters of matrix-isolated septet 2,4,6-tribromo-1,3,5-trinitrenobenzene. *Russ. Chem. Bull.* **2015**, *64*, 87–91.
21. Misochko, E. Y.; Masitov, A. A.; Akimov, A. V.; Korchagin, D. V.; Chapyshev, S. V., Heavy Atom Effect on Magnetic Anisotropy of Matrix-Isolated Monobromine Substituted Septet Trinitrene. *J. Phys. Chem. A* **2015**, *119*, 2413–2419.
22. Rodionov, V. I.; Vaganova, T. A.; Malykhin, E. V., Selective mono- and diamination of some polyhalogenbenzenes in anhydrous ammonia. *J. Fluorine Chem.* **2015**, *180*, 98–102.
23. Takase, M.; Narita, T.; Fujita, W.; Asano, M. S.; Nishinaga, T.; Benten, H.; Yoza, K.; Mullen, K., Pyrrole-Fused Azacoronene Family: The Influence of Replacement with Dialkoxybenzenes on the Optical and Electronic Properties in Neutral and Oxidized States. *J. Am. Chem. Soc.* **2013**, *135*, 8031–8040.
24. Landenberger, K. B.; Bolton, O.; Matzger, A. J., Two Isostructural Explosive Cocrystals with Significantly Different Thermodynamic Stabilities. *Angew. Chem., Int. Ed.* **2013**, *52*, 6468–6471.

25. Reynaerts, R.; Minoia, A.; Gali, S. M.; Daukiya, L.; Van Velthoven, N.; De Vos, D.; Lazzaroni, R.; Mali, K. S.; De Feyter, S., Coplanar versus Noncoplanar Carboxyl Groups: The Influence of Sterically Enforced Noncoplanarity on the 2D Mixing Behavior of Benzene Tricarboxylic Acids. *J. Phys. Chem. C* **2020**, *124*, 24874–24882.
26. Hulvey, Z.; Vlaisavljevich, B.; Mason, J. A.; Tsivion, E.; Dougherty, T. P.; Bloch, E. D.; Head-Gordon, M.; Smit, B.; Long, J. R.; Brown, C. M., Critical Factors Driving the High Volumetric Uptake of Methane in Cu3(btc)2. *J. Am. Chem. Soc.* **2015**, *137*, 10816–10825.
27. You, W.; Liu, Y.; Howe, J. D.; Tang, D.; Sholl, D. S., Tuning Binding Tendencies of Small Molecules in Metal-Organic Frameworks with Open Metal Sites by Metal Substitution and Linker Functionalization. *J. Phys. Chem. C* **2018**, *122*, 27486–27494.
28. Krautwurst, J.; Smets, D.; Lamann, R.; Ruschewitz, U., How Does the Fluorination of the Linker Affect the Stability of Trimesate-Based Coordination Polymers and Metal-Organic Frameworks? *Inorg. Chem.* **2019**, *58*, 8622–8632.
29. Bushey, M. L.; Nguyen, T.-Q.; Nuckolls, C., Synthesis, Self-Assembly, and Switching of One-Dimensional Nanostructures from New Crowded Aromatics. *J. Am. Chem. Soc.* **2003**, *125*, 8264–8269.
30. Oakdale, J. S.; Sit, R. K.; Fokin, V. V., Ruthenium-Catalyzed Cycloadditions of 1-Haloalkynes with Nitrile Oxides and Organic Azides: Synthesis of 4-Haloisoxazoles and 5-Halotriazoles. *Chem. - Eur. J.* **2014**, *20*, 11101–11110.
31. Rieser, J.; Ismail, N.; Abou-Elenien, G.; Wallenfels, K., Mono-, bis- and trishydrazo, and -azo compounds in the tricyanobenzene series. I. Preparation and properties of the title compounds. *Liebigs Ann. Chem.* **1981**, (9), 1586–1597.
32. Alahakoon, S.; Tan, K.; Pandey, H.; Diwakara, S. D.; McCandless, G. T.; Grinffiel, D. I.; Durand-Silva, A.; Thonhauser, T.; Smaldone, R. A., 2D-Covalent Organic Frameworks with Interlayer Hydrogen Bonding Oriented through Designed Nonplanarity. *J. Am. Chem. Soc.* **2020**, *142*, 12987–12994.
33. Jiang, H.; Jia, J.; Shkurenko, A.; Chen, Z.; Adil, K.; Belmabkhout, Y.; Weselinski, L. J.; Assen, A. H.; Xue, D.-X.; O'Keeffe, M.; Eddaoudi, M., Enriching the Reticular Chemistry Repertoire: Merged Nets Approach for the Rational Design of Intricate Mixed-Linker Metal-Organic Framework Platforms. *J. Am. Chem. Soc.* **2018**, *140*, 8858–8867.
34. Cady, H. H.; Larson, A. C., The crystal structure of 1,3,5-triamino-2,4,6-trinitrobenzene. *Acta Crystallogr.* **1965**, *18*, 485–496.
35. Desimoni, G.; Gamba Invernizzi, A.; Quadrelli, P.; Righetti, P. P., Copper(II) in organic synthesis. IX. The copper(II)-catalyzed Michael reaction as a route to polysubstituted benzene derivatives. *Gazz. Chim. Ital.* **1991**, *121*, 483–485.
36. Stoessel, P.; Buesing, A.; Breuning, E.; Pflumm, C.; Parham, A. H.; Eberle, T.; Mujica-Fernaud, T. Heteroaromatic compounds for organic electroluminescent devices. WO2012095143A1, 2012.
37. Klebe, J. F., New silyl donors. *J. Am. Chem. Soc.* **1964**, *86*, 3399–3400.
38. Wallenfels, K.; Witzler, F.; Friedrich, K., 1,3,5-Trichloro-2,4,6-tricyanobenzene and 1,3,5-trifluoro-2,4,6-tricyanobenzene. *Tetrahedron* **1967**, *23*, 1845–1855.
39. Wasserman, E.; Schueller, K.; Yager, W. A., E.P.R. detection of the septet ground state of a trinitrene. *Chem. Phys. Lett.* **1968**, *2*, 259–260.
40. Ledoux, I.; Zyss, J.; Siegel, J. S.; Brienne, J.; Lehn, J. M., Second-harmonic generation from nondipolar noncentrosymmetric aromatic charge-transfer molecules. *Chem. Phys. Lett.* **1990**, *172*, 440–444.
41. Alandini, N.; Buzzetti, L.; Favi, G.; Schulte, T.; Candish, L.; Collins, K. D.; Melchiorre, P., Amide Synthesis by Nickel/Photoredox-Catalyzed Direct Carbamoylation of (Hetero)Aryl Bromides. *Angew. Chem., Int. Ed.* **2020**, *59*, 5248–5253.
42. Hu, Y.; Yin, J.; Chaitanya, K.; Ju, X.-H., Theoretical investigation on charge transfer properties of 1,3,5-tripyrrolebenzene (TPB) and its derivatives with electron-withdrawing substituents. *Croat. Chem. Acta* **2016**, *89*, 81–90.
43. Lee, G. H.; Kwon, D. Y.; Kim, Y. S., Study of cyanobenzene derivatives for thermally activated delayed fluorescence emitters. *J. Nanosci. Nanotechnol.* **2016**, *16*, 11453–11456.
44. Shu, Y.; Levine, B. G., Simulated evolution of fluorophores for light emitting diodes. *J. Chem. Phys.* **2015**, *142*, 104104/1-104104/11.
45. Tsipis, A. C., Interaction of Elemental Mercury with a Diverse Series of π-Organic Substrates Probed by Computational Methods: Is Mercury Fixation Possible? *ACS Earth Space Chem.* **2018**, *2*, 451–461.
46. Bailey, A. S.; Case, J. R., 4,6-Dinitrobenzofuroxan, nitrobenzodifuroxan, and benzotrifuroxan. A new series of complex-forming reagents for aromatic hydrocarbons. *Tetrahedron* **1958**, *3*, 113–131.
47. Bagal, M. L.; Ishchenko, M. A.; Nikolaev, V. D., Sterically hindered carboxylic acid esters. *Russ. J. Org. Chem.* **1997**, *33*, 1731–1738.
48. Radulescu, D.; Novac, L.; Petreanu, I.; Popa, S., Trinitrobenzene derivatives. Properties of the nitro group. *Bul. Soc. Stiinte Cluj* **1939**, *9*, 215–254.

49. Goncharov, T. K.; Dubikhin, V. V.; Nazin, G. M.; Prokudin, V. G., Effect of oxygen-containing substituents on the stability of compounds with trinitromethyl groups. *Khim. Fiz.* **2006**, *25*, 33–37.
50. Becker, M.; Voss, K.; Villinger, A.; Schulz, A., An efficient route to 1,3,5-triazido-2,4,6-tricyanobenzene. *Z. Naturforsch., B: J. Chem. Sci.* **2012**, *67*, 643–649.
51. Schneider, R., Action of sulphur chloride on silver cyanide. *Jour. prakt. Chem.* 32, 187.
52. Wasserman, E.; Schueller, K.; Yager, W. A., ESR identification of septet ground state a Trinitrens. *Chem. Zentralbl.* **1969**, *140*, 94.
53. Lin, Y.; Wu, X.; Feng, S.; Jiang, G.; Luo, J.; Zhou, S.; Vrijmoed, L. L. P.; Jones, E. B. G.; Krohn, K.; Steingroever, K.; Zsila, F., Five Unique Compounds: Xyloketals from Mangrove Fungus Xylaria sp. from the South China Sea Coast. *J. Org. Chem.* **2001**, *66*, 6252–6256.
54. Pettigrew, J. D.; Cadieux, J. A.; So, S. S. S.; Wilson, P. D., Phenylboronic Acid Mediated Triple Condensation Reactions of Phloroglucinol and Unsaturated Carbonyl Compounds. *Org. Lett.* **2005**, *7*, 467–470.
55. Bosch, E.; Barnes, C. L., Triangular Halogen-Halogen-Halogen Interactions as a Cohesive Force in the Structures of Trihalomesitylenes. *Cryst. Growth Des.* **2002**, *2*, 299–302.
56. Shishkin, O. V.; Medvediev, V. V.; Zubatyuk, R. I., Supramolecular architecture of molecular crystals possessing shearing mechanical properties: columns versus layers. *CrystEngComm* **2013**, *15*, 160–167.
57. Abraham, F.; Kress, R.; Smith, P.; Schmidt, H.-W., A New Class of Ultra-Efficient Supramolecular Nucleating Agents for Isotactic Polypropylene. *Macromol. Chem. Phys.* **2013**, *214*, 17–24.
58. Shuai, Z.; Ramasesha, S.; Bredas, J. L., Nonlinear optical properties of nitro-aniline and methyl-aniline compounds - an exact correction vector INDO-SDCI study. *Chem. Phys. Lett.* **1996**, *250*, 14–18.
59. Walsdorff, C.; Saak, W.; Pohl, S., A new preorganized tridentate ligand bearing three indolethiolate groups. Preparation of 3:1 subsite-differentiated Fe4S4 clusters. *J. Chem. Soc., Dalton Trans.* **1997**, (11), 1857–1861.
60. Cho, B. R.; Lee, S. J.; Lee, S. H.; Son, K. H.; Kim, Y. H.; Doo, J.-Y.; Lee, G. J.; Kang, T. I.; Lee, Y. K.; Cho, M.; Jeon, S.-J., Octupolar Crystals for Nonlinear Optics: 1,3,5-Trinitro-2,4,6-tris(styryl)benzene Derivatives. *Chem. Mater.* **2001**, *13*, 1438–1440.
61. Chowdhury, H.; Chatterjee, N.; Goswami, A., An Eco-Friendly Route to N-Arylindoles by Iron-Catalyzed [2+2+2] Cycloaddition of Diynes with (Indol-1-yl)alkynes. *Eur. J. Org. Chem.* **2015**, *2015*, 7735–7742.
62. Vandendriessche, S.; Van Cleuvenbergen, S.; Willot, P.; Hennrich, G.; Srebro, M.; Valev, V. K.; Koeckelberghs, G.; Clays, K.; Autschbach, J.; Verbiest, T., Giant Faraday Rotation in Mesogenic Organic Molecules. *Chem. Mater.* **2013**, *25*, 1139–1143.
63. Molina-Ontoria, A.; Zimmermann, I.; Garcia-Benito, I.; Gratia, P.; Roldan-Carmona, C.; Aghazada, S.; Graetzel, M.; Nazeeruddin, M. K.; Martin, N., Benzotrithiophene-Based Hole-Transporting Materials for 18.2% Perovskite Solar Cells. *Angew. Chem., Int. Ed.* **2016**, *55*, 6270–6274.
64. Wolff, J. J.; Siegler, F.; Matschiner, R.; Wortmann, R., Optimized two-dimensional NLO chromophores with a threefold symmetry axis. *Angew. Chem., Int. Ed.* **2000**, *39*, 1436–1439.
65. Nguyen, T.-Q.; Martel, R.; Avouris, P.; Bushey, M. L.; Brus, L.; Nuckolls, C., Molecular Interactions in One-Dimensional Organic Nanostructures. *J. Am. Chem. Soc.* **2004**, *126*, 5234–5242.
66. Tulevski, G. S.; Bushey, M. L.; Kosky, J. L.; Ruter, S. J. T.; Nuckolls, C., Assembling dimeric π stacks on gold surfaces by using three-dimensional lock-and-key receptors. *Angew. Chem., Int. Ed.* **2004**, *43*, 1836–1839.
67. Estey, P.; Bubar, A.; Decken, A.; Calhoun, L.; Eisler, S., Synthesis and Photochemical Isomerization of a Propeller-Shaped Molecular Switch. *Chem. - Eur. J.* **2013**, *19*, 16204–16208.
68. Anthony, J. E.; Khan, S. I.; Rubin, Y., 1,3,5 / 2,4,6-Differentiated hexaalkynylbenzenes: absorption and fluorescence properties of a D3h-symmetric donor-substituted system. *Tetrahedron Lett.* **1997**, *38*, 3499–3502.
69. Bruns, D.; Miura, H.; Vollhardt, K. P. C.; Stanger, A., En Route to Archimedene: Total Synthesis of C3h-Symmetric [7]Phenylene. *Org. Lett.* **2003**, *5*, 549–552.
70. Katoono, R.; Kawai, H.; Ohkita, M.; Fujiwara, K.; Suzuki, T., A C3-symmetric chiroptical molecular propeller based on hexakis(phenylethynyl)benzene with a threefold terephthalamide: stereospecific propeller generation through the cooperative transmission of point chiralities on the host and guest upon complexation. *Chem. Commun. (Cambridge, U. K.)* **2013**, *49*, 10352–10354.
71. Yang, X.; Yuan, L.; Chen, Z.; Liu, Z.; Miao, Q., A Trefoil Macrocycle Synthesized by 3-Fold Benzannulation. *Org. Lett.* **2018**, *20*, 6952–6956.
72. Annelated systems are excluded
